# Measurement of Physicochemical Properties and CO_2_, N_2_, Ar, O_2_, and H_2_O Unary Adsorption Isotherms of Purolite A110 and Lewatit VP OC 1065 for Application in Direct Air Capture

**DOI:** 10.1021/acs.jced.3c00401

**Published:** 2023-10-11

**Authors:** May-Yin Ashlyn Low, David Danaci, Hassan Azzan, Robert T. Woodward, Camille Petit

**Affiliations:** † Barrer Centre, Department of Chemical Engineering, Imperial College London, London SW7 2AZ, U.K.; ‡ Institute of Materials Chemistry & Research,University of Vienna, 1090 Vienna, Austria

## Abstract

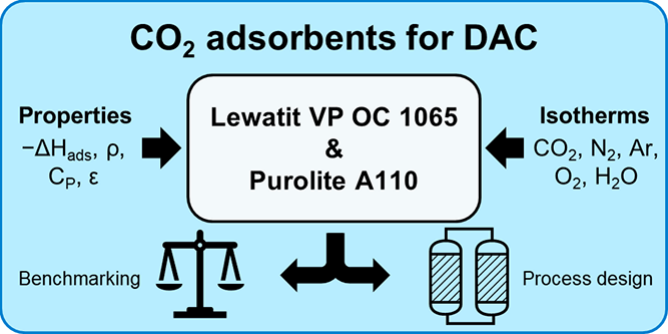

Direct air capture (DAC) using solid adsorbents has gained significant attention as a carbon dioxide removal (CDR) technology to help limit global temperature rise to below 2 °C. One large area of focus is the development of new adsorbent materials for DAC. However, the necessary data needed to employ these materials in process models for adsorbent screening are rarely available. Here, we showcase Purolite A110, a commercially available amine-functionalized polymeric resin, as a new candidate adsorbent for DAC and compare its properties to a current benchmark, Lewatit VP OC 1065. For both materials, we report their chemical features and composition, skeletal, particle, and bed density, total pore volume, particle porosity, BET area, thermal stability, and specific heat capacity. We determine their equilibrium sorption properties by measuring the volumetric CO_2_ isotherms at 288, 298, 308, 333, 343, 353, and 393 K, N_2_ and H_2_O isotherms at 288, 298, and 308 K, and Ar and O_2_ isotherms at 298 K. For CO_2_, N_2_, and H_2_O, we also present the corresponding isotherm model fitting parameters and heats of adsorption. These data can help facilitate process modeling and optimization studies to properly assess these adsorbents at scale.

## 1 Introduction

The 2015 Paris Agreement saw 196 countries commit to limiting global temperature rise to below 2 °C, and preferably below 1.5 °C. According to recent reports by the Intergovernmental Panel on Climate Change (IPCC), this scenario can only be achieved with carbon dioxide removal (CDR) technologies.^1^ The use of solid adsorbents to remove CO_2_ directly from the atmosphere, i.e., adsorption-based direct air capture (DAC), is a CDR technology that is actively being investigated. It is a challenging process due to the ultradilute concentration of CO_2_ in the air (∼0.04%_vol_ or 0.04 kPa) and varying ambient conditions (e.g., temperature and relative humidity), but it offers a route toward long-term carbon storage and allows the amount of CO_2_ captured to be quantitatively measured.

A number of commercial adsorption-based DAC plants are currently operational, amounting to a CO_2_ capture capacity on the order of ca. 10 kt year^–1^.^2^ However, this only equates to 0.0002% of the CO_2_ removal goal of 4.8 Gt year^–1^ by 2050, stipulated in the “below 2 °C” scenario (of which 2.7 Gt year^–1^ is thought to be removed by engineered CDR technologies such as DAC).^3^ Hence, much work is still needed for the scale-up of these DAC processes. One aspect actively being researched is the development of new adsorbent materials. From our recent work,^2^ we determined that over 200 adsorbents had been proposed for DAC in the literature since 2016, including silicas, metal oxides, metal–organic frameworks (MOFs), zeolites, and polymers. Some of the most reported metrics were the adsorbents’ CO_2_ uptake at 0.04 kPa, or a CO_2_ isotherm at a single temperature (usually 298 K). However, works such as those by Khurana and Farooq,^4^ Rajagopalan et al.,^5^ and Farmahini et al.^6^ have shown that simple metrics such as these are not sufficient for adsorbent screening. Instead, performance indicators determined from process modeling and optimization, such as purity, recovery, productivity, and energy consumption, should be used. In the case of DAC, these are especially important to determine the net CO_2_ removal achieved by the process. To conduct process-scale evaluation, various material properties are needed, as outlined in Table 1.

**Table 1:** Summary of Adsorbate and Adsorbent Properties Required in the Modeling of an Adsorption-Based DAC Process^a^

material property required	process modeling output
equilibrium isotherms considering coadsorption for ALL adsorbing components in ambient air at multiple temperatures	total amount adsorbed and desorbed
overall mass transfer coefficient	rate of mass transfer and adsorption
density (skeletal, particle, and bed)	mass of solid and volume of process contactor required
porosity (particle and bed) derived from pore volume estimation	total volume of gas in vessel space and mass of adsorbent
heat capacity (adsorbent and adsorbed phase)	heat required to perform desorption
heat of adsorption (of all adsorbing components)	required energy input for desorption and process temperature changes
overall heat transfer coefficient (related to thermal conductivity of the adsorbent)	rate of heat transfer in material

a Adapted from Low et al. (open access).^2^

It is extremely rare to find adsorbents in the literature with all of the necessary material properties reported, even when considering numerous sources. Of the more than 200 proposed DAC adsorbents in recent years, only two materials, zeolite 13X and Lewatit VP OC 1065, had sufficient data required for process modeling. These include: CO_2_, N_2_, and H_2_O isotherms measured at three or more temperatures from which isotherm model fitting parameters and heats of adsorption could be determined; bed density; particle density; skeletal density and/or total pore volume; heat capacity; thermal conductivity; and mass transfer coefficients at low CO_2_ composition. While 13X is a well-known commercial benchmark for postcombustion carbon capture, it has a relatively low CO_2_ adsorption capacity of 0.2–0.3 mmol g^–1^ at 0.04 kPa CO_2_ and 298 K.^7,8^

Lewatit VP OC 1065 (referred to as Lewatit from here) is a commercially available polymeric resin functionalized with primary amines and is believed to be very similar to the adsorbents used by the company Climeworks,^9^ one of the current commercial operators of adsorption-based DAC. It has a comparably high CO_2_ capacity of 1.0 mmol g^–1^ at 0.04 kPa CO_2_ and 298 K under dry conditions.^10^ This is due to its chemisorbent nature, where two amine groups react with and capture CO_2_ to form ammonium carbamate^11^ (see eq 1).
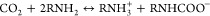
1

In recent years, Lewatit has gained attention for its application in DAC, and it is often regarded as a benchmark adsorbent. The current literature consists of investigating the performance of Lewatit using different cycle processes^9,10,12−14^ and contactor designs,^15−20^ the impact of climate variations,^21,22^ a few pilot-scale investigations,^23,24^ and some kinetic studies at low CO_2_ compositions.^9,25,26^

In addition to gathering the data to fully evaluate the potential of adsorbents at scale, expanding the group of easily accessible commercial materials for DAC to cover the wide range of climate conditions will help accelerate the deployment of adsorption-based DAC technologies. In the field of polymeric resins, Shu et al. investigated a commercial resin (AmberLite HPR4800 OH) using pH swing regeneration in an electrochemical cell,^27^ which had a CO_2_ adsorption capacity of 1.76 mmol g^–1^ under humid conditions at 0.04 kPa CO_2_ and 298 K. The particle and bed densities of the adsorbent are available from the manufacturer,^28^ but other properties needed for process modeling (see Table 1) were not reported in this study. Elfving et al. studied an undisclosed resin supplied by Oy Hydrocell Ltd. under various process cycles,^29^ regeneration conditions,^30^ and dry versus humid conditions.^31,32^ The adsorbent consists of a polystyrene matrix functionalized with primary amines, like Lewatit, with a dry CO_2_ adsorption capacity of 0.8 mmol g^–1^ at 0.04 kPa CO_2_ and 298 K. Single-component CO_2_ and H_2_O isotherms at multiple temperatures, as well as CO_2_–H_2_O coadsorption isotherms, have been measured. The corresponding isotherm equations and heats of adsorption were also reported along with the bed density, particle density, and specific heat capacity. Parvazinia et al. studied another amine-functionalized polymeric resin, Purolite A109, for CO_2_ capture at 2 kPa and 298 K,^33^ which had a CO_2_ adsorption capacity of 0.2 mmol g^–1^ at these conditions. More recently, Purolite A110, the successor of Purolite A109, appeared in the literature for the first time for DAC applications.^34^ However, the authors modified the material with a CuCl_2_ solution to form their Polyam–N-Cu^2+^ adsorbent, which would capture CO_2_ only under humid conditions via a carbonate reaction. This material was reported to have a CO_2_ adsorption capacity of 5.1 mmol g^–1^ at 0.04 kPa CO_2_, 298 K, and 50% RH. Under the same conditions, unmodified Purolite captured 1.8 mmol g^–1^ of CO_2_, which is comparable to Lewatit at similar conditions (∼0.035 kPa, 298 K, 55% RH).^9^ However, the necessary data needed for process-scale evaluation, such as equilibrium isotherms and heats of adsorption, have not been measured.

In this study, we propose unmodified Purolite A110 (referred to as Purolite from here) as a new candidate benchmark adsorbent for DAC and compare its properties to those of the current benchmark, Lewatit. Our objective is to measure all the material and equilibrium sorption properties needed to perform process modeling and compare the results to that of Lewatit. For both Purolite and Lewatit, we report their chemical features, chemical composition, skeletal, particle, and bed densities, total pore volume, particle porosity, BET area, thermal stability, and specific heat capacity. To analyze their sorption properties, we measure CO_2_, N_2_, and H_2_O isotherms at multiple temperatures and Ar and O_2_ isotherms at a single temperature. For CO_2_, N_2_, and H_2_O, we also fit the corresponding isotherm models and determine heats of adsorption. While a mass transfer coefficient is also needed for process modeling, kinetic sorption properties are outside the scope of this study and are not reported.

## 2 Materials and Methods

### 2.1 Chemicals

Details of the commercial resins investigated in this study are provided in Table 2, along with chemicals used as reference materials throughout the study.

**Table 2:** Details of the Commercial Resins and Reference Chemicals Used in This Study

	Lewatit VP OC 1065	Purolite A110	Zeolite 13X	Silica–alumina	Aluminum oxide (γ-phase, 99.97%)
source	Sigma-Aldrich	Purolite	Micromeritics	Micromeritics	Alfa Aesar
product code	94136–100G-F	SR60210479	004–16843–00	004–16821–00	1344–28–1
lot number	BCCC7234	T014Z/21/6	9266–33	A-501–53	X30G032
matrix	styrene-divinylbenzene	styrene-divinylbenzene			
functional group	primary amine	primary amine			
exchange capacity (eq L^–1^)^a^	2.1	2.0			
water retention (wt %)^a^	52–58	60–66			
particle size (μm)^a^	300–1250	300–1200			
fines, <300 μm (vol %)^a^	3	1			
uniformity coefficient^a^	1.8	1.7			

a Information provided by the manufacturers on the technical data sheets.^35,36^

The purities of the gases used for the study are as follows. For gas sorption measurements: CO_2_ (BOC, N5.0 grade), N_2_ (BOC, N6.0 grade), Ar (BOC, N5.0 grade), and O_2_ (BOC, N5.0 grade). For pycnometry measurements: He (BOC, N5.0 grade). For elemental analysis: He (Messer, N5.0 grade). For thermogravimetric measurements: N_2_ (BOC, N4.8 grade) and compressed air (BOC, industrial grade). For differential scanning calorimetry (DSC) measurements: N_2_ (BOC, N5.2 grade).

### 2.2 Characterization of Chemical Features

Fourier transform infrared (FTIR) spectra of the resins were collected by using an Agilent Cary 630 FTIR spectrometer equipped with an attenuated total reflectance (ATR) accessory. Samples were first degassed ex situ at 393 K and 0.002 kPa for at least 12 h and then ground using a pestle and mortar before analysis. Sixteen spectra were measured per sample, and an average spectrum was obtained over the wavenumber range of 400–4000 cm^–1^ with a resolution of 2 cm^–1^.

Elemental analysis was performed using a Eurovector EA 3000 CHNS-O elemental analyzer on samples weighing between 0.75 and 3.0 mg, which were weighed into tin vials (4 × 6 mm) prior to measurement. Samples were degassed ex situ at 393 K and 0.002 kPa for at least 12 h prior to measurement, and each sample was run in triplicate. Accurate sample weighing was achieved using a microbalance (Sartorius, ME 5 OCE). The operating temperatures for combustion and reduction were 1273 and 1023 K, respectively. He was used as the carrier gas in all cases.

### 2.3 Characterization of Textural Properties and Density

N_2_ sorption isotherms at 77 K were measured using a Micromeritics 3Flex porosity analyzer. Samples were first degassed ex situ at 393 K and 0.002 kPa for at least 12 h and then degassed in situ at 393 K and 0.000002 kPa for 24 h. Approximately 100–200 mg of dried samples was used for the measurements. The specific surface area was determined using the Brunauer–Emmett–Teller (BET) method,^37^ with the Rouquerol criteria applied.^38^ The cumulative pore size distribution was determined using the density functional theory (DFT) model available in the 3Flex software, the “N2-DFT Model”, with a slit-shaped pore geometry kernel, and 0.10000 regularization.

Skeletal densities (ρ_skeletal_) were determined via helium pycnometry using a Micromeritics AccuPyc II 1340 pycnometer. Samples were first degassed at 393 K and 0.002 kPa for at least 12 h, and approximately 1.7 g of dried sample was used to fill a 3.5 cm^3^ sample holder. The samples were then exposed to 20 purge cycles of He dosing (∼134 kPa), followed by 10 measurement cycles under the same conditions. The skeletal density values presented in this study are the averages determined from 10 measurement cycles.

Bed densities (ρ_bed_) were measured by filling a 3D-printed cylindrical container (2.0 cm × 2.0 cm cube with a 1.6 cm diameter by 1.2 cm height cylindrical cavity; see Figure S1 for the image) with a dried sample (degassed at 393 K and 0.002 kPa for at least 12 h). The container was overfilled with the dried sample, tapped 20 times, and the excess swept off using a straight-edged spatula. The total weight of the container and sample was then measured on an analytical balance (OHAUS Adventurer Pro), from which the weight of the empty container was subtracted to obtain the mass of the sample. This was then divided by the known volume of the cylinder, determined by weighing the mass of water required to fill its cavity, to determine the final bed density. This procedure was repeated three times for each sample.

Once the skeletal and bed densities were determined, and assuming a spherical bed voidage (ε_bed_) of 0.36,^39^ the particle density (ρ_particle_), total pore volume (*V*_total_), and adsorbent voidage (ε_particle_) of each sample were calculated from eqs 2–4:
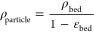
2
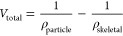
3
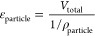
4

### 2.4 Characterization of Thermal Properties

Thermogravimetric analysis (TGA) of the resins was carried out using a Netzsch TG 209 F1 Libra thermal analyzer. Approximately 20 mg of the untreated sample was placed in an alumina crucible and subjected to heat treatment from room temperature to 1173 K at a ramp rate of 10 K min^–1^. This was done under either a protective N_2_ flow (100 mL min^–1^) or a mixture of air (100 mL min^–1^) and a protective N_2_ flow (20 mL min^–1^). A correction run with an empty alumina crucible and identical analysis conditions was performed prior to these measurements to account for buoyancy effects.

Specific heat capacities were measured using a PerkinElmer DSC 8000 differential scanning calorimeter following a methodology adapted from Moosavi et al.^40^ Samples were first degassed ex situ at 393 K and 0.002 kPa for at least 12 h and then ground into powder using a pestle and mortar before analysis. Approximately 6 mg of powder was placed in an aluminum DSC pan and crimped with a corresponding aluminum lid with a manually pierced pinhole. The sample pan was placed into the sample chamber, while an identical empty aluminum pan and lid (uncrimped) were placed into the reference chamber. The external chiller was set to 253 K, and the chambers were then subjected to the following temperature profile: isothermal at 293 K for 5 min, heating ramp to 393 K at a rate of 10 K min^–1^, and isothermal at 393 K for 15 min, all under a N_2_ flow of 40 mL min^–1^. This profile was repeated without opening the sample and reference chambers, until three overlapping heat flow curves were obtained. Prior to each series of measurements, an empty aluminum DSC pan and lid and a sapphire standard were used to obtain the baseline and reference curves, respectively. The same procedure as outlined above was used for these measurements, although the aluminum pans were not crimped. Heat flow curves for all measurements are shown in Figure S2. The specific heat capacity curves were then derived using the PerkinElmer DSC 8000 software using the last three overlapping heat flow curves for each component (baseline, reference, and sample). This methodology was verified by measuring the heat capacities of γ-alumina powder and the α-alumina (sapphire) disc provided by the DSC instrument manufacturer. Both experimental results were comparable to the literature values provided by the National Institute of Standards and Technology (NIST)^41^ (Figure S3). The measured specific heat capacity data for Lewatit and Purolite were fitted to a known specific heat capacity equation using Microsoft Excel 2016 Solver.

### 2.5 Analyses of Gas and Vapor Sorption Properties

Gas and vapor sorption isotherms were measured volumetrically using a Micromeritics 3Flex porosity analyzer for CO_2_ at 288, 298, 308, 333, 343, 353, and 393 K, for N_2_ and H_2_O at 288, 298, and 308 K, and for Ar and O_2_ at 298 K. Samples were first degassed ex situ at 393 K and 0.002 kPa for at least 12 h and then degassed in situ at 393 K and 0.000002 kPa for 24 h. For CO_2_ measurements, approximately 100–200 mg of dried sample was used, and fresh sample was used for each temperature measurement. For H_2_O measurements, approximately 100–200 mg of dried sample was used, and the same sample was used to measure all temperatures. For N_2_, Ar, and O_2_ measurements, approximately 1.0–1.5 g of dried sample was used, and the same sample was used for all measurements, which were done in the gas order listed. A glass stirring rod was also placed in each sample tube for these measurements to reduce the free space volume. For measurements at and below 308 K, temperature control was achieved by submerging the sample tubes in a dewar containing a water–glycol-based bath fluid. The bath temperature was measured with a glass thermometer and maintained by using a Julabo F250 recirculating cooler. For measurements at and above 333 K, temperature control was achieved using the heating mantle provided with the 3Flex instrument.

The sources of uncertainties in the experimental method described above are summarized in Table 3. The method was validated using the reference samples and measurement files provided by Micromeritics. CO_2_ adsorption and desorption isotherms for a zeolite 13X reference material at 273 K (Figure S4) and N_2_ adsorption and desorption isotherms for a silica–alumina reference material at 77 K (Figure S5) were measured and compared with the reference data provided by Micromeritics. Independent aliquots of both materials were measured on all three ports of our instrument to confirm the data reproducibility.

**Table 3:** Sources of Uncertainties for the Experimental Measurements of the Equilibrium Gas Sorption Isotherms

parameter	equipment	model	tolerance
pressure (*P*)	pressure transducer, 1000 Torr	TE 85–015A	0.001 · *P*
	pressure transducer, 10 Torr	MKS 626B11T	0.0025 · *P*
	pressure transducer, 1 Torr	MKS 626B.1T	0.005 · *P*
mass	analytical balance	OHAUS Adventurer Pro	0.0001 g
temperature	thermometer	Brannan 44/010/7	1.0 K
	recirculating cooler	Julabo F250	0.1 K

Temperature-dependent Toth (td-Toth), chemi-physisorption (CP), and virial isotherm model fits for the CO_2_ adsorption data, single-site Langmuir (SSL) isotherm model fits for the N_2_ data, and Guggenheim–Anderson–de Boer (GAB) isotherm model fits for the H_2_O adsorption data were performed using the procedure described in our previous works.^42,43^ This procedure was carried out with MATLAB R2020a (The Mathworks Inc.) using the in-house software package isothermFittingTool.^44^

The isosteric heats of adsorption for CO_2_, N_2_, and H_2_O were calculated by using the van’t Hoff equation for specified loading (*n*) and corresponding pressure (*P*) values. The isotherm data (*P* vs *n*) at each temperature (*T*) were first interpolated using smoothing spline interpolation in MATLAB. *m* points with 0.01 mmol g^–1^ intervals were generated over the loading range common to all temperatures, and the corresponding *P* values were determined using the smoothing spline interpolation. A linear regression was then applied to the ln(*P*) versus 1/*T* data for each value of *n* using the LINEST function in Microsoft Excel. The heat of adsorption for each value of *n* (Δ*H*_*n*_) was then calculated by using eq 5, where the derivative corresponds to the slope of the linear regression, and *R* is the universal gas constant.
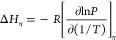
5

The limiting heats of adsorption (Δ*H*_0_) for CO_2_, N_2_, and H_2_O were calculated using the Henry constants determined for each isotherm temperature. Virial plots (*n* vs ln(*P*/*n*)) were created, and the low loading data at each temperature were fitted to a linear equation. The negative exponent of the intercept gives the Henry constant (*K*). A linear regression can then be applied to ln*K* versus 1/*T*, from which the slope can be used to calculate Δ*H*_0_, as shown in eq 6.
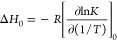
6

## 3 Results and Discussion

### 3.1 Material Properties

#### 3.1.1 Chemical and Textural Properties

**Figure 1:**
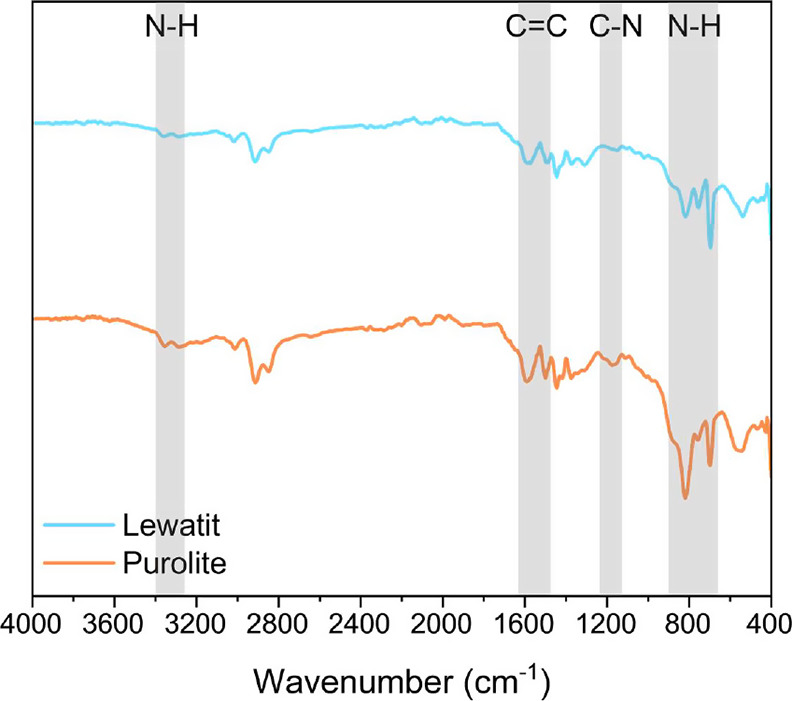
FTIR spectra for dried and ground Lewatit and Purolite resins.

Lewatit and Purolite present as spherical beads with 50–60% water content and particle sizes between 0.3–1.2 mm.^35,36^ Images of the beads before and after drying are shown in Figure S6. Both Lewatit and Purolite are reported by their manufacturers to consist of styrene-divinylbenzene polymer backbones functionalized with primary amines, which should drive the CO_2_ adsorption. We confirmed the presence of these functionalities for both resins using FTIR analysis (Figure 1). The spectra exhibit bands between 1625 and 1500 cm^–1^ corresponding to aromatic C=C bonds, bands between 3400 and 3300 cm^–1^ and 895 and 650 cm^–1^ characteristic of NH_2_ vibrations, and bands around 1200 cm^–1^ characteristic of C–N bonds.^45^ We then used elemental analysis to quantify the nitrogen (N) content in each resin, which is representative of the level of amine functionalization. As seen in Table 4, Purolite has a higher amine content (10.2 wt % of N) than Lewatit (8.3 wt % of N). The N content measured for Lewatit is not far off from the value of 9.5 wt % reported by Yu et al.^46^ Assuming that CO_2_ adsorption only occurs under dry conditions where two N atoms are needed to capture one CO_2_ molecule (see eq 1), the theoretical CO_2_ capacity for Lewatit and Purolite would be 2.96 and 3.64 mmol g^–1^, respectively. We refer to these values later in our study as we quantify the CO_2_ sorption.

**Table 4:** Summary of CHN Elemental Analysis for Lewatit and Purolite Resins, With Uncertainty Bounds for a 95% Confidence Interval

sample	C (%wt)	H (%wt)	N (%wt)
Lewatit	82.5 ± 0.1	8.1 ± 0.1	8.3 ± 0.1
Purolite	79.6 ± 0.4	8.2 ± 0.1	10.2 ± 0.3

**Figure 2:**
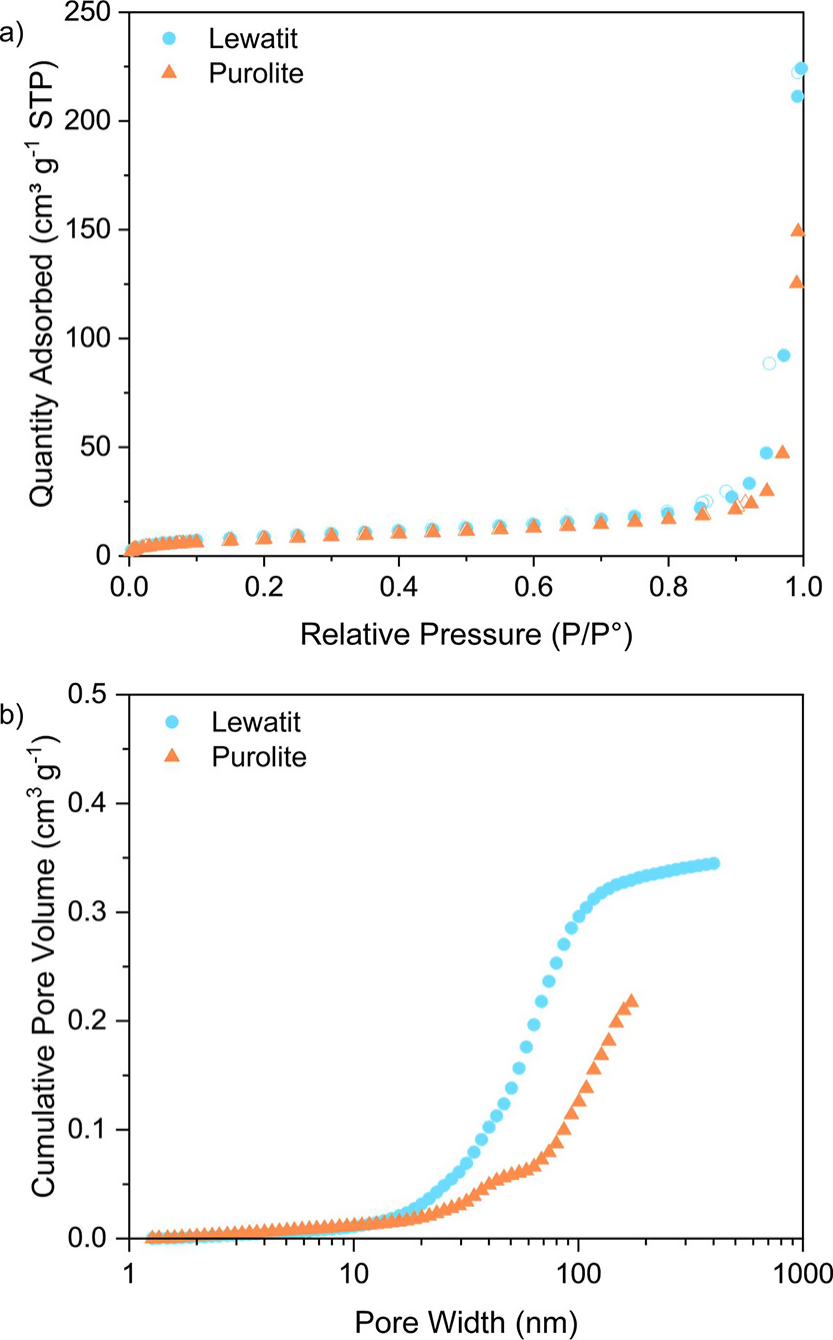
Porosity analysis of Lewatit and Purolite resins. (a) N_2_ sorption isotherms at 77 K (filled symbols = adsorption; open symbols = desorption). (b) Cumulative pore size distribution obtained from N_2_ sorption at 77 K with DFT analysis.

We used N_2_ sorption at 77 K to determine the BET area and pore size distribution for Lewatit and Purolite, which are reported as macroporous resins by the manufacturers. Images of the porous surface of both resins are shown in Figure S7. Both resins have similarly low BET areas of 31 and 28 m^2^ g^–1^, respectively, indicative of macroporous materials. We can also observe from the N_2_ sorption isotherms and pore size distribution the absence of micropores in the materials (Figure 2). The total pore volumes of Lewatit and Purolite, obtained by a direct reading of the N_2_ isotherms at 0.99 *P*/*P*°, are 0.34 and 0.23 cm^3^ g^–1^, respectively. This, however, is an underestimation of the actual total pore volume for these materials as N_2_ sorption at 77 K is ill-suited for macropore analysis. Instead, we estimated the total pore volumes of Lewatit and Purolite to be 0.43 and 0.64 cm^3^ g^–1^, respectively, by determining the bed and skeletal densities of the materials. Using He pycnometry, we found that Purolite and Lewatit exhibit similar skeletal densities (1.15 vs 1.12 g cm^–3^, respectively), but Purolite has a bed density lower than that of Lewatit (0.42 vs 0.48 g cm^–3^, respectively). This result implies that a lower mass of Purolite can fill a specified bed volume compared to that of Lewatit. Purolite also has a higher total pore volume, higher porosity, and lower particle density than Lewatit. The values of bed density, particle density, and porosity are typically needed as inputs for process modeling and are reported in Table 5, along with the total pore volumes and BET areas. Literature values for these properties are provided in Table S1 where available for comparison.

**Table 5:** Summary of Density (Skeletal, Particle, and Bed) and Textural Property (Total Pore Volume, Particle Voidage, and BET Area) Values for Dried Lewatit and Purolite Resins^a^

	Lewatit	Purolite
ρ_skeletal_(g cm^–3^)	1.1231 ± 0.0002	1.1471 ± 0.0002
ρ_particle_(g cm^–3^)	0.744 ± 0.001	0.663 ± 0.002
ρ_bed_(g cm^–3^)	0.476 ± 0.001	0.424 ± 0.002
*V*_total_(cm^3^ g^–1^)	0.454 ± 0.001	0.637 ± 0.001
ε_particle_	0.338 ± 0.001	0.422 ± 0.001
*S*_BET_(m^2^ g^–1^)	31	28

a Uncertainty bounds for a 95% confidence interval are provided when available.

#### 3.1.2 Thermal Properties

**Figure 3:**
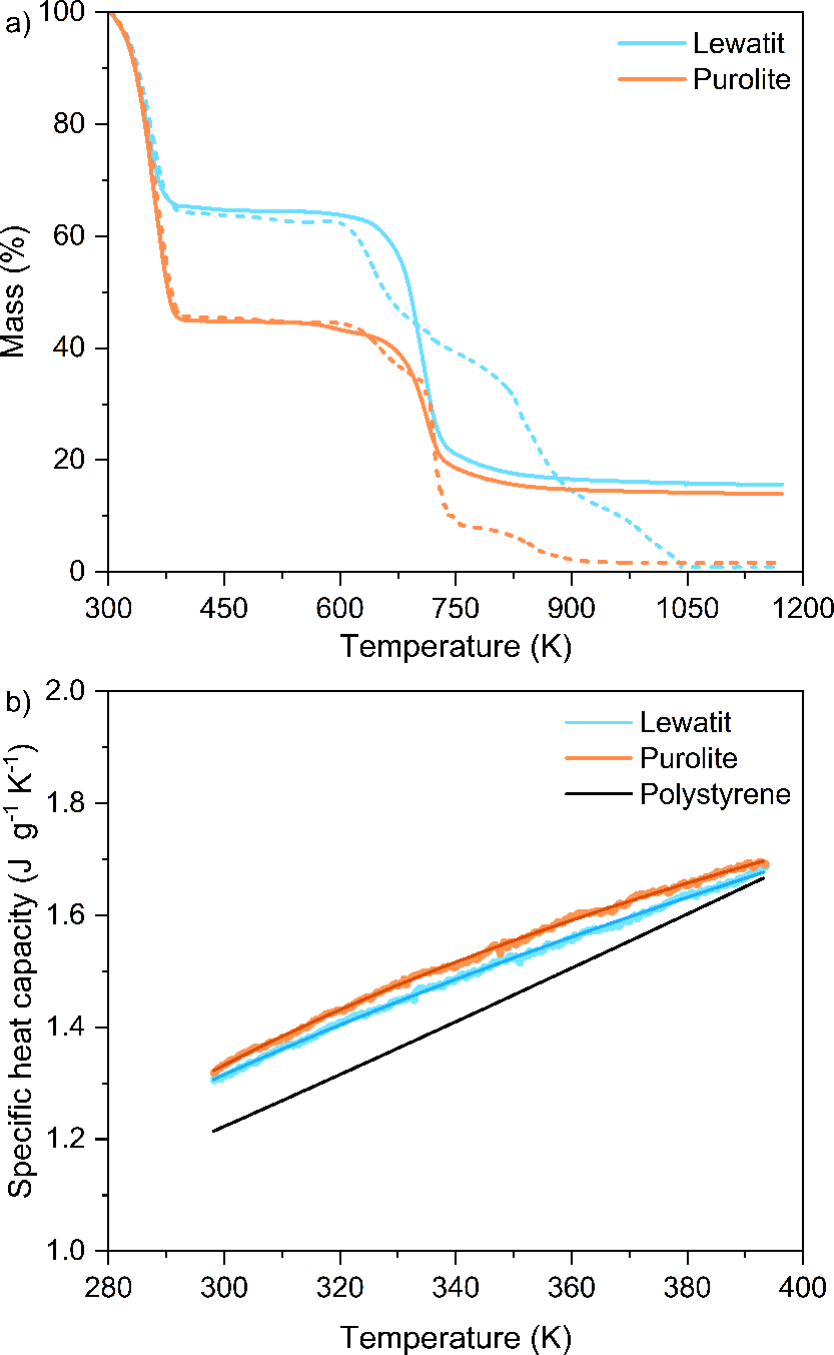
Thermal analysis of the Lewatit and Purolite resins. (a) TG curves under N_2_ (solid lines) and air (dotted lines) atmospheres up to 1173 K. (b) Specific heat capacities of Lewatit and Purolite measured as a function of temperature from 298 to 393 K compared to the reported values of polystyrene, where the darker solid lines represent the fitted results using eq 7.

We used thermogravimetric analysis (TGA) to determine the thermal stability of the resins, which dictates the maximum temperature that can be used for desorption in a DAC process without degrading the material. The manufacturer of Lewatit recommends a maximum operating temperature of 373 K,^35^ while none is recommended for Purolite. As seen in Figure 3a, both resins contain adsorbed water (weight loss <373 K), with Purolite having approximately 20% higher water content than Lewatit. Small reductions in mass are observed for both Lewatit and Purolite, starting at 398 K under both N_2_ and air atmospheres (see Figure S8), likely associated with amine degradation.^47^ The larger decreases in mass between 573 to 673 K for both materials under both conditions correspond to the degradation of the polymer backbone.

**Table 6:** Fitting Parameters for the Specific Heat Capacities of Lewatit and Purolite

parameter	unit	Lewatit	Purolite
*a*	J K g^–1^	–3.23 × 10^4^	–5.86 × 10^4^
*b*	J g^–1^ K^–2^	0.00227	0.000994
*c*	J g^–1^ K^–1^	–0.994	–1.69

We used differential scanning calorimetry (DSC) to determine the specific heat capacities of the resins in the temperature range 298–393 K, which is a possible range of temperatures that the materials might experience in a DAC process. The heat capacity is a necessary input in process modeling as it relates to the energy input needed to heat an adsorbent during the desorption process, where an adsorbent with a higher heat capacity would require more energy to be heated to a set temperature. As seen in Figure 3b, both Lewatit and Purolite have similar heat capacities, as expected due to their similar chemical structures. Both values are similar to the heat capacity of polystyrene in the same temperature range (1.2–1.7 J g^–1^ K^–1^),^48^ which forms the backbone of both resins, and agree well with previously measured specific heat capacity values for Lewatit of 1.6 J g^–1^ K^–1^.^49^ Our experimental data can be modeled using eq 7 shown below:^48^
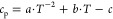
7where *c*_p_ is the specific heat capacity, *T* is the temperature, and *a*, *b*, and *c* are constants to be fitted. The fitted parameters for Lewatit and Purolite are listed in Table 6.

### 3.2 Sorption Properties

#### 3.2.1 CO_2_ Sorption

**Figure 4:**
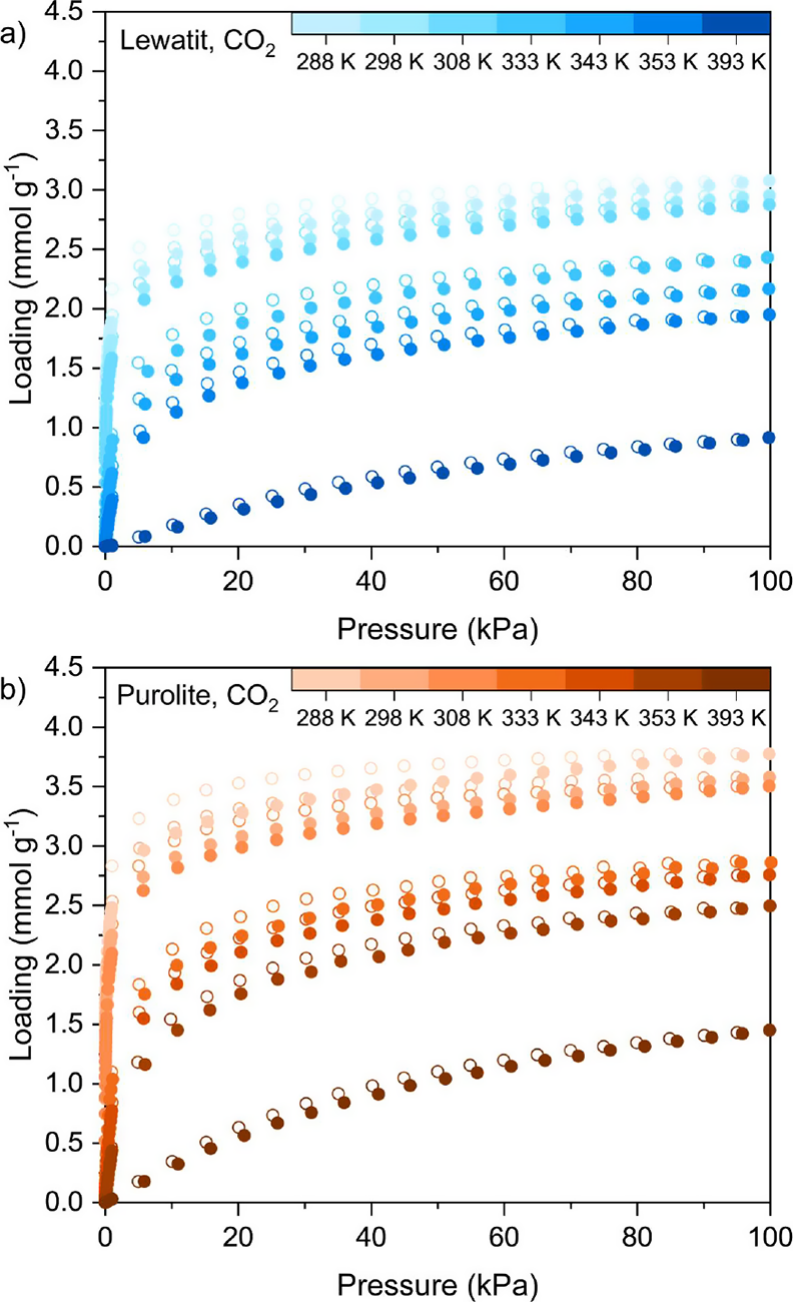
Equilibrium adsorption (filled symbols) and desorption (hollow symbols) isotherms for CO_2_ measured at 288, 298, 308, 333, 343, 353, and 393 K up to 100 kPa for (a) Lewatit and (b) Purolite.

We measured the equilibrium CO_2_ adsorption and desorption isotherms up to 100 kPa for Lewatit and Purolite at 288, 298, 308, 333, 343, 353, and 393 K (Figure 4), with more than 20 data points measured below 1 kPa at most temperatures. The adsorption data points for each temperature for Lewatit and Purolite are provided in Table A.1, while all adsorption and desorption data are provided as AIF files^50^ and CSV files in the Supporting Information. We ensured that equilibrium was reached by repeating the adsorption and desorption measurements at 308 K, with the equilibrium interval increased from 60 to 90 s, and observed no change in the results (Figure S9).

The results demonstrate that Purolite adsorbs more CO_2_ than Lewatit at 0.04 kPa CO_2_ pressure, which is representative of the CO_2_ feed stream for a DAC process. At an adsorption temperature of 298 K and in the presence of 0.04 kPa CO_2_, the gravimetric CO_2_ adsorption capacity for Purolite versus Lewatit is approximately 1.3 mmol g^–1^ versus 0.95 mmol g^–1^. Considering the adsorbents’ bed densities (see Table 5), Purolite also has a higher volumetric CO_2_ adsorption capacity of 0.55 mmol cm^–3^_bed_ compared to that of Lewatit (0.45 mmol cm^–3^_bed_). This trend correlates with the higher N content observed for Purolite compared to Lewatit. CO_2_ isotherms for Purolite have not yet been reported in the literature, while CO_2_ isotherms for Lewatit have been measured before.^9,25,51,52^ We compared our adsorption data obtained at 298 K with that obtained by Young et al.^9^ in Figure S10a. The data align very well at low CO_2_ pressures, but the CO_2_ loading reported by Young et al. at pressures closer to 100 kPa is higher than that of our experimental data. This deviation could be due to the different sorption measurement techniques used, i.e., gravimetrically (Young et al.) versus volumetrically (our study).

Hysteresis is observed for both Lewatit and Purolite in Figure 4. In both cases, the extent of hysteresis decreases as the temperature increases, which is indicative of the chemisorbent nature of the materials. At lower temperatures, there is insufficient energy available to remove the chemisorbed CO_2_ at a given pressure, while at higher temperatures (e.g., 393 K), the process is reversible. We attribute this behavior to a true equilibrium effect rather than a kinetic effect. Indeed, we repeated the measurements with a longer equilibration interval (Figure S9) and did not observe any change in the degree of hysteresis in the isotherms.

We also measured adsorption and desorption isotherms at 308 K for both materials approximately a year after our original measurements on the same batch of resins to test if there was any degradation of the adsorbents. As seen from Figure S11, there is no discernible difference between the two measurements for Lewatit; however, there is a slight decrease in loading in the later Purolite measurement compared to the original, which could be attributed to minor degradation of the material during storage. A pilot-scale study by van Paasen et al.^53^ using Lewatit for postcombustion CO_2_ capture showed that approximately 10% of the adsorbent degradation after 33 days was due to shelf-life instability, while the major contributor was oxidative degradation at high operating temperatures during regeneration. These and other sources of oxidative degradation during a DAC process cycle were recently described by Carneiro et al.^54^

**Figure 5:**
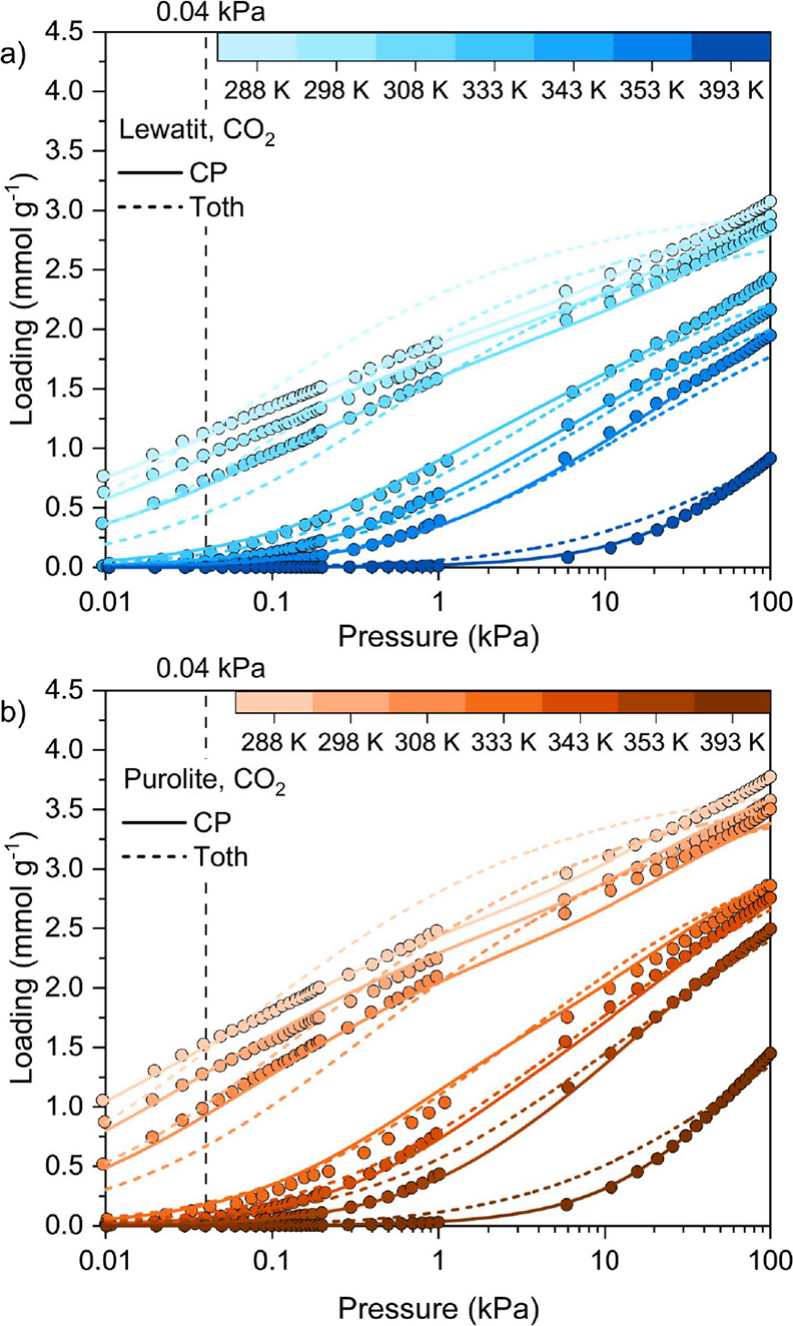
Equilibrium adsorption isotherms for CO_2_ measured at 288, 298, 308, 333, 343, 353, and 393 K up to 100 kPa for (a) Lewatit and (b) Purolite with a log-scale of pressure. Solid lines represent the fitting results from the CP isotherm model, while dashed lines represent the fitting results from the td-Toth isotherm model. The fitting parameters of the isotherms are found in Table 7.

Isotherm model equations that represent the experimental data well are needed to implement the data in process modeling. All previous studies with Lewatit have used the temperature-dependent Toth (td-Toth) isotherm model to describe the loading *n* of CO_2_, given by eqs 8–11:
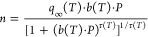
8
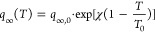
9
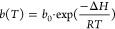
10
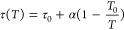
11where *P* is the pressure, *T* is the temperature, *q*_∞_*(T)* is the maximum CO_2_ capacity, *q*_∞,0_ is the maximum CO_2_ capacity at the reference temperature *T*_0_, *b*(*T*) is the CO_2_ adsorption coefficient defined by a constant *b*_0_ and the heat of adsorption Δ*H*, *R* is the universal gas constant, τ(*T*) is the surface heterogeneity parameter, τ_0_ is the surface heterogeneity parameter at *T*_0_*,* and χ and α are the factors determining the temperature dependencies of *q*_∞_(*T*) and τ(*T*). As seen in Figure 5, this model does not fit our measured data very well for either Lewatit or Purolite at the lower temperatures of 288, 298, and 308 K. The fitted parameters are found in Table 7, while the upper and lower bounds imposed on each parameter during the fitting are summarized in Table S2. The fitting parameters determined by Young et al.^9^ (Table S3) are also unable to represent our data well, as shown in Figure S10b. This may be due to varying factors such as a different temperature range and a greater number of data points, particularly in the low-pressure region below 1 kPa.

**Table 7:** Fitting Parameters With Uncertainty Bounds for a 95% Confidence Interval for the td-Toth and CP Isotherm Models for CO_2_ Adsorption for Lewatit and Purolite

equation	parameter	unit	Lewatit	Purolite
td-Toth	*q_∞_*_,0_	mmol g^–1^	3.00 ± 0.05	3.74 ± 0.05
	*b*_0_	(× 10^–11^) kPa^–1^	2.0 ± 0.2	3.4 ± 0.3
	–Δ*H*	kJ mol^–1^	70.1 ± 0.3	70.1 ± 0.2
	*T*_0_	K	298.15	298.15
	τ_0_		0.431 ± 0.009	0.401 ± 0.006
	α		1.4 × 10^–10^ ± 1.1 × 10^–2^	1.43 × 10^–4^ ± 5.8 × 10^–2^
	χ		0.7 ± 0.2	0.3 ± 0.1
CP	*q_∞_*_,0_	mmol g^–1^	2.56 ± 0.01	2.91 ± 0.02
	*b*_0_	(× 10^–16^) kPa^–1^	1.05 ± 0.03	0.96 ± 0.05
	–Δ*H*	kJ mol^–1^	105.51 ± 0.09	105.2 ± 0.1
	*T*_0_	K	298.15	298.15
	τ_0_		0.343 ± 0.02	0.401 ± 0.004
	α		1.39 ± 0.04	1.99 ± 0.10
	χ		1.63 ± 0.05	2.52 ± 0.09
	*q*_∞,c_	mmol g^–1^	1.05 ± 0.03	3.56 ± 0.09
	*b*_0,c_	(× 10^–4^) kPa^–1^	1.03 ± 0.05	21 ± 1
	–Δ*H*_*c*_	kJ mol^–1^	13.5 ± 0.1	7.0 ± 0.2
	*E*_a_	kJ mol^–1^	0.60 ± 0.06	3.13 ± 0.07

Given that td-Toth does not represent our data well, we considered other approaches. While the dominant mechanism in amine-functionalized adsorbents is known to be chemisorption via the reaction shown in eq 1, previous works have also observed a non-negligible contribution from physisorption on the adsorbent surface, which would be more prevalent at lower temperatures.^55,56^ This seems to also apply to Lewatit and Purolite, as their sorption isotherms do not plateau in our measured pressure range. As such, an isotherm model which accounts for both physisorption and chemisorption was previously proposed by Serna-Guerrero et al. to describe CO_2_ adsorption on triamine-grafted pore-expanded MCM-41 from 0.1 to 2000 kPa^55^ and used by Elfving et al. for CO_2_ adsorption on a proprietary amine-functionalized resin from 0.01 to 0.5 kPa for DAC applications.^32^ This model is given by the following equation:
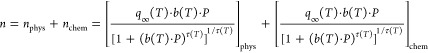
12where *q*_∞_(*T*), *b*(*T*), and τ(*T*) are given by eqs 9–11. However, to implement this model, the contributions from *n*_phys_ and *n*_chem_ must be separately determined experimentally. Serna-Guerrero et al. did so by measuring adsorption isotherms for ungrafted MCM-41 and assumed that this was the contribution for *n*_phys_, while Elfving et al. used a N_2_ purge with and without heat to separately desorb CO_2_ captured by chemisorption versus physisorption, respectively. Other studies such as those by Pai et al.,^57^ Hefti et al.,^58^ and Hughes et al.^59^ have also proposed solutions for modeling isotherms with both chemisorption and physisorption contributions, either by using a weighted isotherm model or modeling the chemisorption and physisorption separately, though the application here was for S-shaped isotherms from diamine-appended MOFs. For our purposes, we propose a modification of eq 12 as follows, which we refer to here as the chemi-physisorption (CP) isotherm model.
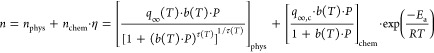
13where *q*_∞,c_ is the maximum CO_2_ capacity associated with chemisorption, *E*_a_ is the activation energy, and again *q*_∞_(*T*), *b*(*T*), and τ(*T*) are given by eqs 9–11. Here, the contribution from physisorption is given by the td-Toth isotherm model, while the contribution from chemisorption is given by the single-site Langmuir (SSL) model. We assume that the transition between physisorption and chemisorption follows an Arrhenius behavior given by η (i.e., at a given temperature, some fraction of molecules will have sufficient activation energy to participate in chemisorption). Thus, the model favors physisorption at lower temperatures and increases the contribution of chemisorption as the temperature increases, while still allowing for the exothermic nature of adsorption at even higher temperatures. As shown in Figure 5, the model provides an improved fit of the experimental data. We performed an F-test between the CP and td-Toth isotherm model fitting results and confirmed that the improved results were indeed significant and not due to additional fitting parameters (ps S16–S17). The fitted parameters can be found in Table 7, while the upper and lower bounds imposed on each parameter during the fitting are summarized in Table S2.

As the above CP isotherm model has not yet been thoroughly validated, we also fitted our experimental data to the well-known virial equation as an alternative (Figure S12), given by
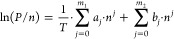
14where *a* and *b* are the characteristic virial coefficients, *n* is the loading, *P* is the pressure, *T* is the temperature, *m*_1_ = 3, and *m*_2_ = 1. The fitted parameters can be found in Table S4, and the upper and lower bounds imposed on each parameter during the fitting are summarized in Table S2. The virial equation provides a reasonable fit of the data; however, it is unable to account for competitive adsorption when used in process modeling and is often difficult to implement due to the need for the equation to be solved numerically as it returns pressure as a function of loading, rather than the conventional loading as a function of pressure. As a result, the CP isotherm model we propose is likely the most appropriate method to use to represent our measured data.

#### 3.2.2 N_2_, Ar, and O_2_ Sorption

**Figure 6:**
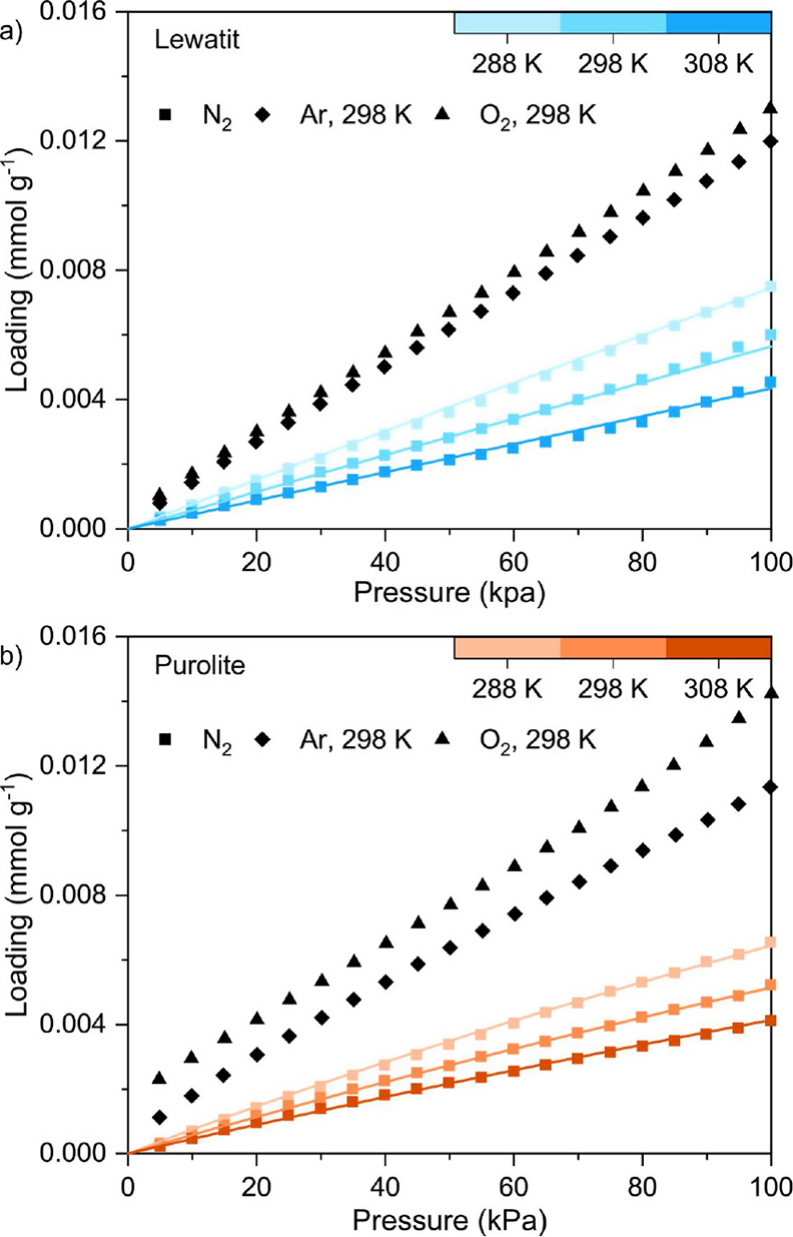
Equilibrium adsorption isotherms for N_2_ (squares) measured at 288, 298, and 308 K and for Ar (diamonds) and O_2_ (triangles) at 298 K up to 100 kPa for (a) Lewatit and (b) Purolite. Solid lines represent the SSL isotherm fitting results (fitting parameters can be found in Table 8).

We measured N_2_ sorption isotherms up to 100 kPa for Lewatit and Purolite at 288, 298, and 308 K (Figure 6, desorption shown in Figure S13a,b), as well as Ar and O_2_ isotherms up to 100 kPa at 298 K (Figure 6, desorption shown in Figure S13c,d). These are all gases present in air at concentrations higher than CO_2_. They may be competitive for CO_2_ adsorption sites or adsorb on the backbone and impact the achievable product purity. The adsorption data points for each adsorbate and temperature for Lewatit and Purolite are provided in Table A.2, while all experimental adsorption and desorption data are provided as AIF files^50^ and CSV files in the Supporting Information. The desorption data shown in Figure S13 exhibit an irregular pattern which we attribute to the low loading levels, which are on the same order of magnitude as the error of the porosity analyzer.

Compared with CO_2_ adsorption, the N_2_, Ar, and O_2_ adsorption levels are almost negligible. The SSL isotherm model shown in eq 15 can be used to represent the N_2_ isotherm experimental data, and the fitting parameters are reported in Table 8
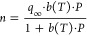
15where *q*_∞_ is the maximum CO_2_ capacity, *b*(*T*) is given by equation 10, *n* is the loading, *P* is the pressure, and *T* is the temperature. The upper and lower bounds imposed on each parameter during the fitting are summarized in Table S2. Interestingly, both Lewatit and Purolite are selective to O_2_ compared to N_2_, where many traditional adsorbents such as zeolites have the opposite performance. Although their O_2_ adsorption capacities are low, these materials may have potential applications in air separation where it would be more energy- and cost-effective to adsorb O_2_ (21%_vol_ in air) versus N_2_ (78%_vol_ in air). To the best of our knowledge, O_2_ isotherms have not been previously measured for Lewatit, and so this characteristic was not observed. In addition, the O_2_ isotherm on Purolite at 298 K does not appear to intersect with the origin. This may be an indication for the degradation of, or reaction with, the amine functionalization of the sorbent. It also aligns with the impact of oxidative degradation of the amines on the CO_2_ capacity, as discussed in Section 3.2.1.

**Table 8:** Fitting Parameters With Uncertainty Bounds for a 95% Confidence Interval for the SSL Isotherm Model for N_2_ Adsorption for Lewatit and Purolite

equation	parameter	unit	Lewatit	Purolite
SSL	*q*_*∞*_	mmol g^–1^	0.315 ± 0.004	0.0419 ± 0.0002
	*b*_0_	(×10^–8^) kPa^–1^	5.06 ± 0.06	75.6 ± 0.05
	–Δ*H*	kJ mol^–1^	20.30 ± 0.03	18.65 ± 0.02

**Figure 7:**
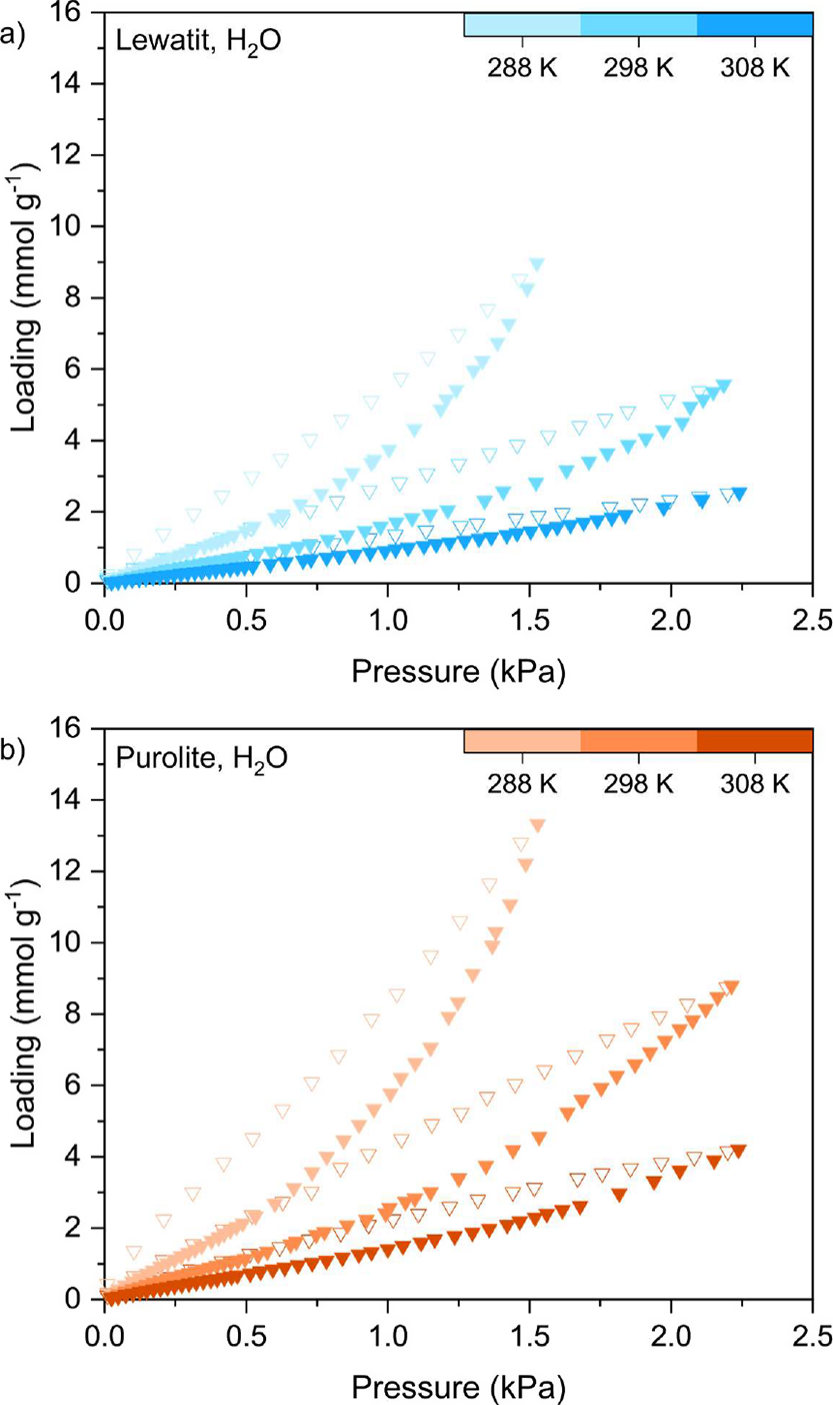
Equilibrium adsorption (filled symbols) and desorption (hollow symbols) isotherms for H_2_O measured at 288 K up to 1.6 kPa and 298 and 308 K up to 2.3 kPa for (a) Lewatit and (b) Purolite.

#### 3.2.3 H_2_O Sorption

We measured the H_2_O adsorption and desorption isotherms for Lewatit and Purolite at 288 K up to 1.6 kPa and at 298 and 308 K up to 2.3 kPa (Figure 7). The adsorption data points for each temperature for Lewatit and Purolite are provided in Table A.3, while all experimental adsorption and desorption data are provided as AIF files^50^ and CSV files in the Supporting Information. Water adsorption behavior is particularly important to understand for amine-functionalized adsorbents as the presence of H_2_O from the relative humidity in the air is known to increase the CO_2_ uptake of these materials as well as minimize amine degradation.^11,60,61^ While directly measuring CO_2_–H_2_O coadsorption isotherms is ideal, it remains experimentally challenging; for this study, we chose to only measure unary water sorption isotherms.

The water adsorption behavior of both resins follows a Type III isotherm behavior according to IUPAC classification,^38^ which represents unrestricted multilayer adsorption on macroporous materials. When the loading data are plotted against the adsorption potential, ϵ, given by
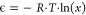
16where *R* is the universal gas constant, *T* is the temperature, and *x* is the relative pressure, the results collapse on to a characteristic curve typical of nonmicroporous adsorbents (Figure S14).^62^

**Figure 8:**
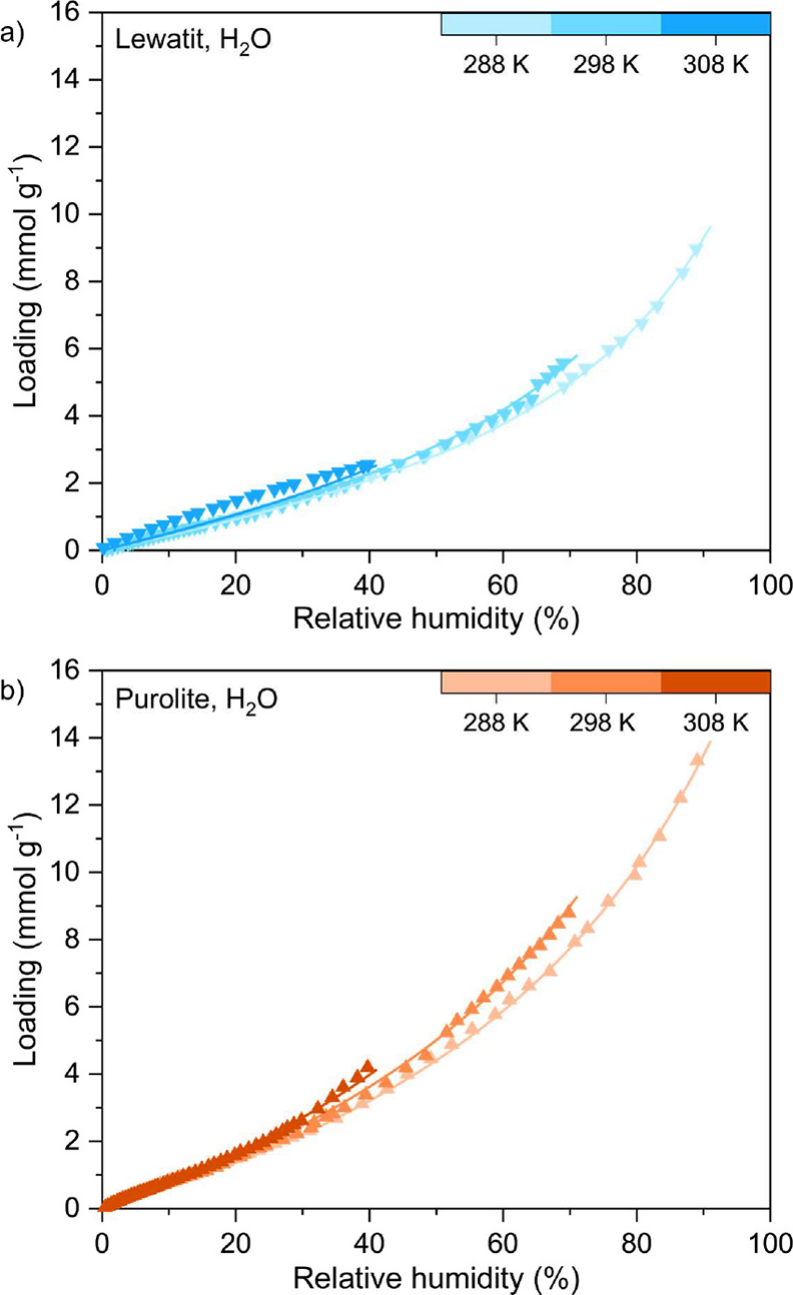
Equilibrium adsorption isotherms for H_2_O measured at 288, 298, and 308 K as a function of relative humidity for (a) Lewatit and (b) Purolite. Solid lines represent the GAB isotherm fitting results (fitting parameters are listed in Table 9).

We used the Guggenheim–Anderson–de Boer (GAB) model to fit the experimental data as a function of relative pressure for both materials, as used by Young et al. for Lewatit.^9^ The model is as follows:
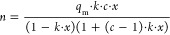
17
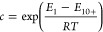
18
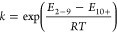
19
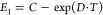
20
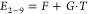
21
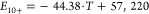
22where *n* is the water loading on the resins, *x* is the relative pressure, *q*_m_ is the water loading of the monolayer, and *k* and *c* are affinity parameters governed by the heats of adsorption of the first (*E*_1_), second to ninth (*E*_2–9_), and tenth and higher (*E*_10+_) layers of adsorbed water. *E*_10+_ is also equivalent to the heat of condensation of water, and *C*, *D*, *F*, and *G* are constants to be fitted. The fitting results are shown in Figure 8, and the fitting parameters are reported in Table 9, while the upper and lower bounds imposed on each parameter during the fitting are summarized in Table S2. A comparison of our adsorption data obtained at 298 K with that obtained by Young et al.^9^ is shown in Figure S15a, and we observe good agreement between the two data sets. An overlay of their fitted isotherm parameters (Table S5) with our experimental data is also shown in Figure S15b. Their parameters reasonably fit our measured 308 K data but deviate from our data at 288 and 298 K. This could be because Young et al. fitted their parameters to experimental data collected at a wider and higher temperature range, from 298 to 373 K.

**Table 9:** Fitting Parameters With Uncertainty Bounds for a 95% Confidence Interval for the GAB Isotherm Model for H_2_O Adsorption for Lewatit and Purolite

equation	parameter	unit	Lewatit	Purolite
GAB	*q*_m_	mmol g^–1^	3.23 ± 0.02	7.75 ± 0.07
	*C*	kJ mol^–1^	46.83 ± 0.04	45.79 ± 0.04
	*D*	K^–1^	0.02446 ± 0.00008	0.02441 ± 0.00008
	*F*	kJ mol^–1^	53.31 ± 0.01	52.52 ± 0.01
	*G*	kJ mol^–1^ K^–1^	–0.03268 ± 0.00003	–0.03108 ± 0.00004

#### 3.2.4 Isosteric Heats of Adsorption

**Figure 9:**
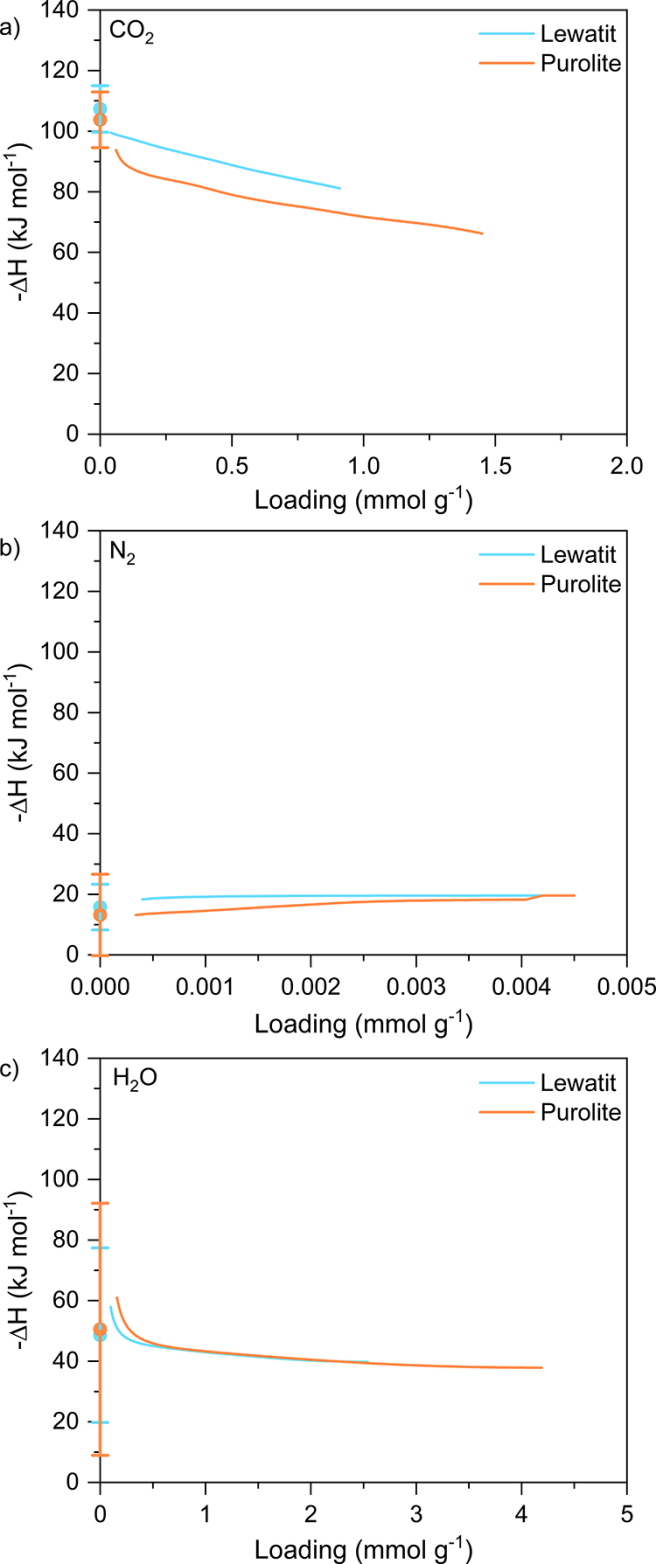
Isosteric heats of adsorption of Lewatit and Purolite for (a) CO_2_, (b) N_2_, and (c) H_2_O.

We calculated the limiting heat of adsorption (Table 10), as well as the heat of adsorption as a function of loading (i.e., isosteric heat of adsorption), of CO_2_, N_2_, and H_2_O for both Lewatit and Purolite (Figure 9). The intermediate results obtained during the calculation of the limiting heats of adsorption, as described in Section 2.5, are presented in Figures S16–S21. Confidence intervals associated with the heat of adsorption as a function of loading calculations are presented in Figures S22 and S23, along with the additional data calculated using alternative isotherm temperatures for CO_2_. The smoothing parameter values applied for each adsorbent–adsorbate combination, as described in Section 2.5, are presented in Table S6. The heats of adsorption serve as inputs in process models and link to the energy requirements for desorption in a DAC process, where a higher heat of adsorption implies a higher energy needed to regenerate the adsorbent. Both materials have high CO_2_ heats of adsorption characteristic of chemisorbents. At the loading levels corresponding to 0.04 kPa_a_ CO_2_, Lewatit and Purolite have heats of adsorption of ∼81 and 69 kJ mol^–1^, respectively. The data presented in Figure 9a were obtained using the 333, 343, 353, and 393 K CO_2_ isotherms. The isosteric heats of adsorption calculated using the lower temperatures of 288, 298, and 308 K are shown in Figure S22 and show an eventual transition to the physisorption range, commonly said to be below 50 kJ mol^–1^.

**Table 10:** Limiting Heats of Adsorption of CO_2_, N_2_, and H_2_O for Lewatit and Purolite with Uncertainty Bounds for a 95% Confidence Interval

	Lewatit	Purolite
–Δ*H*_0_, CO_2_ (kJ mol^–1^)	107 ± 8	104 ± 9
–Δ*H*_0_, N_2_ (kJ mol^–1^)	16 ± 8	13 ± 13
–Δ*H*_0_, H_2_O (kJ mol^–1^)	49 ± 29	50 ± 42

Also characteristic of many chemisorbents, both materials exhibit low N_2_ heats of adsorption of ∼20 kJ mol^–1^. The N_2_ heat of adsorption stays relatively constant as a function of loading, indicating homogeneous coverage of the adsorbent surface.

For H_2_O, the heat of adsorption steeply decreases from 60 kJ mol^–1^ between 0 and 0.5 mmol g^–1^ loading, to a plateau of approximately 40 kJ mol^–1^, which is close to the heat of condensation of water of 44.0 kJ mol^–1^ at 298 K given by eq 22 and corroborated by NIST.^63^ This trend possibly reflects two different sorption mechanisms consisting of a monolayer of resin–H_2_O interactions, followed by multilayers of H_2_O–H_2_O interaction.

## 4 Conclusions

In this study, we endeavored to experimentally determine as many of the material and sorption properties needed for process-scale evaluation of DAC adsorbents as possible for Purolite A110, a commercially available amine-functionalized polymeric resin, and compared its properties to those of Lewatit VP OC 1065, a current benchmark adsorbent for DAC. Lewatit and Purolite have similar chemical features, specific heat capacities, thermal stability, and skeletal density. Purolite has lower bed and particle densities than Lewatit and a higher total pore volume and porosity. In terms of its gas sorption performance, Purolite has a higher gravimetric and volumetric CO_2_ adsorption capacity than Lewatit, attributed to its higher N content, as well as higher H_2_O adsorption, while both materials have low and comparable adsorption of N_2_, Ar, and O_2_. Purolite has a lower CO_2_ heat of adsorption compared to Lewatit and a similar H_2_O heat of adsorption. This trend suggests potentially lower energy requirements for desorption and thus lower process operating costs, but this will ultimately depend on actual loadings. In our work, we also proposed an empirical CO_2_ sorption isotherm model combining chemisorption and physisorption terms. The model fitted the data well and could be used for the numerical expression of the sorption isotherms. Based on the data we have collected, we propose that Purolite A110 is indeed a suitable adsorbent for DAC. With this work, we hope we have provided the adsorption community with the necessary information to conduct process-scale modeling and optimization to assess the process performance of Purolite for adsorption-based DAC and continue to advance the development and scale-up of this technology.

## Supporting Information Available

Additional information on bed density, specific heat capacity, and thermal stability measurements; validation of the porosity analyzer using reference materials; images of Lewatit and Purolite resin beads; literature value comparisons of textural properties, CO_2_ adsorption, and H_2_O adsorption for Lewatit; checks for sample stability and achievement of equilibrium; bounds for isotherm fitting parameters; F-test procedure; fitting of the Virial equation to CO_2_ isotherms; N_2_, Ar, and O_2_ desorption isotherm data; H_2_O adsorption potentials; and additional information on calculations of limiting and isosteric heats of adsorption (PDF)
Isotherm data of Lewatit (ZIP)
Isotherm data of Purolite (ZIP)
Isotherm data in tabular format of Lewatit and Purolite resin beads for CO_2_, H_2_O, N_2_, Ar and O_2_ (PDF)

## Notes

The current version of the software package used for isotherm fitting and uncertainty calculation is available on the Imperial College London Github repository and can be accessed at https://github.com/ImperialCollegeLondon/IsothermFittingTool. The version of the code used in this publication is referred to by the git commit ID “060ff65”.
