# Supplementary material for: Measurement of Physicochemical Properties and CO2, N2, Ar, O2, and H2O Unary Adsorption Isotherms of Purolite A110 and Lewatit VP OC 1065 for Application in Direct Air Capture
Source: J Chem Eng Data. 2023 Oct 11;68(12):3499–511. doi: 10.1021/acs.jced.3c00401 (PMC10726313; doi:10.1021/acs.jced.3c00401)
Supplement: Supplementary file 1 — je3c00401_si_001.pdf [file je3c00401_si_001.pdf]

Measurement of physicochemical properties and CO_2, N_2, Ar, O_2, and H_2O unary adsorption isotherms of Purolite A110 and Lewatit VP OC 1065 for application in direct air capture

Supporting Information

May-Yin (Ashlyn) Low,[1] David Danaci,[1] Hassan Azzan[1], Robert T. Woodward[2] Camille Petit[1]*

[1] Barrer Centre, Department of Chemical Engineering, Imperial College London, London SW7 2AZ, United Kingdom

[2] Institute of Materials Chemistry & Research, University of Vienna, 1090 Vienna, Austria

*Corresponding author: camille.petit@imperial.ac.uk

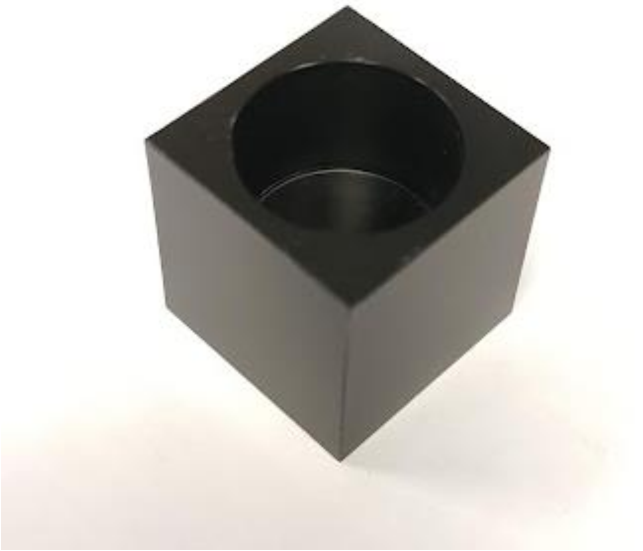

Figure S1. 3D-printed container for measuring the bed densities of Lewatit and Purolite. The outer cube dimensions are 2.0 × 2.0 × 2.0 cm, while the cylindrical inset measures 1.6 cm in dimeter and 1.2 cm in height.

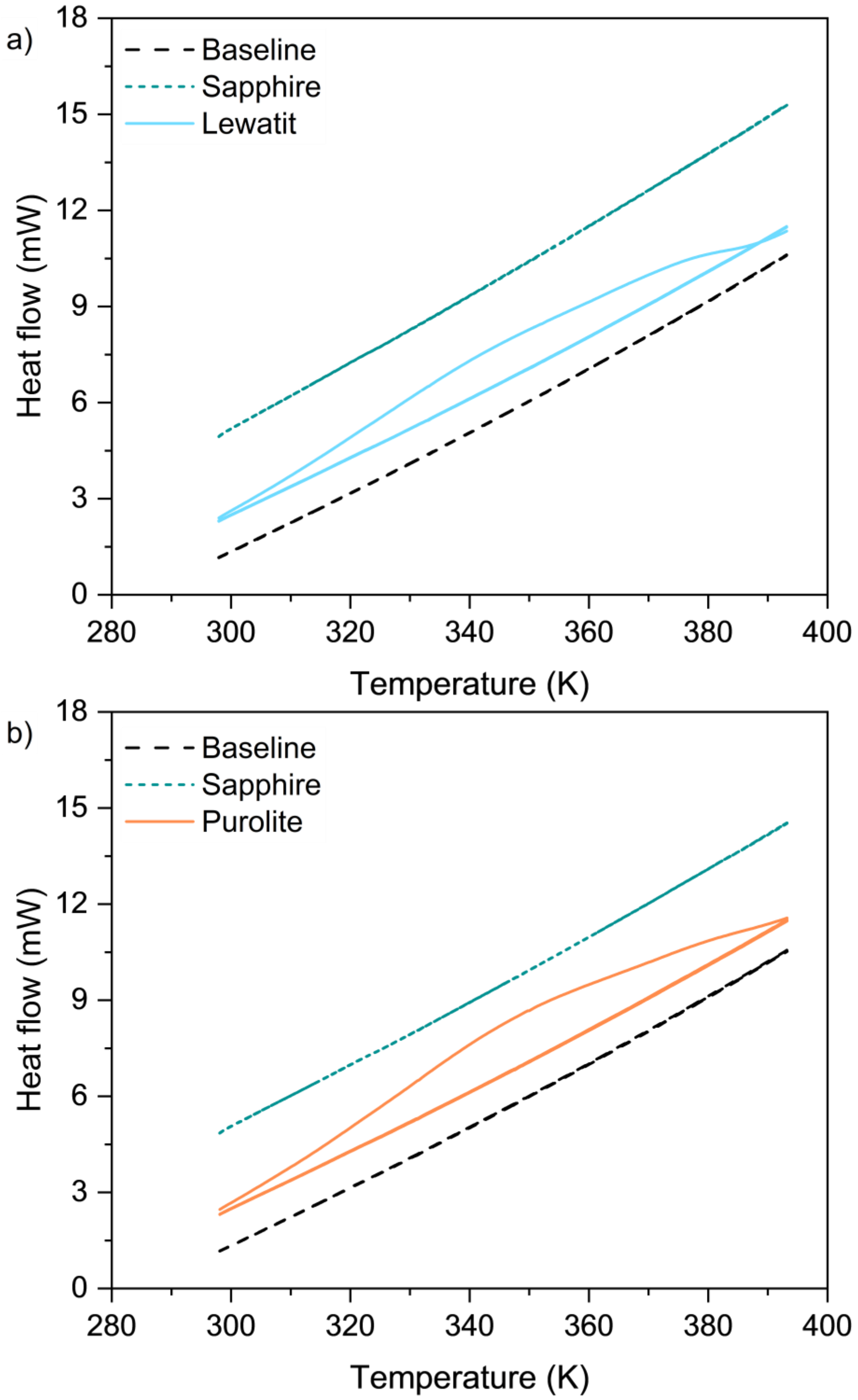

Figure S2. Heat flow curves of 3 baseline runs, 3 sapphire runs, and 4 sample runs for a) Lewatit and b) Purolite heat capacity measurements. For both samples, the first heat flow curve differs from the following three.

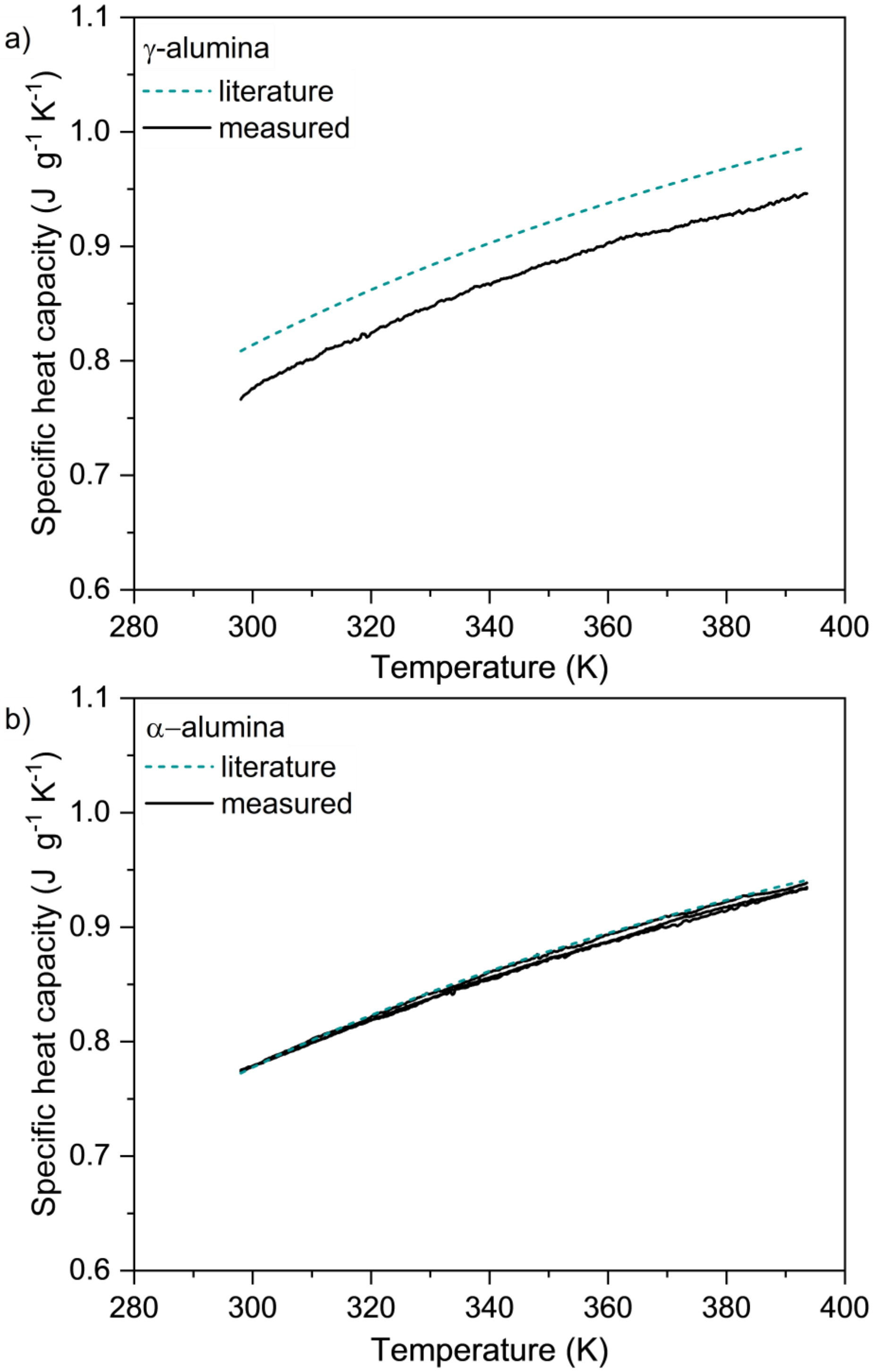

Figure S3. Heat capacities of measured a) γ-alumina powder and b) α-alumina disc provided by Perkin-Elmer, in comparison to literature values provided by NIST [1].

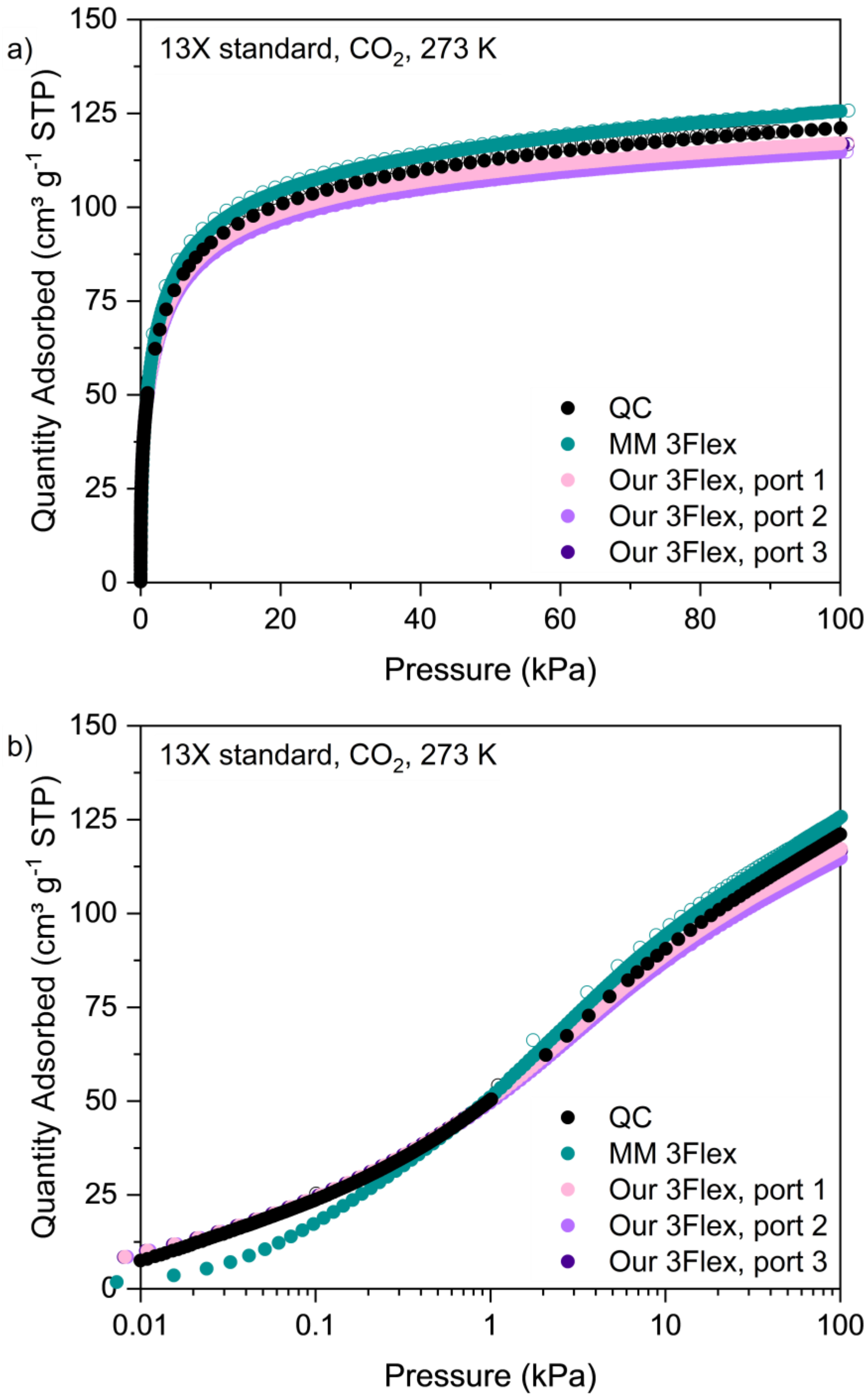

Figure S4. Comparison of equilibrium adsorption (filled symbols) and desorption (hollow symbols) isotherms of CO_2 for a zeolite 13X reference material measured at 273 K by both Micromeritics (MM) on a 3Flex porosity analyser, and by us on Quantachrome Autosorb iQ3 (QC) and Micromeritics 3Flex porosity analysers. Samples were degassed ex-situ at 0.002 kPa for 1 h at 363 K then at 623 K for at least 8 h. Samples were then degassed in-situ at 573 K and 0.000002 kPa for 4 h. Isotherms are shown in a) linear scale and b) log scale of pressure.

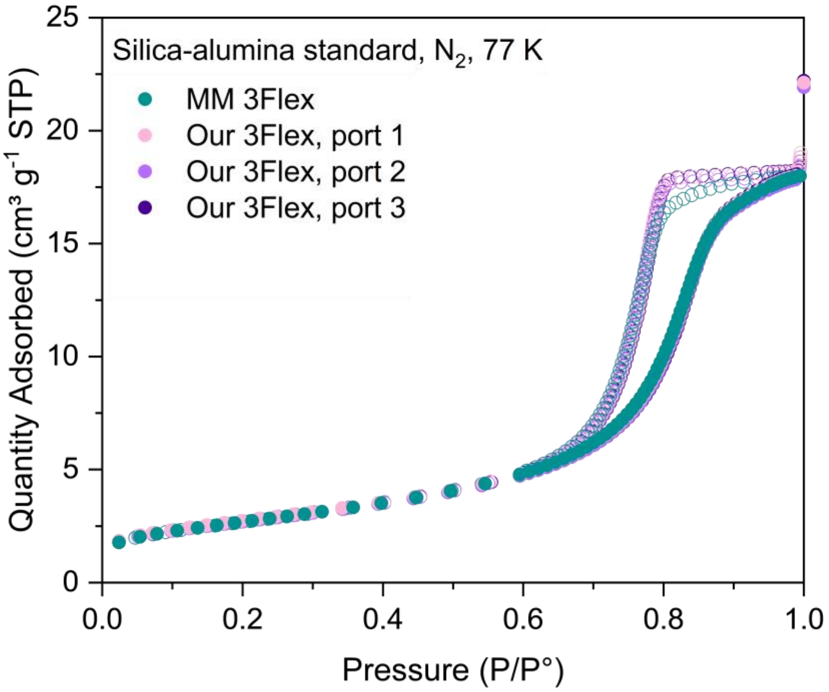

Figure S5. Comparison of equilibrium adsorption (filled symbols) and desorption (hollow symbols) isotherms of N_2 measured at 77 K for a silica-alumina reference material run by both Micromeritics (MM) and ourselves on 3Flex porosity analysers. Samples were degassed ex-situ at 0.002 kPa for 1 h at 363 K then at 623 K for at least 4 h. Samples were then degassed in-situ at 623 K and 0.000002 kPa for 4 h.

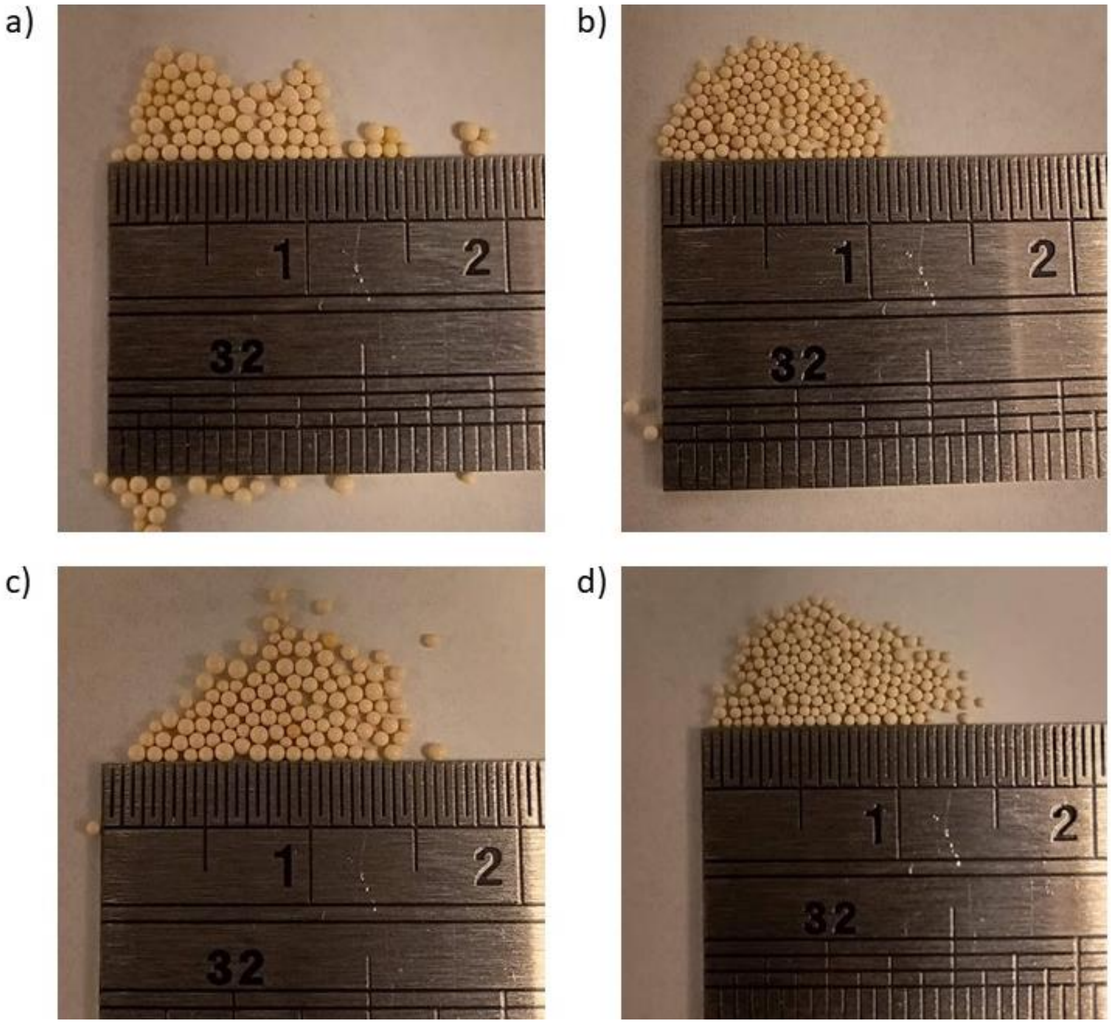

Figure S6. Images of fresh a) Lewatit and b) Purolite beads as well as vacuum-dried c) Lewatit and d) Purolite beads, (degassed ex-situ at 393 K and 0.002 kPa for at least 12 h).

a)

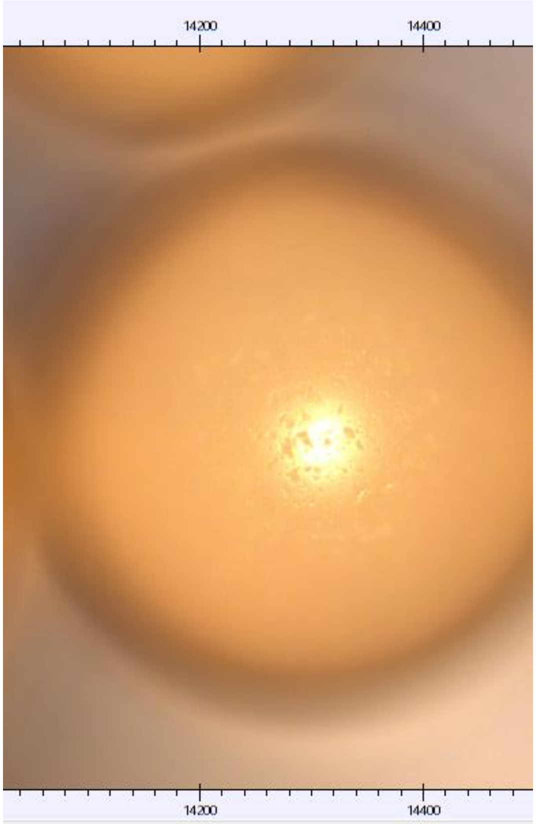

b)

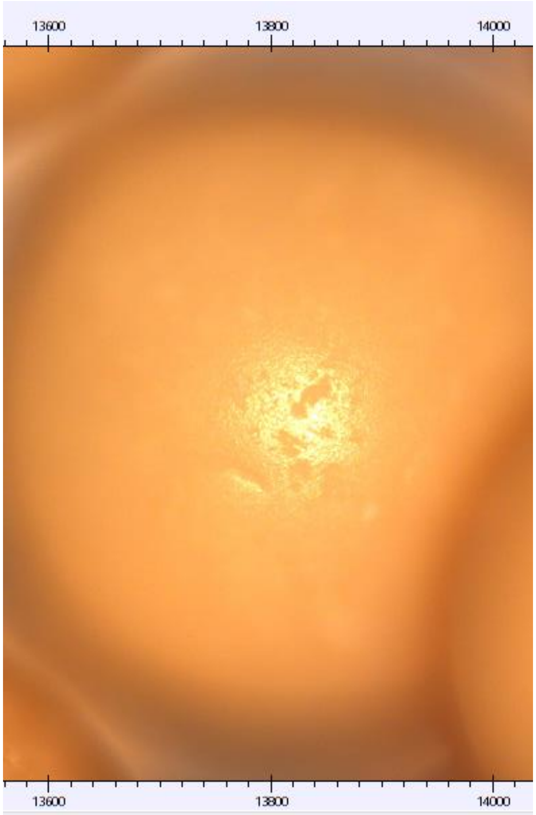

Figure S7. Surface of fresh a) Lewatit and b) Purolite beads under 10× magnification using a Senterra II Raman microscope, where ruler values are in microns.

Table S1. Literature values of textural properties for Lewatit.

Textural property	Value	Reference
$\rho_{skeletal}$ (g cm^{-3})	-	-
$\rho_{particle}$ (g cm^{-3})	0.88	[2]
ρ_{bed} (g cm^{-3})	0.63 (wet)	[3]
V_{total} (cm^3 g^{-1})	0.64	[3]
$\varepsilon_{particle}$	0.56	Calculated using [2] and [3]
S_{BET} (m^2 g^{-1})	43	[3]

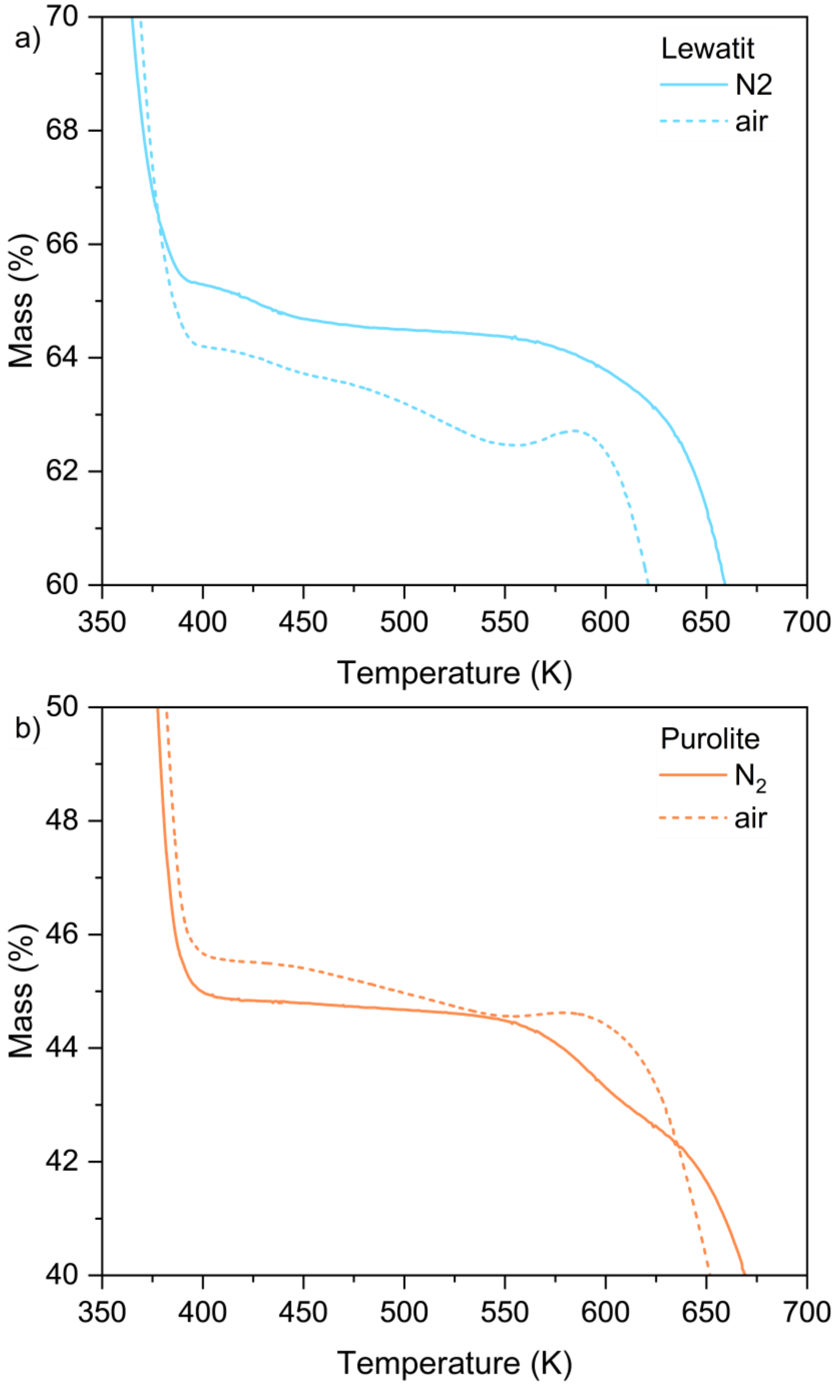

Figure S8. a) TG curves for Lewatit under N_2 and air atmospheres between 373 to 673 K. c) TG curves for Purolite under N_2 and air atmospheres between 373 to 673 K.

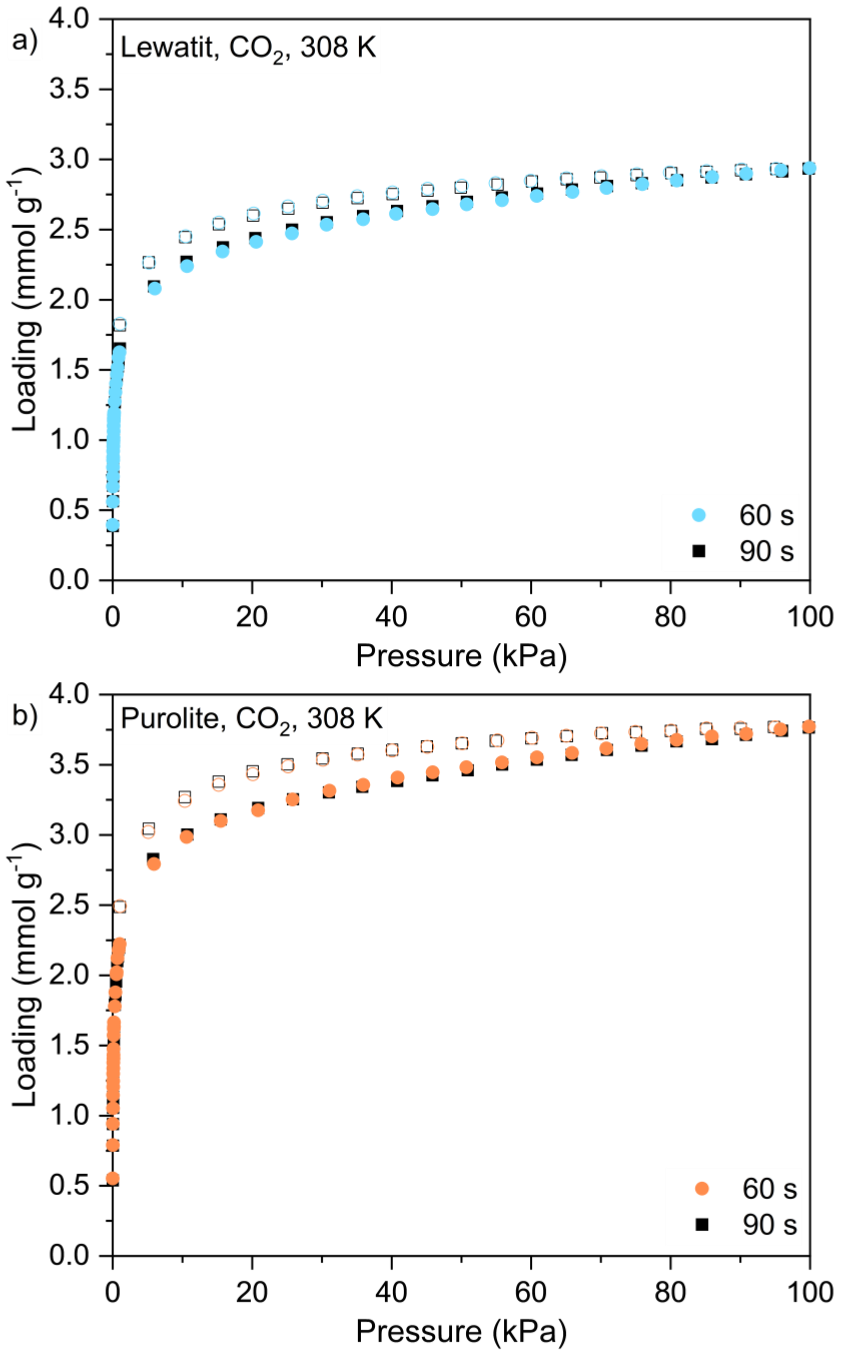

Figure S9. Equilibrium adsorption (filled symbols) and desorption (hollow symbols) isotherms of CO_2 at 308 K for the same sample of a) Lewatit and b) Purolite with 60 s and 90 s equilibration intervals.

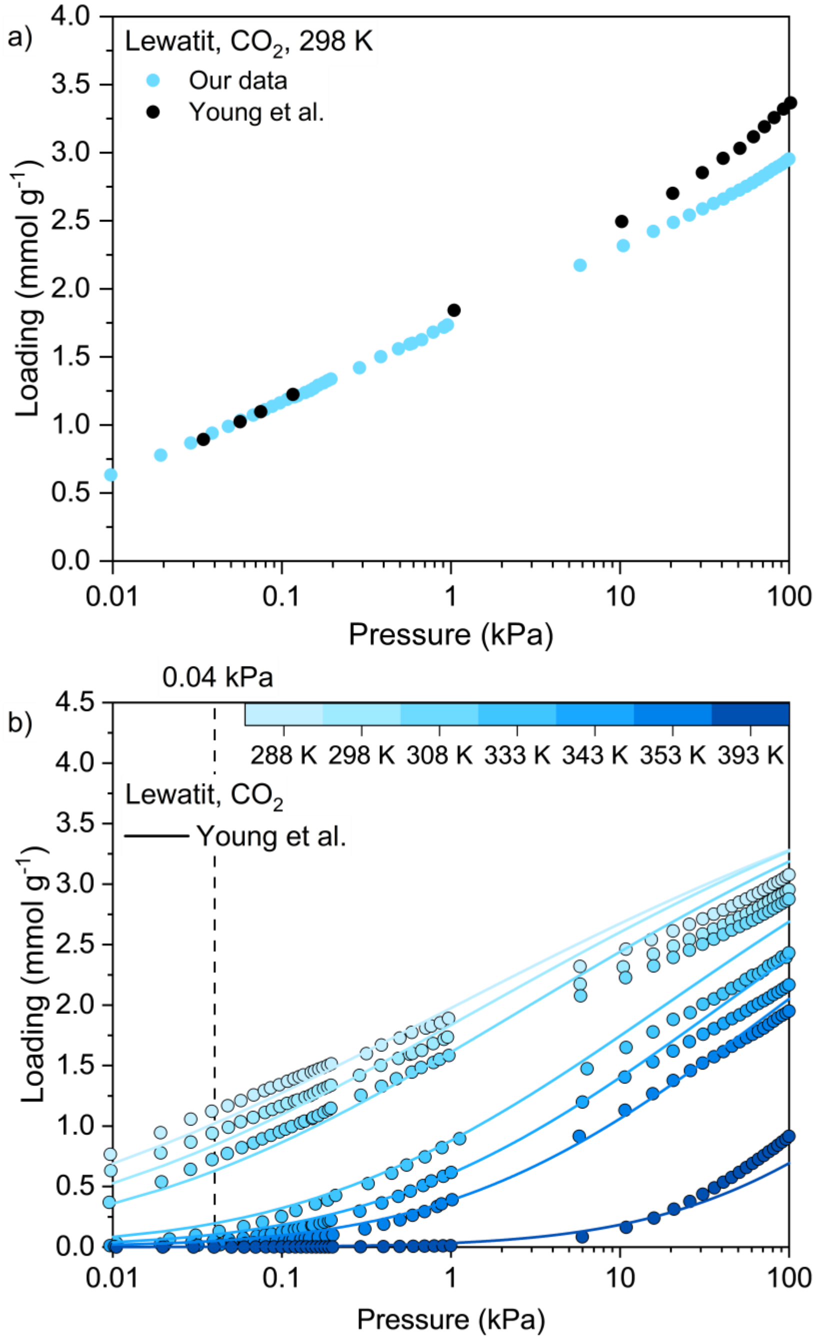

Figure S10. a) Comparison of our measured CO_2 equilibrium adsorption isotherm at 298 K for Lewatit to the 298 K CO_2 isotherm measured by Young et al. [4]. b) Overlay of our measured CO_2 equilibrium adsorption isotherm data with the td-Toth isotherm model and fitting parameters used by Young et al.[4].

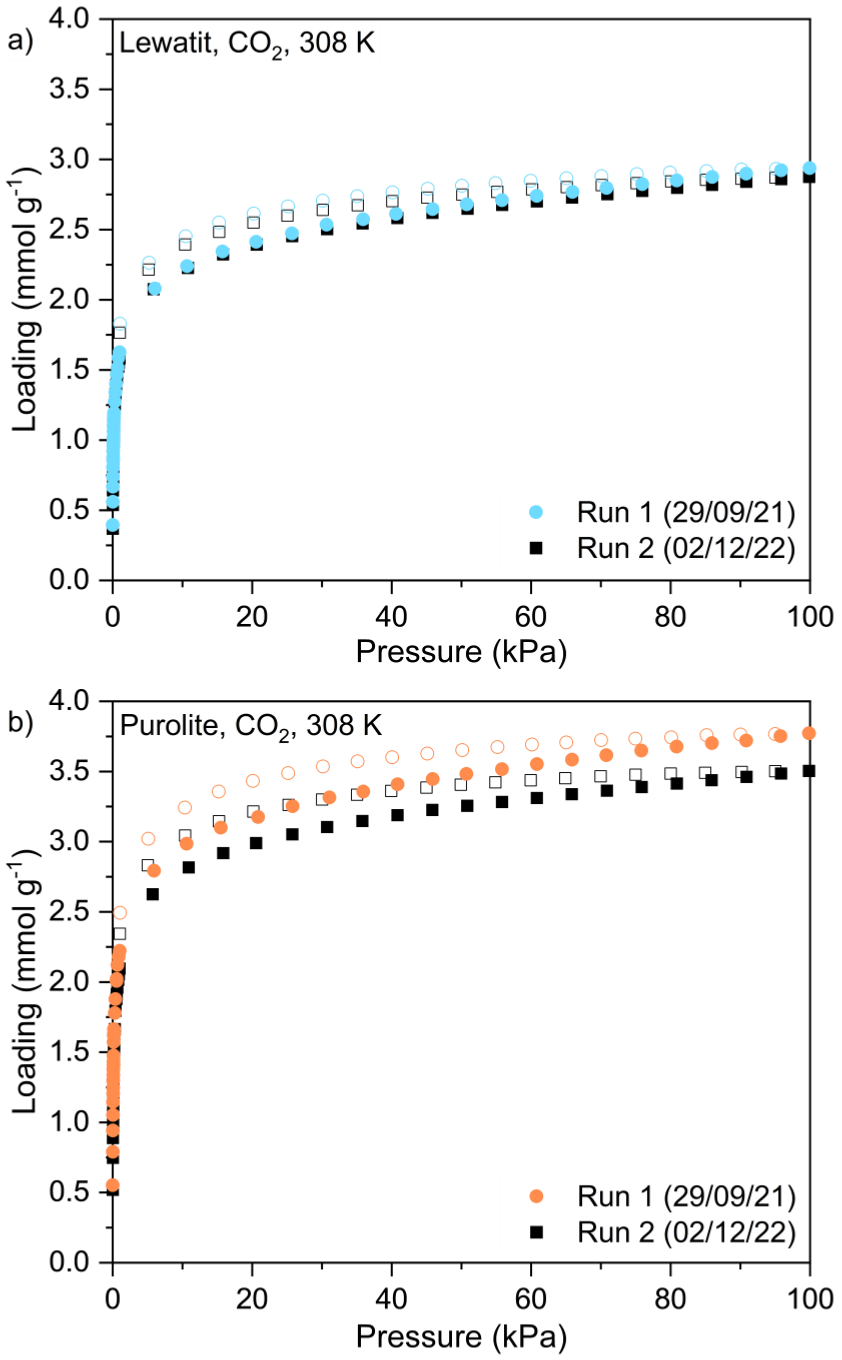

Figure S11. Equilibrium adsorption (filled symbols) and desorption (hollow symbols) isotherms of CO_2 at 308 K for two samples of a) Lewatit and b) Purolite measured approximately one year apart. Each sample is from the same batch of commercial resin.

Table S2. Upper and lower bounds imposed on fitting parameters for the td-Toth, CP, Virial, and SSL isotherm models.

Fitting equation	Parameter	Units	Lower bound	Upper bound
td-Toth	$q_{\infty,0}$	mmol g^{-1}	0	5
	b_0	kPa^{-1}	1×10^{-22}	0.001
	$-\Delta H$	kJ mol^{-1}	0	130
	T_0	K	-	-
	τ_0	-	0	5
	α	-	0	5
	χ	-	0	5
CP	$q_{\infty,0}$	mmol g^{-1}	0	5
	b_0	kPa^{-1}	1×10^{-22}	0.001
	$-\Delta H$	kJ mol^{-1}	0	130
	T_0	K	-	-
	τ_0	-	0	5
	α	-	0	5
	χ	-	0	5
	$q_{\infty c}$	mmol g^{-1}	0	5
	$b_{0,c}$	kPa^{-1}	1×10^{-22}	0.001
	$-\Delta H_c$	kJ mol^{-1}	0	130
	E_a	kJ mol^{-1}	0	50
Virial	a_0	K	0	30 000
	a_1	K mol^{-1}	0	30 000
	a_2	K mol^{-2}	0	30 000
	a_3	K mol^{-3}	0	30 000
	b_0	-	0	30 000
	b_1	mol^{-1}	0	30 000
SSL	q_{∞}	mmol g^{-1}	0	10
	b_0	kPa^{-1}	0	0.01
	$-\Delta H$	kJ mol^{-1}	0	150
GAB	q_m	mmol g^{-1}	0	10
	C	kJ mol^{-1}	0	100
	D	K^{-1}	0	0.05
	F	kJ mol^{-1}	0	100
	G	kJ mol^{-1} K^{-1}	-0.05	0

Table S3. Fitting parameters for the td-Toth isotherm model for CO_2 adsorption for Lewatit used by Young et al. [4].

**Fitting equation**	**Parameter**	**Unit**	**Lewatit**
td-Toth	q_{∞}	mmol g^{-1}	4.86
	b_0	kPa^{-1}	2.85×10^{-18}
	$-\Delta H$	kJ mol^{-1}	117.798
	T_0	K	298.15
	τ_0	-	0.209
	α	-	0.523
	χ	-	0

An F-test was conducted between the fitting results of the td-Toth isotherm model, i.e. the “reduced model”, and the CP isotherm model, i.e. the “full model”, using the following procedure from PennState [5]:

1. Calculate the error sum of squares (SSE) of the CO_2 loading for both the reduced and full model using the following equations:

   $$SSE = \sum(experimental - fitted)^2 \quad (1)$$

2. Calculate the linear F-statistic using the following equation:

   $$F = \left(\frac{SSE(reduced) - SSE(full)}{df_{reduced} - df_{full}}\right) \div \left(\frac{SSE(full)}{df_{full}}\right) \quad (2)$$

   $$df = \#\ experimental\ data\ points - \#\ fitted\ parameters \quad (3)$$

   where *df* stands for degrees of freedom.

3. Check if the calculated F-statistic is larger than the critical value for the given degrees of freedom and a 0.95 confidence level. The critical value can be calculated using the following Excel function:

   $$F.inv\ (probability, df_1, df_2) \quad (4)$$

   where probability is the confidence level, df_1 is the numerator degrees of freedom from equation (2), i.e. $df_{reduced} - df_{full}$, and df_2 is the denominator degrees of freedom from equation (2), i.e. df_{full}.

   If the calculated F-statistic is larger, any better fitting by the full model is not due to an increased number of fitting parameters.

For Lewatit:

$SSE(reduced) = 5.61$

$SSE(full) = 0.20$

$\#\ experimental\ data\ points = 324$

$\#\ fitted\ parameters, reduced = 6$

$\#\ fitted\ parameters, full = 10$

$df_{reduced} = 318$

$df_{full} = 314$

$$F = \left(\frac{SSE(reduced) - SSE(full)}{df_{reduced} - df_{full}}\right) \div \left(\frac{SSE(full)}{df_{full}}\right) = \left(\frac{5.61 - 0.20}{318 - 314}\right) \div \left(\frac{0.20}{314}\right) = 2107$$

$F_{critical}(0.95, 4, 314) = 2.40$

For Lewatit, $F > F_{critical}$, and as such the better fit offered by the CP isotherm model as compared to the td-Toth isotherm model is not due to an increased number of fitting parameters.

For Purolite:

$SSE(reduced) = 5.56$

$SSE(full) = 0.63$

$\# \, experimental \, data \, points = 319$

$\# \, fitted \, parameters, reduced = 6$

$\# \, fitted \, parameters, full = 10$

$df_{reduced} = 313$

$df_{full} = 309$

$$F = \left(\frac{SSE(reduced) - SSE(full)}{df_{reduced} - df_{full}}\right) \div \left(\frac{SSE(full)}{df_{full}}\right) = \left(\frac{5.56 - 0.60}{313 - 309}\right) \div \left(\frac{0.63}{309}\right) = 605$$

$F_{critical}(0.95, 4, 309) = 2.40$

For Purolite, $F > F_{critical}$, and as such the better fit provided by the CP isotherm model compared to the td-Toth isotherm model is not due to an increased number of parameters.

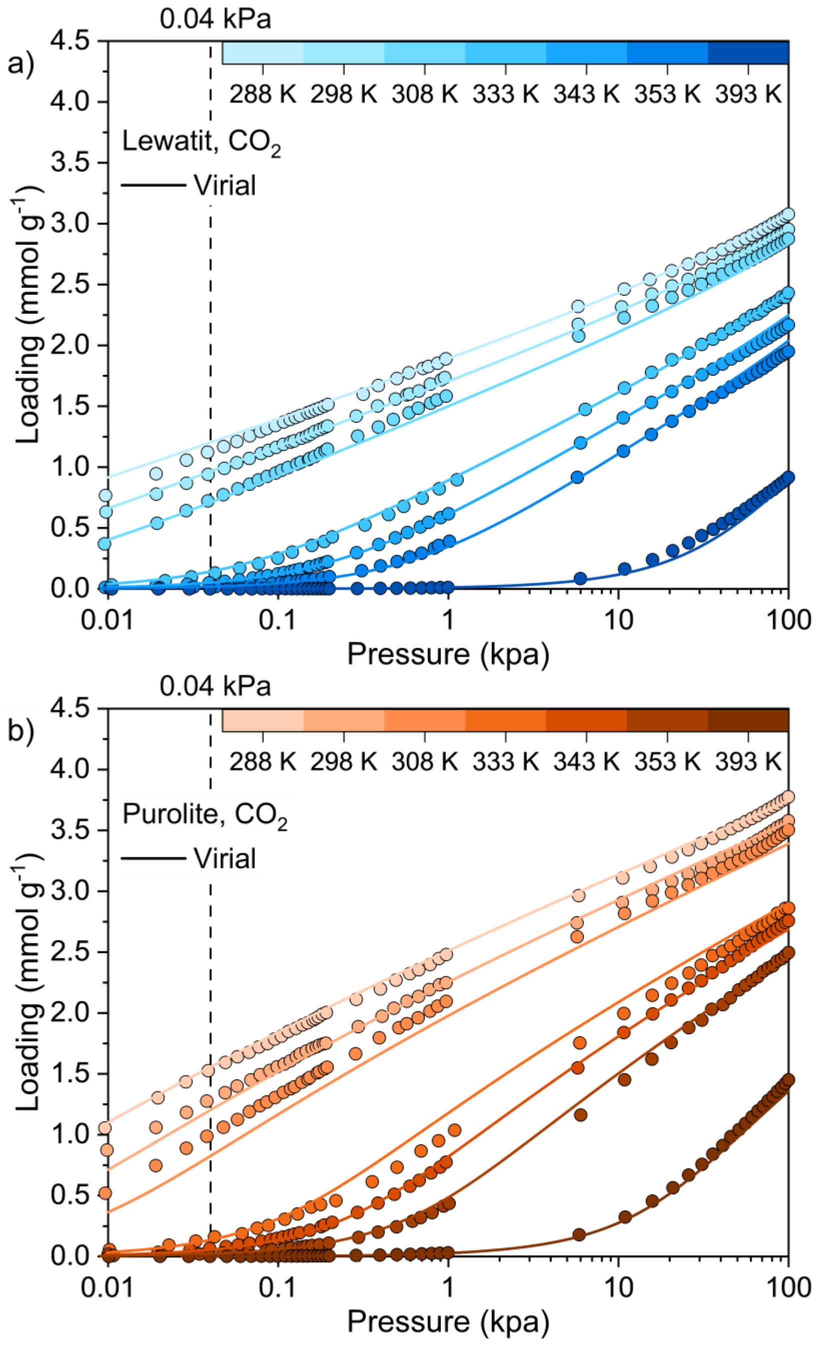

Figure S12. Equilibrium adsorption isotherms for CO_2 measured at 288, 298, 308, 333, 343, 353, and 393 K up to 100 kPa for a) Lewatit and b) Purolite with a log-scale of pressure. Solid lines represent the fitting results from Virial equation, whose fitting parameters are found in Table S4.

Table S4. Fitting parameters with uncertainty bounds for a 95% confidence interval for the Virial equation for CO_2 adsorption for Lewatit and Purolite.

Equation	Parameter	Unit	Lewatit	Purolite
Virial	a_0	K	-12910 ± 20	-10600 ± 30
	a_1	K mol^{-1}	2980 ± 20	850 ± 20
	a_2	K mol^{-2}	269 ± 7	211 ± 6
	a_3	K mol^{-3}	-57 ± 2	-18 ± 1
	b_0	-	32.68 ± 0.07	25.95 ± 0.08
	b_1	mol^{-1}	-7.78 ± 0.05	-2.29 ± 0.05

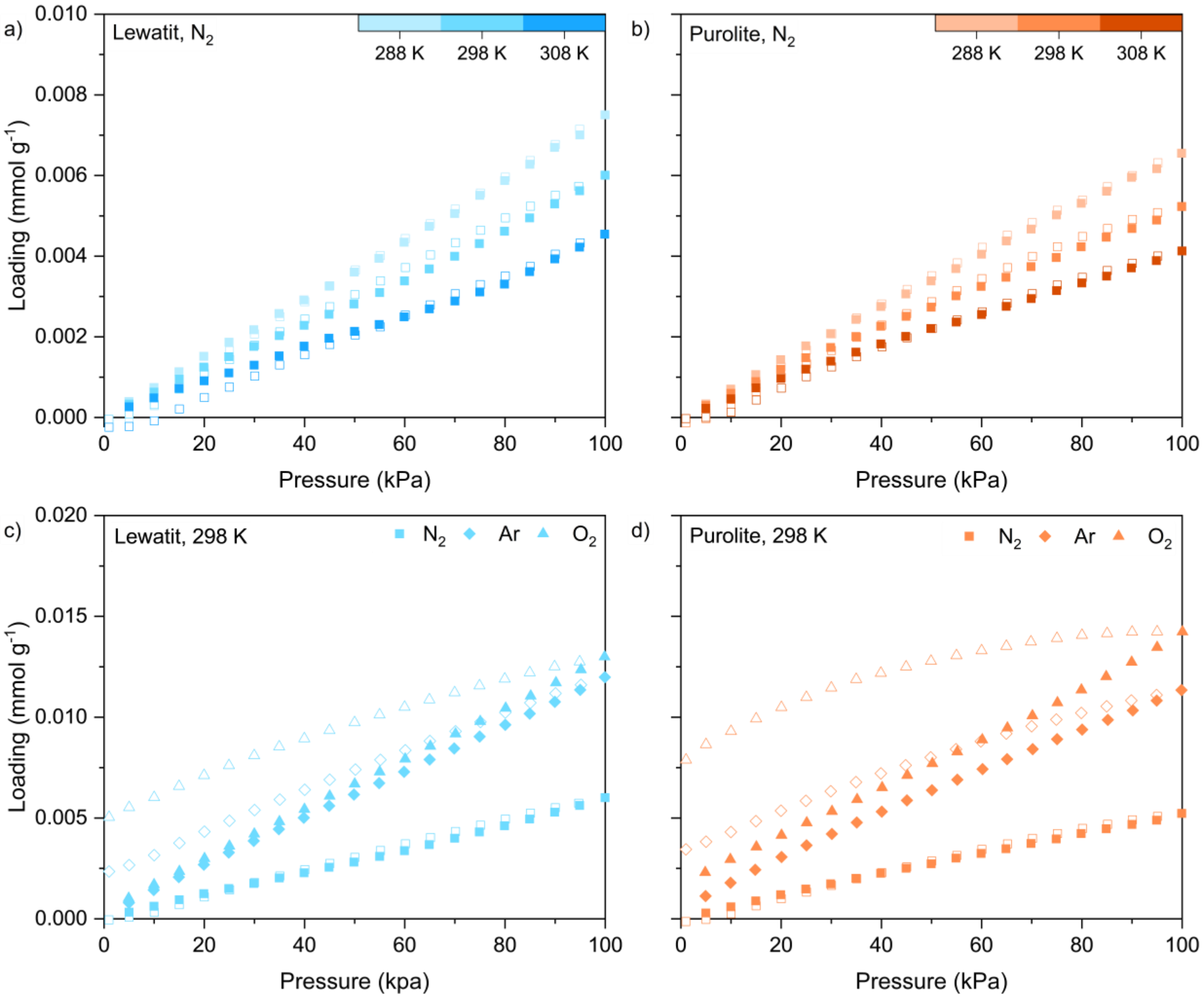

Figure S13. Equilibrium adsorption (filled symbols) and desorption (hollow symbols) isotherms for N_2 measured at 288, 298, and 308 K up to 100 kPa for a) Lewatit and b) Purolite. Equilibrium adsorption (filled symbols) and desorption (hollow symbols) isotherms for N_2, Ar, and O_2 measured at 298 K up to 100 kPa for c) Lewatit and d) Purolite.

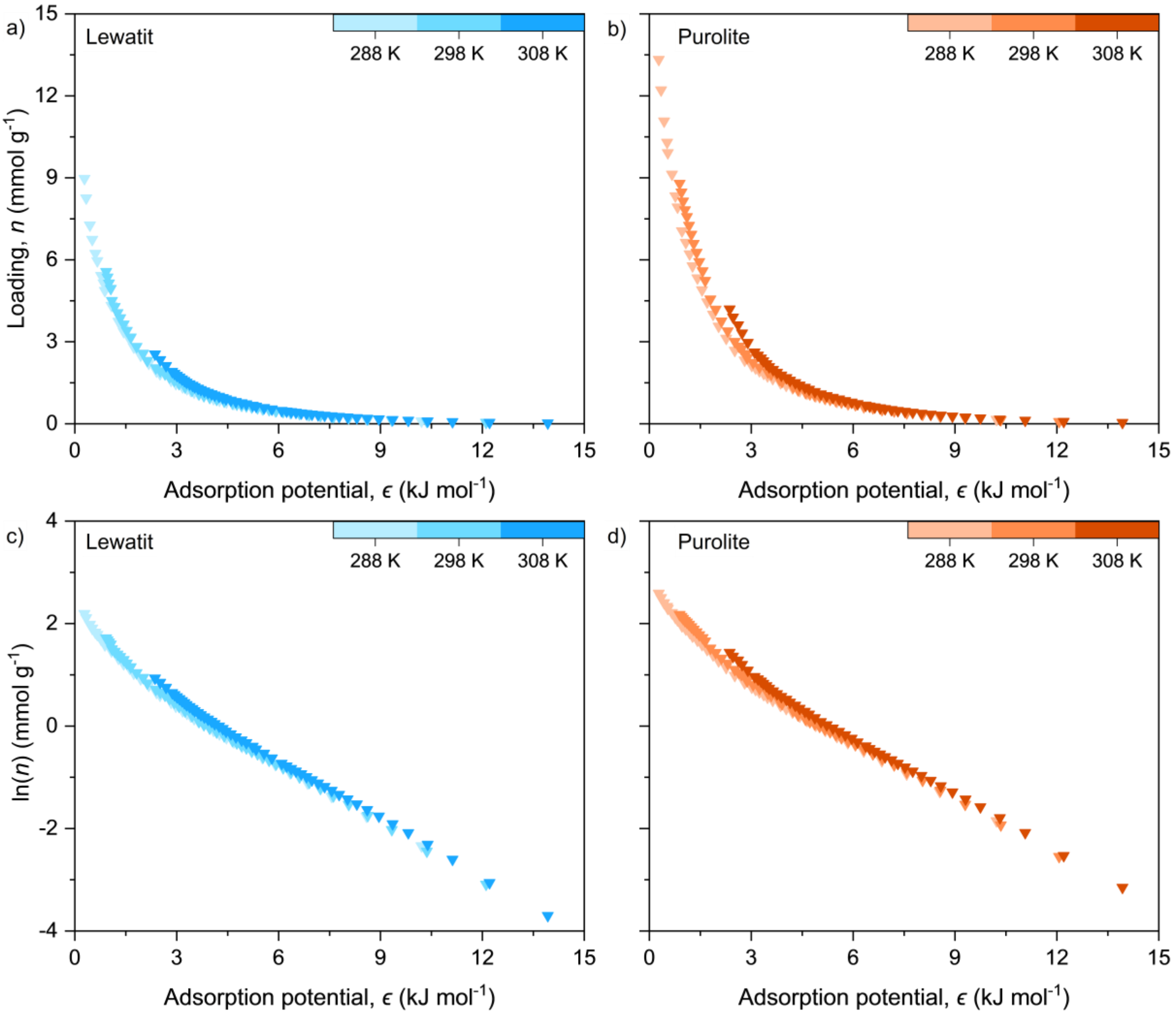

Figure S14. H_2O adsorption potentials for Lewatit plotted against the a) H_2O loading and c) natural log of the H_2O loading. H_2O adsorption potentials for Purolite plotted against the b) H_2O loading and d) natural log of the H_2O loading.

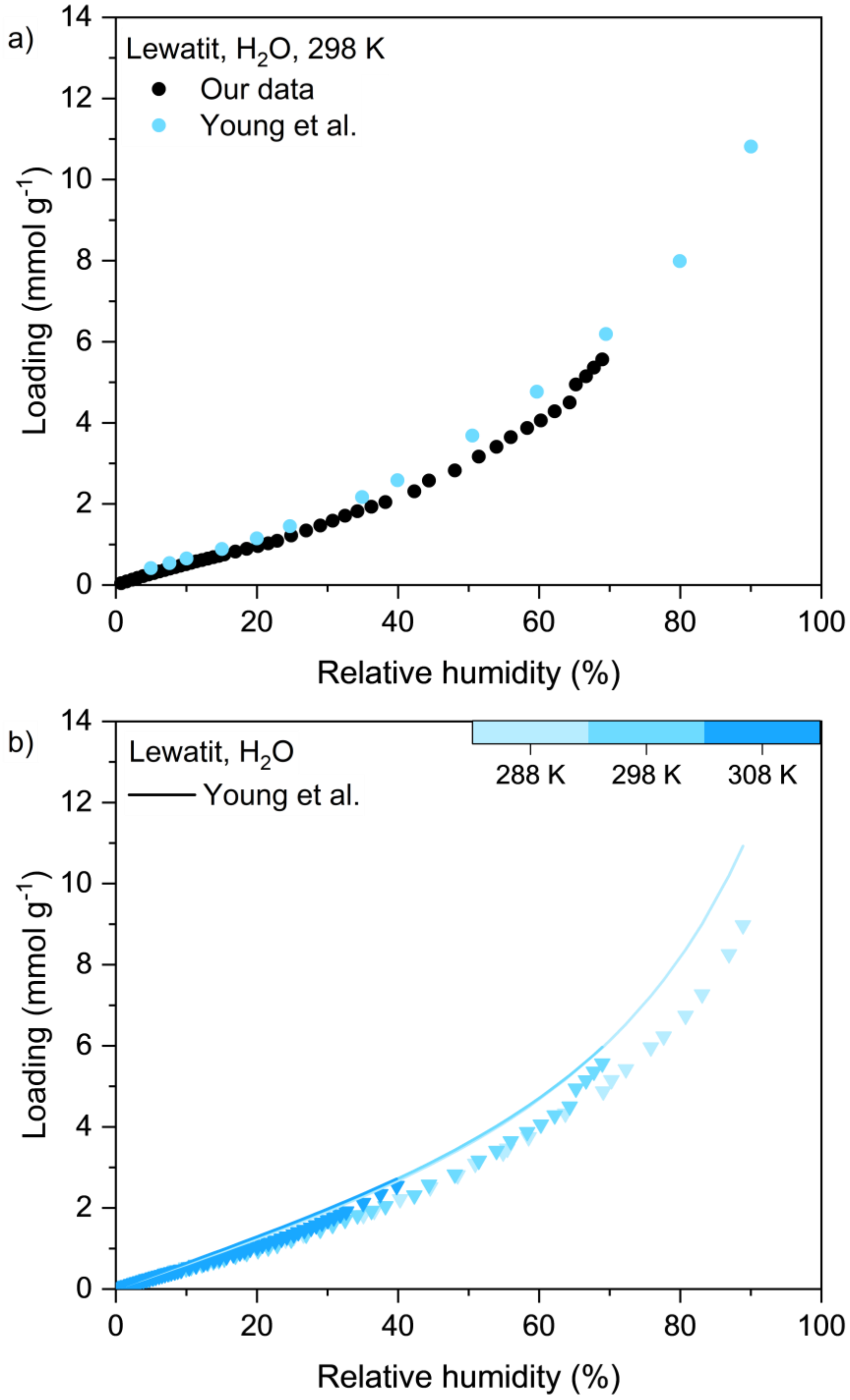

Figure S15. a) Comparison of our measured H_2O equilibrium adsorption isotherm at 298 K for Lewatit to the 298 K H_2O isotherm measured by Young et al. [4] b) Overlay of our measured H_2O equilibrium adsorption isotherm data with the GAB isotherm model and fitting parameters used by Young et al. [4].

Table S5. Fitting parameters for the GAB isotherm model for H_2O adsorption for Lewatit used by Young et al. [4].

**Fitting equation**	**Parameter**	**Unit**	**Lewatit**
GAB	q_m	mmol g^{-1}	3.63
	C	kJ mol^{-1}	47.110
	D	K^{-1}	0.023744
	F	kJ mol^{-1}	57.706
	G	kJ mol^{-1} K^{-1}	-0.047814

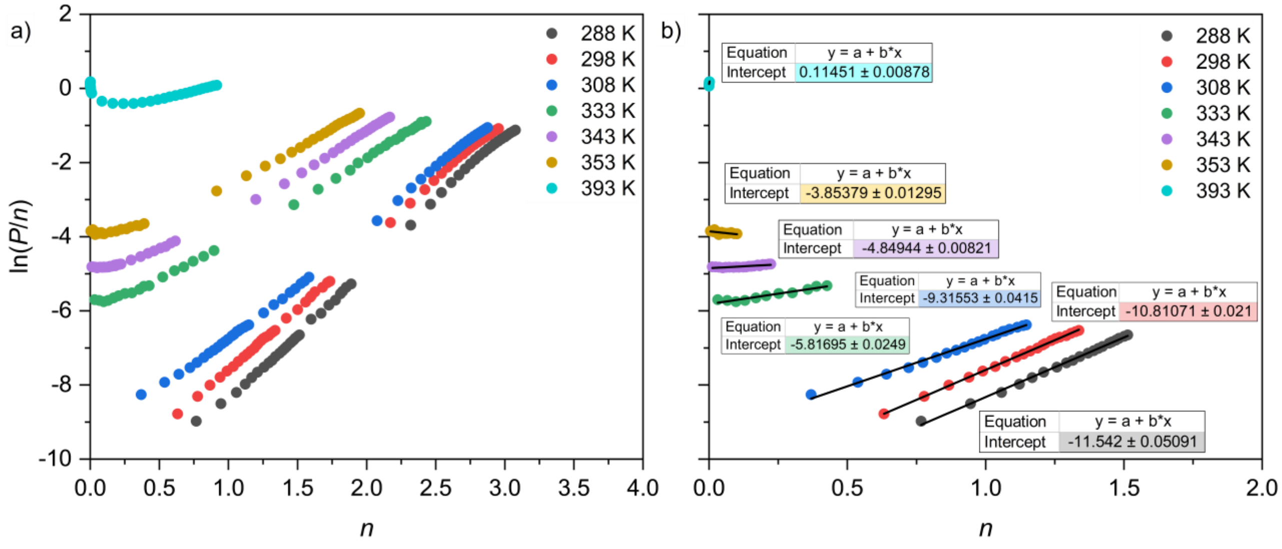

c)

y-intercept, a	K = exp(-a)	T	1/T	ln(K)
-11.542	102950.56433	288.15	0.00347	11.542
-10.81071	49548.71943	298.15	0.00335	10.81071
-9.31553	11109.23956	308.15	0.00325	9.31553
-5.81695	335.94585	333.15	0.003	5.81695
-4.84944	127.66888	343.15	0.00291	4.84944
-3.85379	47.1715	353.15	0.00283	3.85379
0.11451	0.8918	393.15	0.00254	-0.11451

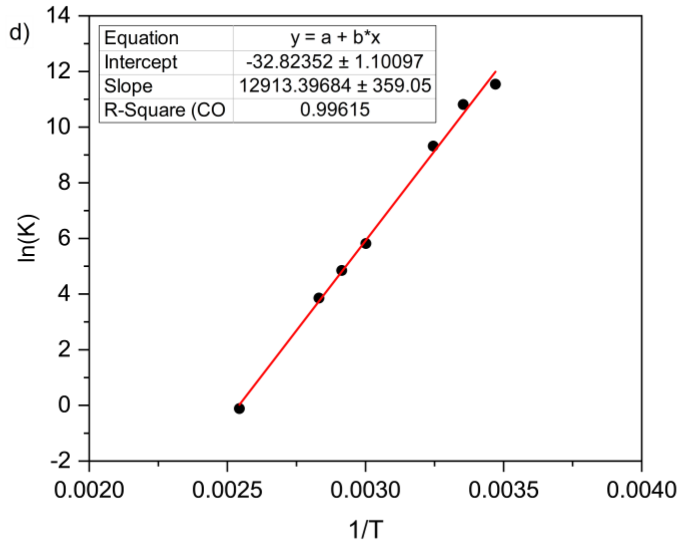

Figure S16. a) Virial plot of all measured CO_2 isotherm data for Lewatit, where pressure (*P*), loading (*n*), and temperature (*T*) values are in units of bar, mmol g^{-1}, and K, respectively. b) Virial plot showing the selected low loading data fitted to linear equations. The y-intercepts of each fitted equation are used to calculate the Henry constant K. c) Summary of fitted and calculated parameters used to determine ΔH_0. d) Plot of lnK vs. 1/T fitted to a linear equation, whose slope is used to calculate ΔH_0.

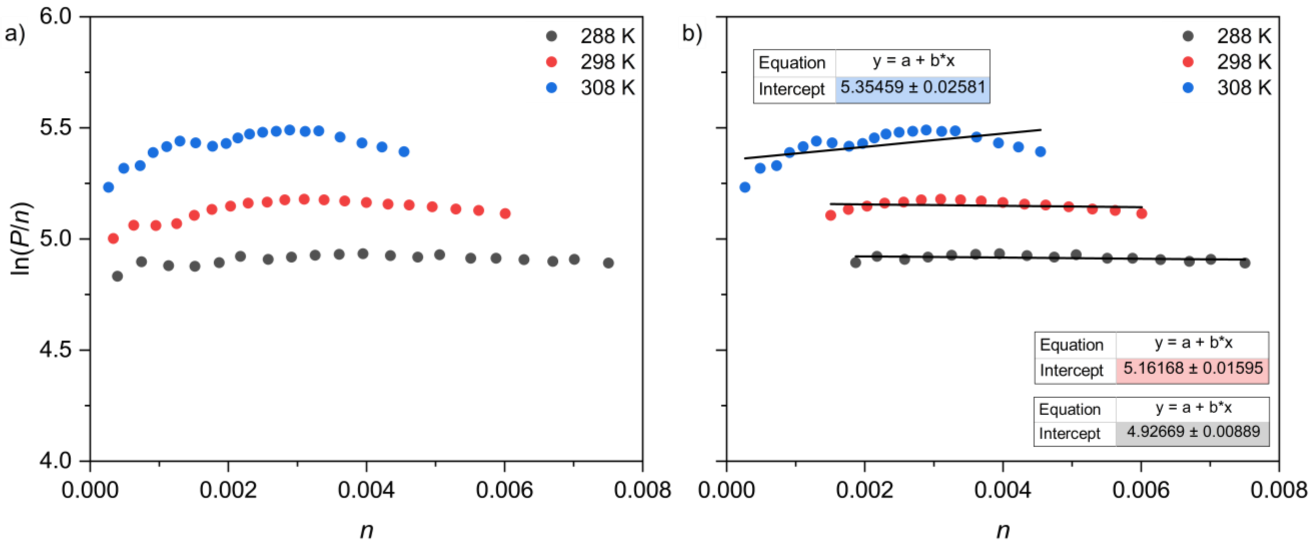

c)

y-intercept, a	K = exp(-a)	T	1/T	ln(K)
4.92669	0.00725	288.15	0.00347	-4.92669
5.16168	0.00573	298.15	0.00335	-5.16168
5.35459	0.00473	308.15	0.00325	-5.35459

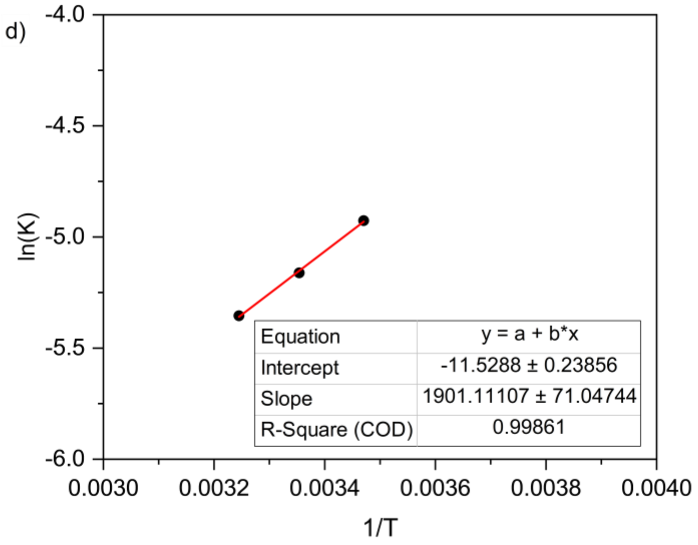

Figure S17. a) Virial plot of all measured N_2 isotherm data for Lewatit, where pressure (*P*), loading (*n*), and temperature (*T*) values are in units of bar, mmol g^{-1}, and K, respectively. b) Virial plot showing the selected low loading data fitted to linear equations. The y-intercepts of each fitted equation are used to calculate the Henry constant K. c) Summary of fitted and calculated parameters used to determine ΔH_0. d) Plot of lnK vs. 1/T fitted to a linear equation, whose slope is used to calculate ΔH_0.

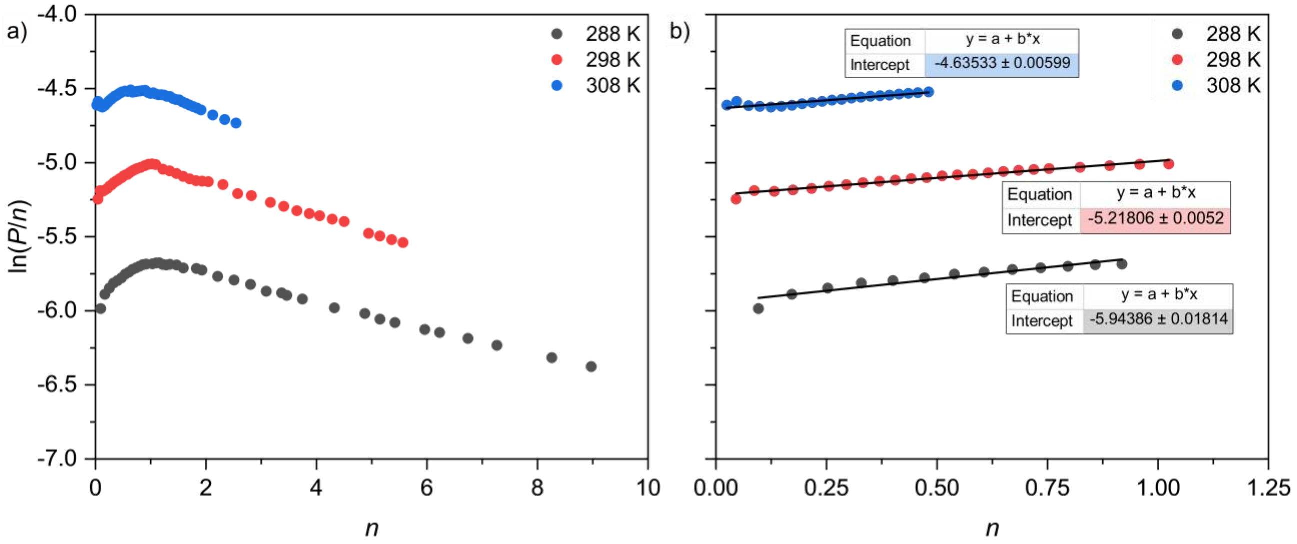

c)

y-intercept, a	K = exp(-a)	T	1/T	ln(K)
-5.94386	381.40431	288.25	0.00347	5.94386
-5.21806	184.57576	298.15	0.00335	5.21806
-4.63533	103.06192	308.15	0.00325	4.63533

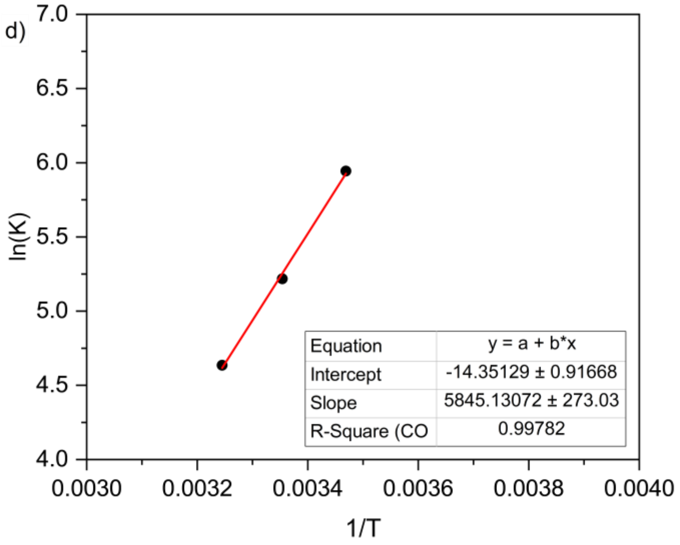

Figure S18. a) Virial plot of all measured H_2O isotherm data for Lewatit, where pressure (*P*), loading (*n*), and temperature (*T*) values are in units of bar, mmol g^{-1}, and K, respectively. b) Virial plot showing the selected low loading data fitted to linear equations. The y-intercepts of each fitted equation are used to calculate the Henry constant K. c) Summary of fitted and calculated parameters used to determine ΔH_0. d) Plot of lnK vs. 1/T fitted to a linear equation, whose slope is used to calculate ΔH_0.

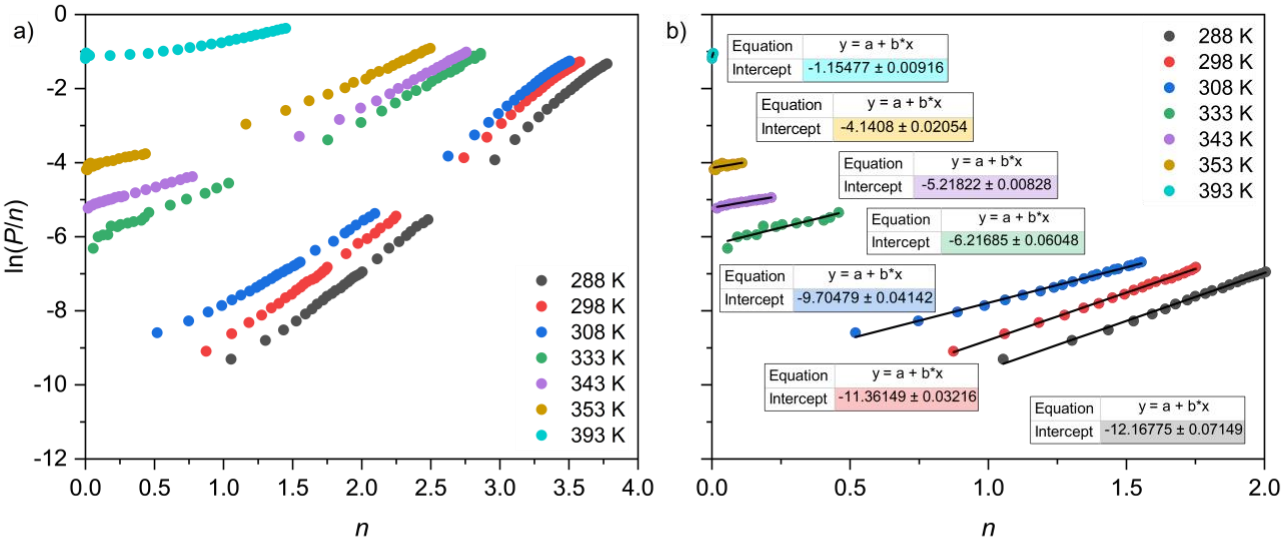

c)

y-intercept, a	K = exp(-a)	T	1/T	ln(K)
-12.16775	192480.47519	288.15	0.00347	12.16775
-11.36149	85947.33466	298.15	0.00335	11.36149
-9.70479	16395.95603	308.15	0.00325	9.70479
-6.21685	501.12221	333.15	0.003	6.21685
-5.21822	184.60529	343.15	0.00291	5.21822
-4.1408	62.85308	353.15	0.00283	4.1408
-1.15477	3.17329	393.15	0.00254	1.15477

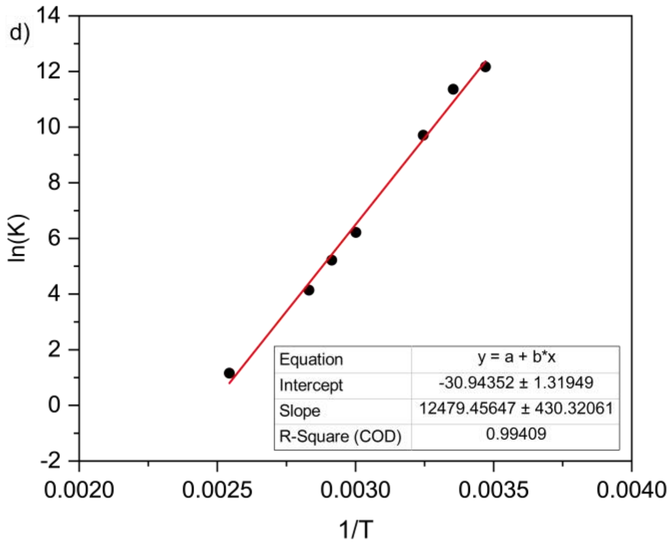

Figure S19. a) Virial plot of all measured CO_2 isotherm data for Purolite, where pressure (*P*), loading (*n*), and temperature (*T*) values are in units of bar, mmol g^{-1}, and K, respectively. b) Virial plot showing the selected low loading data fitted to linear equations. The y-intercepts of each fitted equation are used to calculate the Henry constant K. c) Summary of fitted and calculated parameters used to determine ΔH_0. d) Plot of lnK vs. 1/T fitted to a linear equation, whose slope is used to calculate ΔH_0.

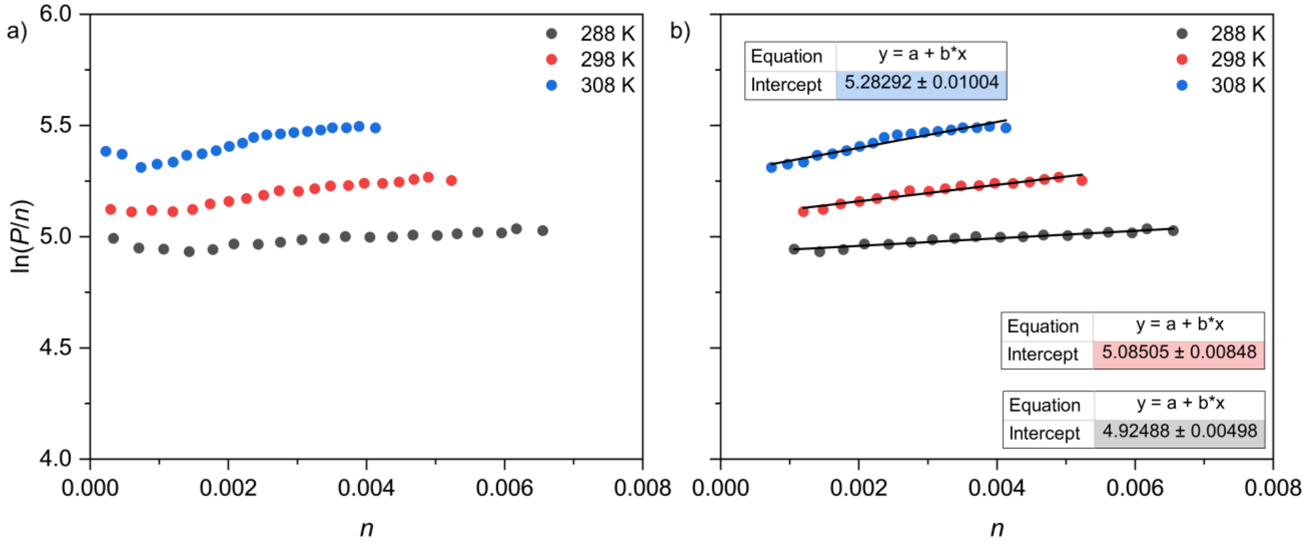

c)

y-intercept, a	K = exp(-a)	T	1/T	ln(K)
4.92488	0.00726	288.15	0.00347	-4.92488
5.08505	0.00619	298.15	0.00335	-5.08505
5.28292	0.00508	308.15	0.00325	-5.28292

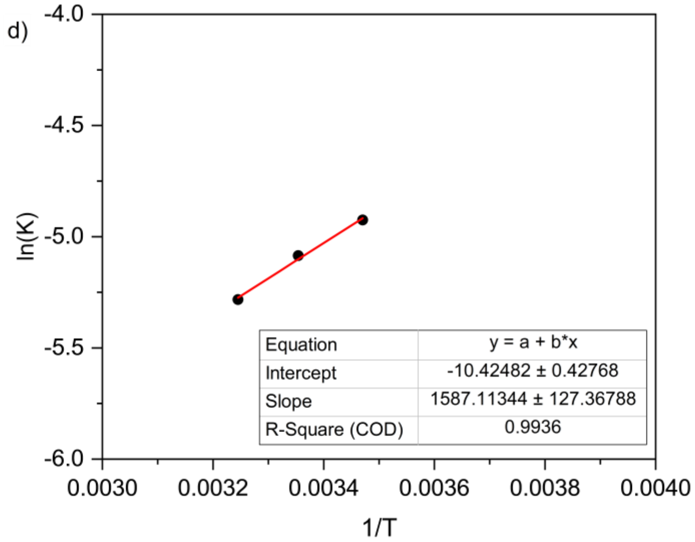

Figure S20. a) Virial plot of all measured N_2 isotherm data for Purolite, where pressure (*P*), loading (*n*), and temperature (*T*) values are in units of bar, mmol g^{-1}, and K, respectively. b) Virial plot showing the selected low loading data fitted to linear equations. The y-intercepts of each fitted equation are used to calculate the Henry constant K. c) Summary of fitted and calculated parameters used to determine ΔH_0. d) Plot of lnK vs. 1/T fitted to a linear equation, whose slope is used to calculate ΔH_0.

a)
b)

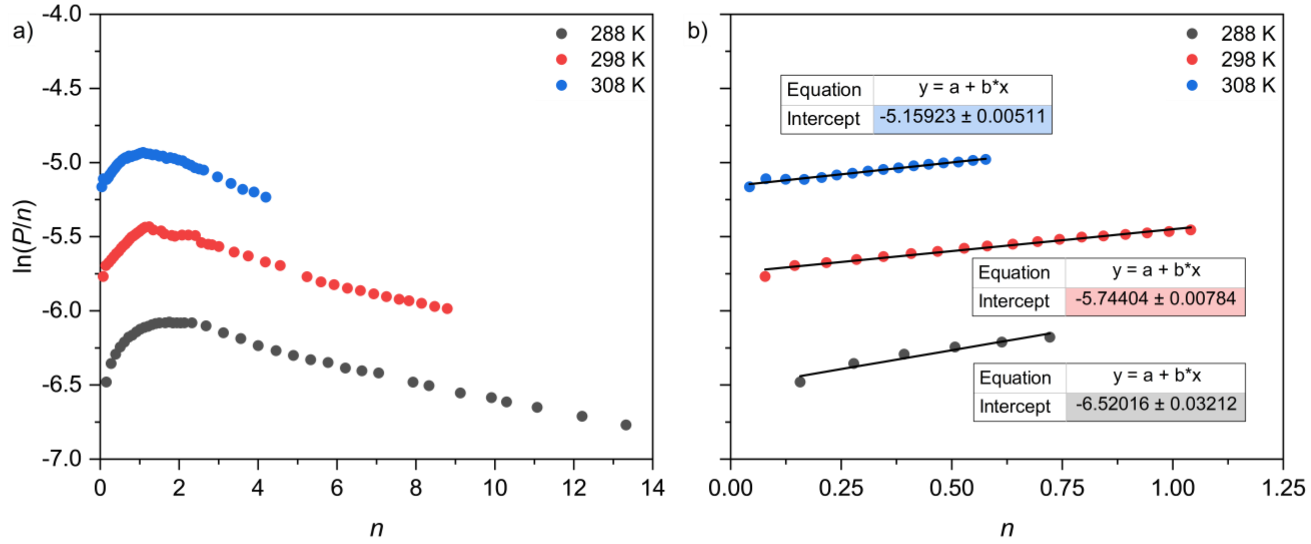

c)

y-intercept, a	K = exp(-a)	T	1/T	ln(K)
-6.52016	678.68697	288.25	0.00347	6.52016
-5.74404	312.32365	298.15	0.00335	5.74404
-5.15923	174.0304	308.15	0.00325	5.15923

d)

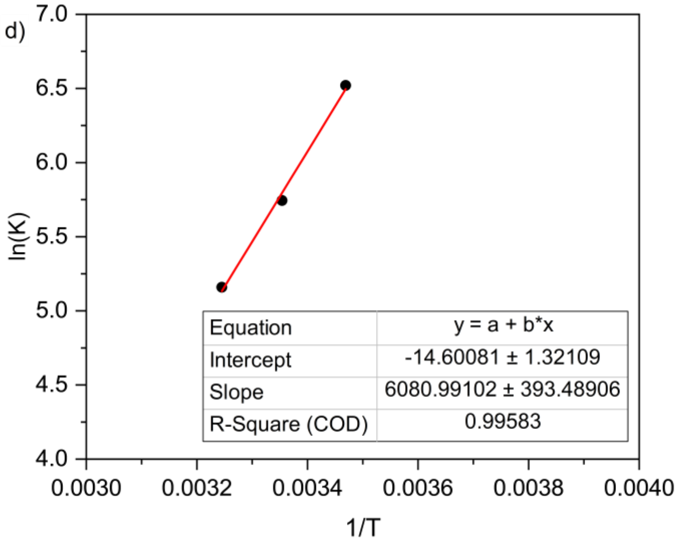

Figure S21. a) Virial plot of all measured H_2O isotherm data for Purolite, where pressure (*P*), loading (*n*), and temperature (*T*) values are in units of bar, mmol g^{-1}, and K, respectively. b) Virial plot showing the selected low loading data fitted to linear equations. The y-intercepts of each fitted equation are used to calculate the Henry constant K. c) Summary of fitted and calculated parameters used to determine ΔH_0. d) Plot of lnK vs. 1/T fitted to a linear equation, whose slope is used to calculate ΔH_0.

Table S6. Smoothing spline values used in MATLAB for the interpolation of CO_2, N_2, and H_2O data to calculate the corresponding isosteric heats of adsorption for Lewatit and Purolite.

	**Lewatit**	**Purolite**
CO_2	0.9989	0.9997
N_2	0.9999999	0. 9999999999
H_2O	0.919	0.670

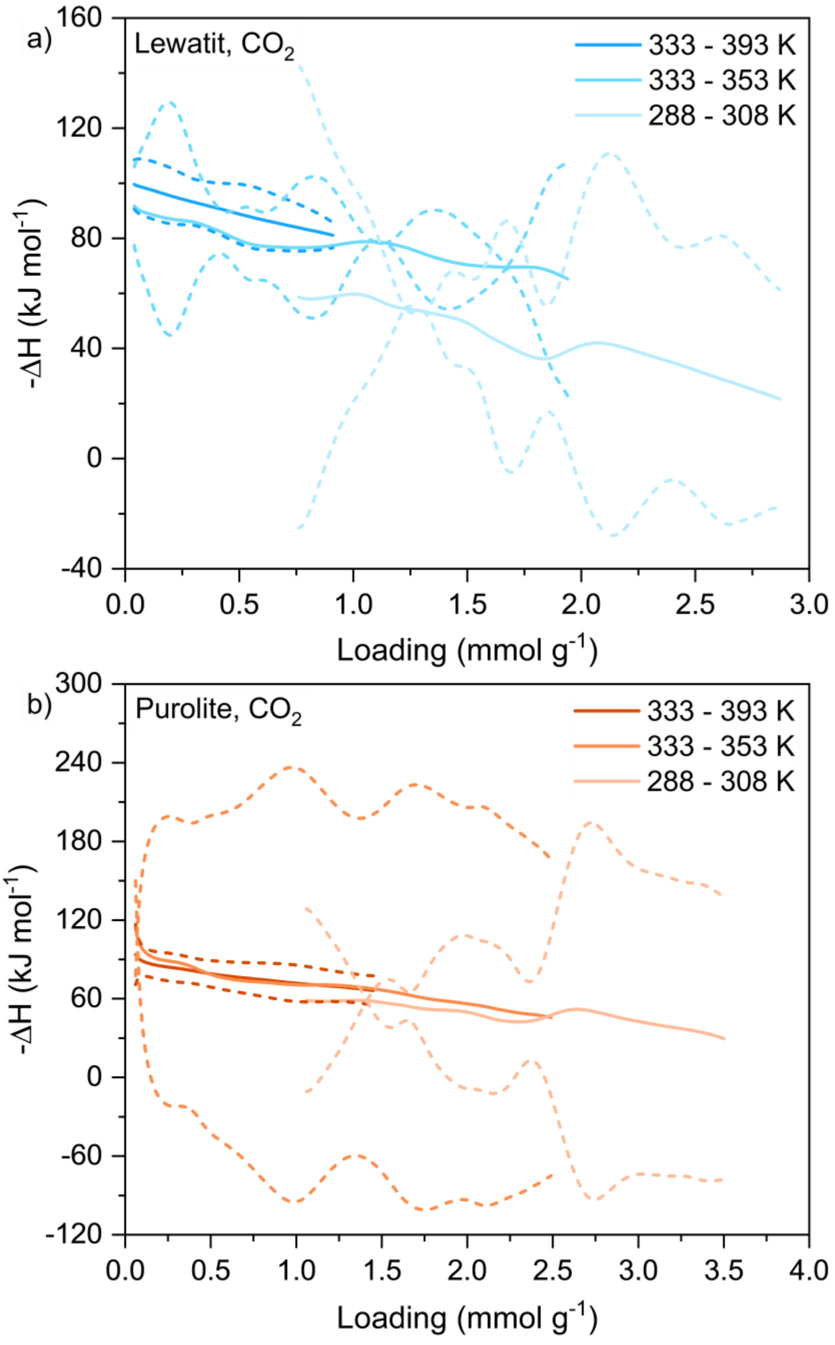

Figure S22. Isosteric heats of adsorption (solid lines) of a) Lewatit and b) Purolite for CO_2 calculated using isotherm data measured at 333, 343, 353, and 393 K, at 333, 343, and 353 K, and288, 298, and 308 K. Dashed lines represent the upper and lower confidence intervals.

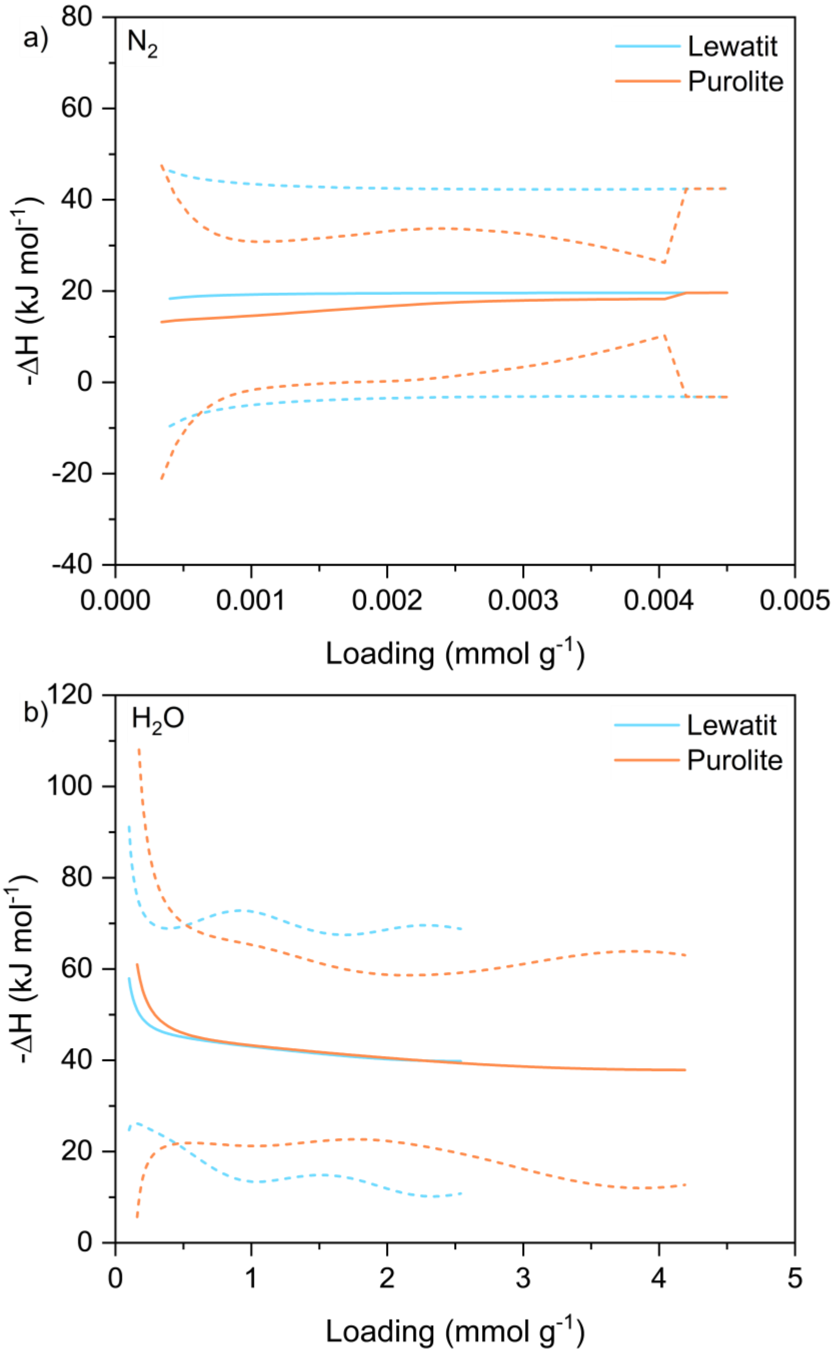

Figure S23. Isosteric heats of adsorption (solid lines) of Lewatit and Purolite for a) N_2 and b) H_2O with corresponding upper and lower confidence intervals (dashed lines)

References

[1] NIST. "Aluminium Oxide." https://webbook.nist.gov/cgi/cbook.cgi?ID=C1344281&Type=JANAFS&Plot=on (accessed 2023).

[2] R. Veneman, T. Hilbers, D. W. F. Brilman, and S. R. A. Kersten, "CO_2 capture in a continuous gas–solid trickle flow reactor," *Chemical Engineering Journal*, vol. 289, pp. 191-202, 2016, doi: 10.1016/j.cej.2015.12.066.

[3] Lanxess. "Product Information Lewatit VP OC 1065." https://lanxess.com/en/Products-and-Brands/Products/l/LEWATIT--VP-OC-1065 (accessed 2023).

[4] J. Young, E. García-Díez, S. Garcia, and M. van der Spek, "The impact of binary water–CO_2 isotherm models on the optimal performance of sorbent-based direct air capture processes," *Energy & Environmental Science*, vol. 14, no. 10, pp. 5377-5394, 2021, doi: 10.1039/d1ee01272j.

[5] PennState. "6.2 The General Linear F-Test." https://online.stat.psu.edu/stat501/lesson/6/6.2 (accessed 2023).
