# Supplementary material for: Measurement of Physicochemical Properties and CO2, N2, Ar, O2, and H2O Unary Adsorption Isotherms of Purolite A110 and Lewatit VP OC 1065 for Application in Direct Air Capture
Source: J Chem Eng Data. 2023 Oct 11;68(12):3499–511. doi: 10.1021/acs.jced.3c00401 (PMC10726313; doi:10.1021/acs.jced.3c00401)
Supplement: Supplementary file 4 — je3c00401_si_004.pdf [file je3c00401_si_004.pdf]

Table A.1. Adsorbed Amounts of $CO_2$ on Lewatit and Purolite at 288, 298, 308, 333, 343, 353, and 393 K up to 100 kPa

| Lewatit | | Purolite | |
|---|---|---|---|
| pressure (kPa) | loading (mmol $g^{-1}$) | pressure (kPa) | loading (mmol $g^{-1}$) |
| 288.15 K | | | |
| 0.0097 | 0.7671 | 0.0096 | 1.0541 |
| 0.0192 | 0.9457 | 0.0196 | 1.3031 |
| 0.0289 | 1.0577 | 0.0287 | 1.4346 |
| 0.0384 | 1.1222 | 0.0388 | 1.5263 |
| 0.0478 | 1.1683 | 0.0489 | 1.5935 |
| 0.0571 | 1.2099 | 0.0577 | 1.6407 |
| 0.0680 | 1.2577 | 0.0676 | 1.6779 |
| 0.0787 | 1.2923 | 0.0771 | 1.7206 |
| 0.0857 | 1.3158 | 0.0867 | 1.7491 |
| 0.0965 | 1.3406 | 0.0955 | 1.7857 |
| 0.1081 | 1.3703 | 0.1046 | 1.8103 |
| 0.1177 | 1.3912 | 0.1148 | 1.8485 |
| 0.1264 | 1.4076 | 0.1272 | 1.8736 |
| 0.1344 | 1.4227 | 0.1390 | 1.9017 |
| 0.1492 | 1.4462 | 0.1492 | 1.9197 |
| 0.1558 | 1.4562 | 0.1602 | 1.9406 |
| 0.1653 | 1.4714 | 0.1661 | 1.9536 |
| 0.1747 | 1.4824 | 0.1725 | 1.9683 |
| 0.1867 | 1.5009 | 0.1819 | 1.9895 |
| 0.1959 | 1.5139 | 0.1921 | 2.0040 |
| 0.3163 | 1.5984 | 0.2877 | 2.1123 |
| 0.3874 | 1.6704 | 0.3995 | 2.1975 |
| 0.4989 | 1.7266 | 0.4920 | 2.2702 |
| 0.5863 | 1.7748 | 0.5935 | 2.3232 |
| 0.7098 | 1.8106 | 0.6698 | 2.3547 |
| 0.7644 | 1.8261 | 0.7835 | 2.3981 |
| 0.8660 | 1.8530 | 0.8807 | 2.4463 |
| 0.9673 | 1.8884 | 0.9739 | 2.4798 |
| 5.7959 | 2.3199 | 5.8422 | 2.9639 |
| 10.8305 | 2.4626 | 10.6079 | 3.1096 |
| 15.3338 | 2.5414 | 15.3981 | 3.2031 |
| 20.6030 | 2.6120 | 20.6317 | 3.2815 |
| 25.7155 | 2.6672 | 25.7583 | 3.3407 |
| 30.8473 | 2.7124 | 30.7008 | 3.3929 |
| 35.8193 | 2.7507 | 35.9016 | 3.4352 |
| 40.8226 | 2.7861 | 40.8176 | 3.4742 |
| 45.8620 | 2.8174 | 45.8937 | 3.5123 |
| 50.8162 | 2.8500 | 50.7967 | 3.5428 |
| 55.8449 | 2.8763 | 55.9044 | 3.5712 |
| 60.8271 | 2.9035 | 60.8606 | 3.5973 |
| 65.9129 | 2.9274 | 65.8585 | 3.6216 |
| 70.8630 | 2.9523 | 70.8525 | 3.6479 |
| 75.8958 | 2.9743 | 75.8173 | 3.6718 |
| 80.8332 | 3.0003 | 80.8161 | 3.6934 |
| 85.8966 | 3.0190 | 85.8397 | 3.7144 |
| 90.8093 | 3.0383 | 90.8357 | 3.7394 |
| 95.8105 | 3.0601 | 95.8889 | 3.7577 |
| 99.8681 | 3.0769 | 99.8477 | 3.7754 |
| 298.15 K | | | |
| 0.0097 | 0.6324 | 0.0098 | 0.8742 |
| 0.0192 | 0.7778 | 0.0191 | 1.0591 |
| 0.0289 | 0.8671 | 0.0289 | 1.1836 |
| 0.0388 | 0.9400 | 0.0381 | 1.2762 |
| 0.0483 | 0.9897 | 0.0487 | 1.3456 |
| 0.0570 | 1.0350 | 0.0574 | 1.4004 |
| 0.0677 | 1.0718 | 0.0692 | 1.4495 |
| 0.0794 | 1.1114 | 0.0783 | 1.4933 |
| 0.0878 | 1.1368 | 0.0895 | 1.5270 |
| 298.15 K | | | |
| 0.0973 | 1.1613 | 0.0988 | 1.5583 |
| 0.1074 | 1.1873 | 0.1088 | 1.5809 |
| 0.1186 | 1.2060 | 0.1181 | 1.6064 |
| 0.1242 | 1.2150 | 0.1280 | 1.6405 |
| 0.1372 | 1.2370 | 0.1349 | 1.6635 |
| 0.1467 | 1.2526 | 0.1464 | 1.6864 |
| 0.1543 | 1.2683 | 0.1586 | 1.7088 |
| 0.1639 | 1.2918 | 0.1679 | 1.7218 |
| 0.1766 | 1.3089 | 0.1750 | 1.7360 |
| 0.1869 | 1.3252 | 0.1850 | 1.7461 |
| 0.1953 | 1.3375 | 0.1908 | 1.7510 |
| 0.2880 | 1.4194 | 0.2956 | 1.8875 |
| 0.3844 | 1.5012 | 0.4074 | 1.9730 |
| 0.4898 | 1.5588 | 0.4793 | 2.0396 |
| 0.5700 | 1.5930 | 0.5715 | 2.0970 |
| 0.5914 | 1.5999 | 0.6687 | 2.1357 |
| 0.6724 | 1.6257 | 0.7753 | 2.1925 |
| 0.7843 | 1.6813 | 0.8879 | 2.2312 |
| 0.9086 | 1.7174 | 0.9699 | 2.2482 |
| 0.9535 | 1.7347 | 5.7160 | 2.7394 |
| 5.8088 | 2.1726 | 10.5545 | 2.9061 |
| 10.4536 | 2.3161 | 15.8391 | 3.0116 |
| 15.7497 | 2.4213 | 20.5849 | 3.0807 |
| 20.6689 | 2.4874 | 25.7086 | 3.1410 |
| 25.6979 | 2.5416 | 30.7870 | 3.1885 |
| 30.8284 | 2.5866 | 35.7129 | 3.2327 |
| 35.7766 | 2.6265 | 40.7847 | 3.2755 |
| 40.8966 | 2.6598 | 45.8686 | 3.3070 |
| 45.7294 | 2.6968 | 50.7835 | 3.3357 |
| 50.8951 | 2.7253 | 55.8009 | 3.3643 |
| 55.7567 | 2.7518 | 60.8266 | 3.3919 |
| 60.8430 | 2.7788 | 65.9115 | 3.4198 |
| 65.8121 | 2.8051 | 70.8305 | 3.4457 |
| 70.8388 | 2.8304 | 75.8973 | 3.4728 |
| 75.8720 | 2.8549 | 80.8106 | 3.4981 |
| 80.8581 | 2.8786 | 85.8008 | 3.5226 |
| 85.8815 | 2.8976 | 90.8615 | 3.5411 |
| 90.8423 | 2.9158 | 95.8534 | 3.5585 |
| 95.8427 | 2.9377 | 99.8753 | 3.5793 |
| 99.8812 | 2.9543 | | |
| 308.15 K | | | |
| 0.0095 | 0.3693 | 0.0096 | 0.5194 |
| 0.0194 | 0.5382 | 0.0191 | 0.7469 |
| 0.0288 | 0.6418 | 0.0288 | 0.8885 |
| 0.0386 | 0.7217 | 0.0380 | 0.9867 |
| 0.0476 | 0.7732 | 0.0477 | 1.0611 |
| 0.0579 | 0.8222 | 0.0576 | 1.1252 |
| 0.0679 | 0.8594 | 0.0678 | 1.1890 |
| 0.0765 | 0.8940 | 0.0780 | 1.2378 |
| 0.0860 | 0.9239 | 0.0858 | 1.2692 |
| 0.0966 | 0.9512 | 0.0960 | 1.3059 |
| 0.1050 | 0.9741 | 0.1074 | 1.3472 |
| 0.1144 | 0.9958 | 0.1185 | 1.3774 |
| 0.1288 | 1.0252 | 0.1247 | 1.3937 |
| 0.1363 | 1.0408 | 0.1374 | 1.4308 |
| 0.1510 | 1.0626 | 0.1491 | 1.4570 |
| 0.1531 | 1.0696 | 0.1532 | 1.4701 |
| 0.1639 | 1.0869 | 0.1698 | 1.5080 |

**Table A.1. continued**

| Lewatit | | Purolite | |
|---|---|---|---|
| pressure (kPa) | loading (mmol $g^{-1}$) | pressure (kPa) | loading (mmol $g^{-1}$) |
| 308.15 K | | | |
| 0.1771 | 1.1145 | 0.1747 | 1.5187 |
| 0.1890 | 1.1354 | 0.1877 | 1.5417 |
| 0.1948 | 1.1476 | 0.1940 | 1.5545 |
| 0.2935 | 1.2550 | 0.2852 | 1.6644 |
| 0.3888 | 1.3290 | 0.3932 | 1.7959 |
| 0.4757 | 1.3913 | 0.5128 | 1.8790 |
| 0.5870 | 1.4463 | 0.5700 | 1.9119 |
| 0.6669 | 1.4839 | 0.6655 | 1.9597 |
| 0.7888 | 1.5244 | 0.7797 | 2.0147 |
| 0.8575 | 1.5508 | 0.8660 | 2.0558 |
| 0.9728 | 1.5828 | 0.9716 | 2.0956 |
| 5.8500 | 2.0758 | 5.7257 | 2.6257 |
| 10.7848 | 2.2267 | 10.8856 | 2.8169 |
| 15.8226 | 2.3233 | 15.8450 | 2.9188 |
| 20.6486 | 2.3933 | 20.5605 | 2.9896 |
| 25.7261 | 2.4533 | 25.7373 | 3.0523 |
| 30.7286 | 2.5030 | 30.7434 | 3.1050 |
| 35.8054 | 2.5444 | 35.7830 | 3.1477 |
| 40.8059 | 2.5825 | 40.8276 | 3.1888 |
| 45.8481 | 2.6195 | 45.8167 | 3.2266 |
| 50.8865 | 2.6491 | 50.8282 | 3.2562 |
| 55.8468 | 2.6762 | 55.8172 | 3.2835 |
| 60.8177 | 2.7014 | 60.8117 | 3.3112 |
| 65.8800 | 2.7283 | 65.8124 | 3.3385 |
| 70.9295 | 2.7526 | 70.8692 | 3.3638 |
| 75.9488 | 2.7758 | 75.8413 | 3.3898 |
| 80.9240 | 2.7979 | 80.8762 | 3.4141 |
| 85.9525 | 2.8195 | 85.8733 | 3.4383 |
| 90.8477 | 2.8406 | 90.8794 | 3.4627 |
| 95.8545 | 2.8600 | 95.8331 | 3.4857 |
| 99.8652 | 2.8756 | 99.8509 | 3.5041 |
| 333.15 K | | | |
| 0.0105 | 0.0315 | 0.0102 | 0.0563 |
| 0.0216 | 0.0659 | 0.0228 | 0.0924 |
| 0.0309 | 0.0977 | 0.0328 | 0.1263 |
| 0.0426 | 0.1297 | 0.0423 | 0.1618 |
| 0.0595 | 0.1694 | 0.0614 | 0.1860 |
| 0.0768 | 0.2063 | 0.0753 | 0.2318 |
| 0.0995 | 0.2518 | 0.0876 | 0.2546 |
| 0.1211 | 0.3013 | 0.1093 | 0.3062 |
| 0.1581 | 0.3572 | 0.1301 | 0.3492 |
| 0.1865 | 0.3884 | 0.1563 | 0.4045 |
| 0.2077 | 0.4265 | 0.1775 | 0.4269 |
| 0.3240 | 0.5252 | 0.2187 | 0.4591 |
| 0.4464 | 0.6099 | 0.3571 | 0.6143 |
| 0.5473 | 0.6771 | 0.5001 | 0.7322 |
| 0.7017 | 0.7449 | 0.6925 | 0.8665 |
| 0.8852 | 0.8215 | 0.8769 | 0.9507 |
| 1.1260 | 0.8959 | 1.0905 | 1.0371 |
| 6.3911 | 1.4741 | 5.9294 | 1.7544 |
| 10.8474 | 1.6498 | 10.7753 | 1.9954 |
| 15.6754 | 1.7779 | 15.7960 | 2.1451 |
| 20.6386 | 1.8808 | 20.5698 | 2.2438 |
| 25.9714 | 1.9379 | 26.0194 | 2.3273 |
| 30.9164 | 2.0076 | 30.7035 | 2.3938 |
| 35.9762 | 2.0504 | 35.7406 | 2.4712 |
| 40.6468 | 2.0929 | 40.9605 | 2.5071 |
| 45.7558 | 2.1371 | 45.6960 | 2.5504 |
| 333.15 K | | | |
| 50.9142 | 2.1666 | 50.8919 | 2.5899 |
| 55.5954 | 2.2108 | 55.6468 | 2.6424 |
| 60.8503 | 2.2451 | 60.9526 | 2.6804 |
| 65.9614 | 2.2577 | 65.7767 | 2.7095 |
| 70.6494 | 2.2863 | 71.0052 | 2.7156 |
| 75.6968 | 2.3247 | 75.8601 | 2.7419 |
| 80.9891 | 2.3465 | 80.9299 | 2.7683 |
| 85.6973 | 2.3648 | 85.7563 | 2.8187 |
| 90.6415 | 2.3969 | 91.2694 | 2.8117 |
| 96.1607 | 2.3951 | 95.6004 | 2.8597 |
| 99.5864 | 2.4313 | 100.1280 | 2.8605 |
| 343.15 K | | | |
| 0.0096 | 0.0118 | 0.0100 | 0.0187 |
| 0.0191 | 0.0236 | 0.0197 | 0.0347 |
| 0.0293 | 0.0364 | 0.0303 | 0.0523 |
| 0.0393 | 0.0497 | 0.0395 | 0.0658 |
| 0.0486 | 0.0606 | 0.0494 | 0.0812 |
| 0.0590 | 0.0734 | 0.0582 | 0.0943 |
| 0.0710 | 0.0887 | 0.0680 | 0.1076 |
| 0.0793 | 0.0984 | 0.0799 | 0.1254 |
| 0.0869 | 0.1080 | 0.0907 | 0.1392 |
| 0.0997 | 0.1238 | 0.0980 | 0.1482 |
| 0.1084 | 0.1339 | 0.1067 | 0.1605 |
| 0.1189 | 0.1443 | 0.1162 | 0.1718 |
| 0.1256 | 0.1526 | 0.1243 | 0.1835 |
| 0.1340 | 0.1617 | 0.1338 | 0.1948 |
| 0.1506 | 0.1766 | 0.1536 | 0.2152 |
| 0.1524 | 0.1827 | 0.1790 | 0.2448 |
| 0.1681 | 0.1951 | 0.1872 | 0.2587 |
| 0.1781 | 0.2066 | 0.2080 | 0.2800 |
| 0.1893 | 0.2167 | 0.2930 | 0.3620 |
| 0.1951 | 0.2231 | 0.3860 | 0.4376 |
| 0.2891 | 0.2965 | 0.4825 | 0.5105 |
| 0.3899 | 0.3642 | 0.5950 | 0.5776 |
| 0.4884 | 0.4185 | 0.6753 | 0.6251 |
| 0.5756 | 0.4625 | 0.7914 | 0.6868 |
| 0.6846 | 0.5085 | 0.8959 | 0.7337 |
| 0.7658 | 0.5460 | 0.9710 | 0.7744 |
| 0.9013 | 0.5845 | 5.7681 | 1.5482 |
| 1.0028 | 0.6163 | 10.7707 | 1.8393 |
| 5.9962 | 1.1988 | 15.9153 | 1.9927 |
| 10.6830 | 1.4051 | 20.4191 | 2.1072 |
| 15.6502 | 1.5307 | 25.8462 | 2.2038 |
| 20.6001 | 1.6202 | 30.7598 | 2.2644 |
| 25.7441 | 1.6974 | 35.6390 | 2.3310 |
| 31.0217 | 1.7584 | 40.8953 | 2.3796 |
| 35.9594 | 1.8051 | 45.7059 | 2.4304 |
| 40.9609 | 1.8470 | 50.9241 | 2.4683 |
| 46.0158 | 1.8873 | 55.6611 | 2.5150 |
| 51.0457 | 1.9197 | 60.9347 | 2.5494 |
| 55.9892 | 1.9534 | 65.7682 | 2.5842 |
| 60.8558 | 1.9852 | 70.9074 | 2.6135 |
| 65.8555 | 2.0126 | 75.9473 | 2.6355 |
| 70.8110 | 2.0366 | 80.8036 | 2.6671 |
| 75.8180 | 2.0599 | 85.9073 | 2.6934 |
| 80.8109 | 2.0862 | 90.8984 | 2.7195 |
| 85.8983 | 2.1069 | 95.9723 | 2.7431 |
| 90.9206 | 2.1292 | 99.8753 | 2.7572 |

**Table A.1. continued**

| Lewatit | | Purolite | |
|---|---|---|---|
| pressure (kPa) | loading (mmol $g^{-1}$) | pressure (kPa) | loading (mmol $g^{-1}$) |
| 343.15 K | | | |
| 95.9772 | 2.1494 | | |
| 99.7836 | 2.1674 | | |
| 353.15 K | | | |
| 0.0102 | 0.0048 | 0.0108 | 0.0071 |
| 0.0201 | 0.0094 | 0.0205 | 0.0136 |
| 0.0318 | 0.0152 | 0.0345 | 0.0201 |
| 0.0431 | 0.0195 | 0.0475 | 0.0290 |
| 0.0506 | 0.0236 | 0.0530 | 0.0310 |
| 0.0608 | 0.0304 | 0.0673 | 0.0372 |
| 0.0685 | 0.0353 | 0.0852 | 0.0488 |
| 0.0798 | 0.0402 | 0.0916 | 0.0553 |
| 0.0925 | 0.0449 | 0.1086 | 0.0628 |
| 0.1038 | 0.0516 | 0.1189 | 0.0693 |
| 0.1114 | 0.0553 | 0.1322 | 0.0753 |
| 0.1164 | 0.0584 | 0.1398 | 0.0796 |
| 0.1298 | 0.0650 | 0.1474 | 0.0852 |
| 0.1373 | 0.0682 | 0.1583 | 0.0902 |
| 0.1458 | 0.0726 | 0.1746 | 0.0974 |
| 0.1641 | 0.0803 | 0.1986 | 0.1092 |
| 0.1822 | 0.0917 | 0.2969 | 0.1566 |
| 0.2003 | 0.1012 | 0.3869 | 0.2001 |
| 0.3096 | 0.1490 | 0.4968 | 0.2437 |
| 0.3999 | 0.1882 | 0.6146 | 0.2890 |
| 0.4995 | 0.2239 | 0.6875 | 0.3193 |
| 0.5925 | 0.2623 | 0.8035 | 0.3589 |
| 0.6984 | 0.2932 | 0.9379 | 0.4134 |
| 0.8290 | 0.3356 | 1.0115 | 0.4350 |
| 0.8780 | 0.3572 | 6.0030 | 1.1619 |
| 1.0157 | 0.3904 | 10.8743 | 1.4501 |
| 5.7338 | 0.9153 | 15.7532 | 1.6196 |
| 10.6990 | 1.1304 | 20.4132 | 1.7566 |
| 15.5992 | 1.2670 | 25.9373 | 1.8788 |
| 20.6210 | 1.3763 | 30.9714 | 1.9412 |
| 26.0789 | 1.4584 | 35.4240 | 2.0302 |
| 30.7968 | 1.5205 | 41.1392 | 2.0687 |
| 36.0010 | 1.5726 | 45.5553 | 2.1251 |
| 40.9155 | 1.6152 | 51.0031 | 2.1901 |
| 45.9313 | 1.6599 | 56.0666 | 2.2277 |
| 50.9780 | 1.6972 | 60.9632 | 2.2655 |
| 56.0260 | 1.7311 | 65.8467 | 2.2962 |
| 60.8200 | 1.7576 | 70.9503 | 2.3408 |
| 65.8187 | 1.7837 | 76.0414 | 2.3697 |
| 70.9114 | 1.8099 | 80.8371 | 2.3865 |
| 75.7575 | 1.8398 | 85.6448 | 2.4258 |
| 80.8261 | 1.8667 | 90.9643 | 2.4442 |
| 85.7536 | 1.8937 | 95.7065 | 2.4712 |
| 91.0136 | 1.9162 | 99.9510 | 2.4960 |
| 95.8590 | 1.9336 | | |
| 99.8691 | 1.9497 | | |

| Lewatit | | Purolite | |
|---|---|---|---|
| pressure (kPa) | loading (mmol $g^{-1}$) | pressure (kPa) | loading (mmol $g^{-1}$) |
| 393.15 K | | | |
| 0.0105 | 0.0001 | 0.0101 | 0.0003 |
| 0.0197 | 0.0002 | 0.0202 | 0.0006 |
| 0.0300 | 0.0003 | 0.0299 | 0.0009 |
| 0.0397 | 0.0003 | 0.0397 | 0.0012 |
| 0.0503 | 0.0004 | 0.0499 | 0.0015 |
| 0.0598 | 0.0005 | 0.0600 | 0.0018 |
| 0.0699 | 0.0006 | 0.0696 | 0.0021 |
| 0.0804 | 0.0007 | 0.0797 | 0.0024 |
| 0.0898 | 0.0008 | 0.0897 | 0.0026 |
| 0.0997 | 0.0009 | 0.0994 | 0.0029 |
| 0.1100 | 0.0009 | 0.1098 | 0.0032 |
| 0.1199 | 0.0010 | 0.1197 | 0.0035 |
| 0.1298 | 0.0011 | 0.1294 | 0.0038 |
| 0.1397 | 0.0012 | 0.1398 | 0.0040 |
| 0.1500 | 0.0013 | 0.1493 | 0.0043 |
| 0.1595 | 0.0013 | 0.1600 | 0.0046 |
| 0.1697 | 0.0014 | 0.1697 | 0.0048 |
| 0.1795 | 0.0015 | 0.1787 | 0.0052 |
| 0.1897 | 0.0016 | 0.1899 | 0.0054 |
| 0.1995 | 0.0017 | 0.1993 | 0.0056 |
| 0.2919 | 0.0028 | 0.2875 | 0.0085 |
| 0.3987 | 0.0041 | 0.3962 | 0.0118 |
| 0.4978 | 0.0053 | 0.4986 | 0.0148 |
| 0.5984 | 0.0065 | 0.5968 | 0.0180 |
| 0.6984 | 0.0077 | 0.6991 | 0.0211 |
| 0.7968 | 0.0090 | 0.7987 | 0.0238 |
| 0.8979 | 0.0102 | 0.8927 | 0.0271 |
| 0.9970 | 0.0114 | 0.9962 | 0.0303 |
| 5.9863 | 0.0846 | 5.8878 | 0.1789 |
| 10.8709 | 0.1629 | 10.9627 | 0.3230 |
| 15.8669 | 0.2397 | 15.8519 | 0.4545 |
| 20.8384 | 0.3139 | 20.9273 | 0.5642 |
| 25.8895 | 0.3773 | 25.8939 | 0.6693 |
| 30.8947 | 0.4369 | 30.9967 | 0.7564 |
| 36.1284 | 0.4885 | 35.9152 | 0.8401 |
| 40.9355 | 0.5358 | 41.0787 | 0.9119 |
| 45.7619 | 0.5755 | 45.8427 | 0.9846 |
| 50.7733 | 0.6173 | 51.1559 | 1.0417 |
| 55.9635 | 0.6572 | 55.9456 | 1.0932 |
| 60.8472 | 0.6922 | 61.0486 | 1.1470 |
| 65.8051 | 0.7258 | 66.0937 | 1.1955 |
| 70.8430 | 0.7566 | 71.1014 | 1.2331 |
| 76.0314 | 0.7881 | 75.9221 | 1.2812 |
| 81.1035 | 0.8137 | 81.1226 | 1.3139 |
| 85.7548 | 0.8421 | 85.9784 | 1.3566 |
| 90.8563 | 0.8687 | 91.1966 | 1.3891 |
| 95.8665 | 0.8924 | 95.8046 | 1.4219 |
| 99.7840 | 0.9163 | 99.9060 | 1.4502 |

**Table A.2. Adsorbed Amounts of $N_2$ on Lewatit and Purolite at 288, 298, and 308 K up to 100 kPa and of Ar and $O_2$ at 298 K up to 100 kPa**

| Lewatit | | Purolite | |
|---|---|---|---|
| pressure (kPa) | loading (mmol $g^{-1}$) | pressure (kPa) | loading (mmol $g^{-1}$) |
| $N_2$, 288.15 K | | | |
| 4.9705 | 0.0004 | 4.9770 | 0.0003 |
| 9.9619 | 0.0007 | 9.9411 | 0.0007 |
| 14.9410 | 0.0011 | 14.9307 | 0.0011 |
| 19.9003 | 0.0015 | 19.8935 | 0.0014 |
| 24.9313 | 0.0019 | 24.8925 | 0.0018 |
| 29.8587 | 0.0022 | 29.8783 | 0.0021 |
| 34.9014 | 0.0026 | 34.9064 | 0.0024 |
| 39.8773 | 0.0029 | 39.8799 | 0.0028 |
| 44.9095 | 0.0033 | 44.8721 | 0.0031 |
| 49.9241 | 0.0036 | 49.8972 | 0.0034 |
| 54.8507 | 0.0039 | 54.8451 | 0.0037 |
| 59.8509 | 0.0043 | 59.9643 | 0.0041 |
| 64.8614 | 0.0047 | 64.9073 | 0.0044 |
| 69.9391 | 0.0051 | 69.8591 | 0.0047 |
| 74.9633 | 0.0055 | 74.9238 | 0.0050 |
| 79.9295 | 0.0059 | 79.8573 | 0.0053 |
| 84.9315 | 0.0063 | 84.9280 | 0.0056 |
| 89.8660 | 0.0067 | 89.8931 | 0.0060 |
| 94.9100 | 0.0070 | 94.9111 | 0.0062 |
| 99.9499 | 0.0075 | 99.8821 | 0.0066 |
| $N_2$, 298.15 K | | | |
| 4.9842 | 0.0003 | 4.9746 | 0.0003 |
| 9.9522 | 0.0006 | 9.9465 | 0.0006 |
| 14.9971 | 0.0010 | 14.9152 | 0.0009 |
| 19.9005 | 0.0013 | 19.8947 | 0.0012 |
| 24.8843 | 0.0015 | 24.8964 | 0.0015 |
| 29.8770 | 0.0018 | 29.8589 | 0.0017 |
| 34.9458 | 0.0020 | 34.9106 | 0.0020 |
| 39.8862 | 0.0023 | 39.8584 | 0.0023 |
| 44.8807 | 0.0026 | 44.9108 | 0.0025 |
| 49.8500 | 0.0028 | 49.9112 | 0.0027 |
| 54.9833 | 0.0031 | 54.8566 | 0.0030 |
| 59.9333 | 0.0034 | 59.8670 | 0.0033 |
| 64.8623 | 0.0037 | 64.8482 | 0.0035 |
| 69.9484 | 0.0040 | 69.8431 | 0.0037 |
| 74.8743 | 0.0043 | 74.8580 | 0.0040 |
| 79.8735 | 0.0046 | 79.8336 | 0.0042 |
| 84.9031 | 0.0050 | 84.8494 | 0.0045 |
| 89.8751 | 0.0053 | 89.9657 | 0.0047 |
| 94.8786 | 0.0056 | 94.8946 | 0.0049 |
| 99.9605 | 0.0060 | 99.9185 | 0.0052 |
| $N_2$, 308.15 K | | | |
| 4.9786 | 0.0003 | 4.9759 | 0.0002 |
| 9.9422 | 0.0005 | 9.9522 | 0.0005 |
| 14.9405 | 0.0007 | 14.9357 | 0.0007 |
| 19.9531 | 0.0009 | 19.9411 | 0.0010 |
| 24.9008 | 0.0011 | 24.9004 | 0.0012 |
| 29.9436 | 0.0013 | 29.8893 | 0.0014 |
| 34.9239 | 0.0015 | 34.9033 | 0.0016 |
| 39.9056 | 0.0018 | 39.8638 | 0.0018 |
| 44.8638 | 0.0020 | 44.8367 | 0.0020 |
| 49.9578 | 0.0021 | 49.8619 | 0.0022 |

| Lewatit | | Purolite | |
|---|---|---|---|
| pressure (kPa) | loading (mmol $g^{-1}$) | pressure (kPa) | loading (mmol $g^{-1}$) |
| $N_2$, 308.15 K | | | |
| 54.8940 | 0.0023 | 54.9043 | 0.0024 |
| 59.8650 | 0.0025 | 59.9672 | 0.0026 |
| 64.8970 | 0.0027 | 64.8946 | 0.0028 |
| 69.9895 | 0.0029 | 69.8802 | 0.0029 |
| 74.9522 | 0.0031 | 74.9274 | 0.0031 |
| 79.8775 | 0.0033 | 79.9741 | 0.0033 |
| 84.8909 | 0.0036 | 84.9072 | 0.0035 |
| 89.9034 | 0.0039 | 89.8630 | 0.0037 |
| 94.8618 | 0.0042 | 94.8532 | 0.0039 |
| 99.8949 | 0.0045 | 99.9574 | 0.0041 |
| Ar, 298.15 K | | | |
| 4.9589 | 0.0008 | 4.9183 | 0.0011 |
| 9.9175 | 0.0014 | 9.9138 | 0.0018 |
| 14.9131 | 0.0021 | 14.8353 | 0.0024 |
| 19.9124 | 0.0027 | 20.0806 | 0.0031 |
| 24.8784 | 0.0033 | 25.0577 | 0.0036 |
| 29.8748 | 0.0039 | 30.1310 | 0.0042 |
| 34.8881 | 0.0045 | 35.0868 | 0.0048 |
| 39.8557 | 0.0050 | 40.0887 | 0.0053 |
| 44.9021 | 0.0056 | 45.0853 | 0.0059 |
| 49.8463 | 0.0062 | 50.0910 | 0.0064 |
| 54.8800 | 0.0067 | 55.1456 | 0.0069 |
| 59.8644 | 0.0073 | 60.1690 | 0.0074 |
| 64.9516 | 0.0079 | 65.0656 | 0.0079 |
| 69.8489 | 0.0085 | 70.1693 | 0.0084 |
| 74.9445 | 0.0090 | 75.0462 | 0.0089 |
| 80.0407 | 0.0096 | 80.0425 | 0.0094 |
| 84.9087 | 0.0102 | 85.1690 | 0.0099 |
| 89.9196 | 0.0108 | 90.1552 | 0.0103 |
| 94.9831 | 0.0114 | 94.9081 | 0.0108 |
| 99.9133 | 0.0120 | 99.8671 | 0.0113 |
| $O_2$, 298.15 K | | | |
| 4.9320 | 0.0010 | 4.8460 | 0.0023 |
| 9.9826 | 0.0017 | 9.8716 | 0.0030 |
| 15.0286 | 0.0024 | 15.0624 | 0.0036 |
| 20.0402 | 0.0030 | 20.0684 | 0.0041 |
| 25.0175 | 0.0036 | 25.0996 | 0.0048 |
| 29.9954 | 0.0042 | 30.1362 | 0.0053 |
| 35.0070 | 0.0048 | 35.1475 | 0.0059 |
| 39.9963 | 0.0054 | 40.1525 | 0.0065 |
| 45.0186 | 0.0061 | 45.1683 | 0.0071 |
| 50.0008 | 0.0067 | 50.1693 | 0.0077 |
| 54.9991 | 0.0073 | 55.1370 | 0.0083 |
| 60.0810 | 0.0079 | 60.1264 | 0.0089 |
| 65.1048 | 0.0086 | 65.1358 | 0.0095 |
| 70.1306 | 0.0092 | 70.1374 | 0.0101 |
| 75.0625 | 0.0098 | 75.1198 | 0.0107 |
| 80.1238 | 0.0104 | 79.8973 | 0.0114 |
| 85.1139 | 0.0111 | 84.8468 | 0.0120 |
| 90.1541 | 0.0117 | 89.9351 | 0.0127 |
| 95.1448 | 0.0124 | 95.0208 | 0.0135 |
| 99.8556 | 0.0130 | 100.0578 | 0.0142 |

Table A.3. Adsorbed Amounts of $H_2O$ on Lewatit and Purolite at 288 K up to 1.6 kPa and at 298 and 308 K up to 2.3 kPa

| Lewatit | | Purolite | |
|---|---|---|---|
| pressure (kPa) | loading (mmol/g) | pressure (kPa) | loading (mmol/g) |
| 288.15 K | | | |
| 0.0242 | 0.0963 | 0.0241 | 0.1571 |
| 0.0476 | 0.1716 | 0.0484 | 0.2785 |
| 0.0731 | 0.2529 | 0.0726 | 0.3924 |
| 0.0983 | 0.3289 | 0.0986 | 0.5075 |
| 0.1218 | 0.4006 | 0.1231 | 0.6133 |
| 0.1461 | 0.4720 | 0.1498 | 0.7219 |
| 0.1712 | 0.5395 | 0.1712 | 0.8145 |
| 0.1952 | 0.6067 | 0.1975 | 0.9189 |
| 0.2198 | 0.6706 | 0.2222 | 1.0156 |
| 0.2433 | 0.7348 | 0.2472 | 1.1163 |
| 0.2663 | 0.7963 | 0.2708 | 1.2134 |
| 0.2903 | 0.8582 | 0.2957 | 1.3106 |
| 0.3121 | 0.9186 | 0.3194 | 1.4050 |
| 0.3515 | 1.0328 | 0.3422 | 1.4993 |
| 0.3762 | 1.0992 | 0.3769 | 1.6498 |
| 0.3962 | 1.1559 | 0.4020 | 1.7503 |
| 0.4136 | 1.2198 | 0.4213 | 1.8446 |
| 0.4330 | 1.2826 | 0.4439 | 1.9421 |
| 0.4564 | 1.3460 | 0.4653 | 2.0376 |
| 0.4965 | 1.4702 | 0.4876 | 2.1331 |
| 0.5263 | 1.5896 | 0.5319 | 2.3268 |
| 0.6024 | 1.8263 | 0.6008 | 2.6833 |
| 0.6287 | 1.9281 | 0.6675 | 3.1241 |
| 0.6916 | 2.2131 | 0.7324 | 3.5643 |
| 0.7648 | 2.5078 | 0.7844 | 4.0022 |
| 0.8316 | 2.8074 | 0.8440 | 4.4550 |
| 0.8748 | 3.0928 | 0.8980 | 4.8973 |
| 0.9422 | 3.3690 | 0.9507 | 5.3341 |
| 0.9526 | 3.4627 | 1.0094 | 5.7739 |
| 1.0044 | 3.7446 | 1.0464 | 6.2130 |
| 1.0936 | 4.3253 | 1.0966 | 6.6306 |
| 1.1859 | 4.8748 | 1.1499 | 7.0571 |
| 1.2059 | 5.1502 | 1.2140 | 7.9253 |
| 1.2412 | 5.4202 | 1.2470 | 8.3328 |
| 1.3019 | 5.9595 | 1.2997 | 9.1272 |
| 1.3333 | 6.2286 | 1.3682 | 9.9137 |
| 1.3864 | 6.7417 | 1.3803 | 10.2964 |
| 1.4265 | 7.2669 | 1.4317 | 11.0719 |
| 1.4918 | 8.2589 | 1.4860 | 12.2120 |
| 1.5258 | 8.9717 | 1.5290 | 13.3252 |
| 298.15 K | | | |
| 0.0240 | 0.0455 | 0.0244 | 0.0781 |
| 0.0484 | 0.0867 | 0.0487 | 0.1449 |
| 0.0734 | 0.1322 | 0.0744 | 0.2169 |
| 0.0976 | 0.1743 | 0.0998 | 0.2848 |
| 0.1225 | 0.2164 | 0.1235 | 0.3458 |
| 0.1469 | 0.2556 | 0.1489 | 0.4085 |
| 0.1714 | 0.2952 | 0.1732 | 0.4682 |
| 0.1956 | 0.3325 | 0.1995 | 0.5283 |
| 0.2197 | 0.3699 | 0.2227 | 0.5804 |
| 0.2429 | 0.4058 | 0.2480 | 0.6383 |
| 0.2670 | 0.4417 | 0.2743 | 0.6942 |
| 0.2903 | 0.4767 | 0.2985 | 0.7438 |
| 0.3153 | 0.5118 | 0.3235 | 0.7941 |
| 0.3388 | 0.5463 | 0.3464 | 0.8434 |
| 0.3618 | 0.5812 | 0.3706 | 0.8932 |
| 0.3874 | 0.6159 | 0.3949 | 0.9420 |
| 0.4130 | 0.6506 | 0.4195 | 0.9916 |

| Lewatit | | Purolite | |
|---|---|---|---|
| pressure (kPa) | loading (mmol/g) | pressure (kPa) | loading (mmol/g) |
| 298.15 K | | | |
| 0.4381 | 0.6847 | 0.4452 | 1.0407 |
| 0.4628 | 0.7192 | 0.4706 | 1.0896 |
| 0.4875 | 0.7535 | 0.4958 | 1.1387 |
| 0.5377 | 0.8236 | 0.5419 | 1.2405 |
| 0.5878 | 0.8909 | 0.5741 | 1.3425 |
| 0.6386 | 0.9586 | 0.6560 | 1.5442 |
| 0.6845 | 1.0244 | 0.6760 | 1.6223 |
| 0.7253 | 1.0909 | 0.7468 | 1.8130 |
| 0.7877 | 1.2217 | 0.7757 | 1.8922 |
| 0.8555 | 1.3420 | 0.8566 | 2.0733 |
| 0.9189 | 1.4674 | 0.9244 | 2.2373 |
| 0.9743 | 1.5870 | 0.9924 | 2.4107 |
| 1.0300 | 1.7062 | 1.0059 | 2.5631 |
| 1.0856 | 1.8194 | 1.0621 | 2.7355 |
| 1.1484 | 1.9317 | 1.0953 | 2.8316 |
| 1.2111 | 2.0441 | 1.1498 | 3.0072 |
| 1.3412 | 2.3085 | 1.2501 | 3.3939 |
| 1.4071 | 2.5738 | 1.3458 | 3.7512 |
| 1.5232 | 2.8235 | 1.4418 | 4.1852 |
| 1.6307 | 3.1670 | 1.5340 | 4.5612 |
| 1.7100 | 3.4090 | 1.6343 | 5.2398 |
| 1.7747 | 3.6444 | 1.6846 | 5.5937 |
| 1.8485 | 3.8706 | 1.7530 | 5.9282 |
| 1.9098 | 4.0582 | 1.8082 | 6.2656 |
| 1.9720 | 4.2849 | 1.8725 | 6.5944 |
| 2.0388 | 4.5027 | 1.9247 | 6.9304 |
| 2.0672 | 4.9442 | 1.9782 | 7.2538 |
| 2.1128 | 5.1477 | 2.0293 | 7.5751 |
| 2.1480 | 5.3609 | 2.0757 | 7.8259 |
| 2.1858 | 5.5652 | 2.1220 | 8.1421 |
| | | 2.1633 | 8.4757 |
| | | 2.2132 | 8.7949 |
| 308.15 K | | | |
| 0.0245 | 0.0247 | 0.0245 | 0.0428 |
| 0.0479 | 0.0470 | 0.0481 | 0.0797 |
| 0.0733 | 0.0741 | 0.0749 | 0.1245 |
| 0.0976 | 0.0991 | 0.1002 | 0.1666 |
| 0.1219 | 0.1244 | 0.1254 | 0.2060 |
| 0.1460 | 0.1482 | 0.1486 | 0.2401 |
| 0.1708 | 0.1721 | 0.1728 | 0.2757 |
| 0.1954 | 0.1950 | 0.1976 | 0.3106 |
| 0.2202 | 0.2180 | 0.2220 | 0.3454 |
| 0.2449 | 0.2404 | 0.2470 | 0.3799 |
| 0.2699 | 0.2627 | 0.2728 | 0.4141 |
| 0.2939 | 0.2846 | 0.2983 | 0.4480 |
| 0.3191 | 0.3064 | 0.3235 | 0.4817 |
| 0.3436 | 0.3280 | 0.3481 | 0.5151 |
| 0.3686 | 0.3496 | 0.3746 | 0.5482 |
| 0.3927 | 0.3710 | 0.3971 | 0.5771 |
| 0.4172 | 0.3926 | 0.4222 | 0.6100 |
| 0.4431 | 0.4141 | 0.4477 | 0.6428 |
| 0.4685 | 0.4356 | 0.4684 | 0.6762 |
| 0.4936 | 0.4570 | 0.5139 | 0.7303 |
| 0.5227 | 0.4814 | 0.5500 | 0.7852 |
| 0.5843 | 0.5351 | 0.5933 | 0.8411 |
| 0.6392 | 0.5866 | 0.6359 | 0.8984 |
| 0.7008 | 0.6371 | 0.6829 | 0.9587 |
| 0.7284 | 0.6720 | 0.7330 | 1.0219 |

**Table A.3. continued**

| Lewatit | | Purolite | |
|---|---|---|---|
| pressure (kPa) | loading (mmol/g) | pressure (kPa) | loading (mmol/g) |
| 308.15 K | | | |
| 0.7840 | 0.7212 | 0.7834 | 1.0864 |
| 0.8283 | 0.7595 | 0.8398 | 1.1737 |
| 0.8833 | 0.8079 | 0.8975 | 1.2580 |
| 0.9376 | 0.8556 | 0.9423 | 1.3282 |
| 0.9902 | 0.9025 | 1.0003 | 1.4093 |
| 1.0274 | 0.9499 | 1.0529 | 1.4971 |
| 1.0722 | 0.9976 | 1.1172 | 1.5888 |
| 1.1273 | 1.0447 | 1.1650 | 1.6825 |
| 1.1701 | 1.0925 | 1.2356 | 1.7747 |
| 1.2172 | 1.1421 | 1.2984 | 1.8759 |
| 1.2706 | 1.1928 | 1.3572 | 1.9808 |
| 1.3217 | 1.2439 | 1.4214 | 2.0858 |
| 1.3650 | 1.2956 | 1.4656 | 2.1900 |

| Lewatit | | Purolite | |
|---|---|---|---|
| pressure (kPa) | loading (mmol/g) | pressure (kPa) | loading (mmol/g) |
| 308.15 K | | | |
| 1.4193 | 1.3482 | 1.5190 | 2.2949 |
| 1.4562 | 1.4024 | 1.5609 | 2.4001 |
| 1.5038 | 1.4582 | 1.6159 | 2.5056 |
| 1.5585 | 1.5152 | 1.6785 | 2.6210 |
| 1.5980 | 1.5738 | 1.8172 | 2.9714 |
| 1.6443 | 1.6361 | 1.9388 | 3.3142 |
| 1.6909 | 1.7011 | 2.0300 | 3.6118 |
| 1.7397 | 1.7683 | 2.1514 | 3.8972 |
| 1.7908 | 1.8388 | 2.2372 | 4.1964 |
| 1.8380 | 1.9124 | | |
| 1.9730 | 2.1231 | | |
| 2.1095 | 2.3400 | | |
| 2.2403 | 2.5458 | | |
